# When and what to test for: A cost-effectiveness analysis of febrile illness test-and-treat strategies in the era of responsible antibiotic use

**DOI:** 10.1371/journal.pone.0227409

**Published:** 2020-01-08

**Authors:** Anthony Zhenhuan Zhang, Diana Negoescu, Claudia Munoz-Zanzi

**Affiliations:** 1 College of Science and Engineering, Industrial and System Engineering, University of Minnesota, Minneapolis, Minnesota, United States of America; 2 School of Public Health, Division of Environmental Health Sciences, University of Minnesota, Minneapolis, Minnesota, United States of America; Universita degli Studi di Parma, ITALY

## Abstract

**Background:**

Febrile illness caused by viral and bacterial diseases (e.g., dengue and leptospirosis) often have similar symptoms and are difficult to differentiate without diagnostic tests. If not treated appropriately, patients may experience serious complications. The question of what diagnostic tests to make available to providers in order to inform antibiotic therapy remains an open problem for health services facing limited resources.

**Methods and findings:**

We formulated the problem of minimizing the weighted average of antibiotic underuse and overuse to inform the optimal diagnostic test and antibiotic treatment options for given occurrence probabilities of several bacterial and viral infections. We modeled the weight of antibiotic overuse as a monetary penalty per unnecessarily administered course, which we varied in both the base case and sensitivity analysis. Detailed Markov cohort models of febrile illness progression were used to estimate the weight of antibiotic underuse. The model accounted for multiple infections simultaneously and incorporated test, treatment, and other direct and indirect costs, as well as the effect of delays in seeking care and test turnaround times. We used the Markov models to numerically estimate disability-adjusted life years (DALYs), pre-penalty costs, and likelihood of antibiotics overuse per patient for fifteen different strategies in two example settings in Thailand, one with a higher probability of bacterial infections (Northern Thailand, Scenario A) and one with a higher probability of viral infections (Bangkok, Scenario B). We found that empirical antibiotic treatment to all patients always incurs the lowest pre-penalty cost (Scenario A: $47.5/patient, $100.6/patient, $149.5/patient for patients seeking care on day one, day four, and day ten respectively; Scenario B: $94.1/patient, $108.7/patient, $122.1/patient on day one, day four, and day ten respectively), and the lowest DALYs, (Scenario A: 0.2 DALYs/patient, 0.9 DALYs/patient, 1.7 DALYs/patient on day one, day four, and day ten, respectively; Scenario B: 0.5 DALYs/patient, 0.7 DALYs/patient, 0.9 DALYs/patient on day one, day four, and day ten, respectively). However, such strategy resulted in the highest proportion of antibiotic overuse per patient (Scenario A: 38.1%, 19.3%, 7.5% on day one, day four, and day ten, respectively; Scenario B: 82.9%, 42.1%, 16.3% on day one, day four, and day ten, respectively). Consequently, empirical antibiotic treatment became suboptimal with antibiotic overuse penalties above $12,800/course, $18,400/course, $23,900/course for patients presenting on day one, day four, and day ten in Scenario A and above $1,100/course, $1,500/course, $1,600/course for patients presenting on day one, day four, and day ten in Scenario B.

**Conclusions:**

Empirical antibiotic treatment to all patients provided the best outcomes if antibiotic overuse was not the primary concern or if presenting with viral disease (such as dengue) was unlikely. Empirical antibiotic treatment to severe patients only was in most cases not beneficial. Otherwise, strategies involving diagnostic tests became optimal. In particular, our results indicated that single test strategies (bacterial RDT or viral PCR) were optimal in regions with a greater probability of presenting with viral infection. PCR-led strategies (e.g., parallel bacterial PCR, or multiplex PCR) are robust under parameter uncertainty (e.g., with uncertain disease occurrence probabilities).

## Introduction

Clinical manifestations such as the sudden onset of acute fever, chills, and headache are the common features among several tropical and emerging diseases, including dengue, Zika, yellow fever, leptospirosis and scrub typhus [1]. Depending on the specific etiology, if untreated, patients could experience respiratory distress, multi-organ failure, and even death [2, 3]. Due to climate change and the increasing counts of weather-related disasters [4–6], healthcare providers are facing a growing number of febrile illness patients, especially in low-resource settings [7]. The global annual burden of diseases under the umbrella of acute febrile illness is estimated to be on the order of millions of cases [8, 9].

There are currently no evidence-based guidelines to inform diagnostic and treatment strategies and the necessary health care investments [10]. Early diagnosis of bacterial infection is essential since antibiotic therapy is more beneficial when initiated early [2]. Moreover, early diagnosis could avoid unnecessary antibiotic administration to patients with non-bacterial infections such as dengue [11]. Due to limited access to laboratory services, low quality of test results and prohibitively expensive diagnostic test costs, current World Health Organization (WHO) guidelines recommend for malaria-endemic, resource-limited regions the use of rapid malaria screening, and prescribing antibiotics to those with clinical signs of severe illness or specific bacterial infections [12]. Studies have also shown that empirical antibiotic prescription to suspected leptospirosis patients is cost-effective when not considering antibiotic overuse [13].

Nevertheless, antibiotic overuse has been recognized as a key driver of antibiotic resistance development, which is an increasingly pressing public health issue. In the U.S. alone, antibiotic resistance causes more than 2 million infections and 23,000 deaths per year [14]. A U.S. based study estimated excess hospital charges due to the presence of antimicrobial-resistant organism (i.e., methicillin-resistant S. aureus) was, on average, $ 86,400 per case (after Consumer Price Index inflation adjustment) [15]. The societal impact due to the transmission of antimicrobial-resistant organisms might still be underestimated in this case [16, 17].

As pointed out by Crump et al. [18], the literature on comprehensive cost-effectiveness assessment of clinical management strategies for non-malarial acute febrile illnesses is scarce. Most existing research in the domain of assessing the cost-effectiveness of protocols managing acute febrile illness has focused on either a single cause of infection or a specific strategy. Wangrangsimakul et al. [19] conducted a prospective observational study to investigate the causes of acute undifferentiated fever and concluded that pathogen-specific rapid diagnostic tests could inform the correct use of antibiotics and improve antimicrobial stewardship in their setting. Under the same stream of work, Lubell et al. [20] conducted a cost-effectiveness analysis of the management of scrub typhus and dengue in a rural Laos setting comparing the approaches of using pathogen-specific diagnostics and biomarker tests. However, potentially critical factors such as disease progression, health-seeking behaviors, and illness-stage-dependent diagnostic test accuracy have not been examined fully. On the other hand, studies have shown that empirical antibiotic administration might be the most cost-effective strategy in resource-limited settings when treating suspected leptospirosis patients [13]. However, this may lead to poor outcomes for non-bacterial infections, which are subsequently treated inappropriately with antibiotics [12]. Moreover, antibiotic overuse has become a significant health issue worldwide. As a consequence, the extent to which diagnostic and treatment strategies may be cost-effective under given scenarios remains uncertain.

The objective of this study was to inform the optimal diagnosis and antibiotic treatment strategies for patients with undifferentiated febrile illnesses. We determined the optimal decision by considering the tradeoff between antibiotic underuse (i.e., higher disability-adjusted life years (DALYs) and health care cost) and antibiotic overuse (i.e., unnecessary courses of antibiotics prescribed). We selected Thailand as an exemplary setting because it is endemic for several pathogens of interest, including dengue, leptospirosis, and scrub typhus at varying occurrence probabilities and immediate availability of published data for model development [1, 21].

## Methods

### Overview

To understand the degree to which different strategies achieve the balance between antibiotic overuse and underuse, we seek to quantify the expected consequences and proportions of patients falling in both categories for each strategy. To capture the consequences of antibiotic underuse, we developed a Markov cohort model that describes febrile disease progression with and without treatment. The long-term consequences of antibiotic overuse, such as the development of bacterial resistance, were much more challenging to estimate. Consequently, we assigned a monetary penalty (weight) to every patient unnecessarily prescribed a course of antibiotics, given the disease etiology, in order to capture how different values for this weight might drive optimal decision-making.

### Balancing antibiotic overuse and underuse

We first considered a simplified setting where patients presenting with fever had one of four possible disease etiologies: 1) a specific bacterial infection for which a diagnostic test exists and is treatable with a commonly prescribed antibiotic (i.e., doxycycline [12, 13]); 2) other bacterial infections for which no test is available, but also treatable with the same antibiotic; 3) a specific viral infection for which a test exists and it is not treatable with the antibiotic; 4) other infections for which there is no test and are not treatable with the antibiotic.

In this setting, we considered three strategies: 1) empirical antibiotic treatment (prescribing antibiotics to all patients, without any tests); 2) testing all patients using the bacterial infection test, and prescribing antibiotics to patients with positive results; 3) testing all patients using the viral infection test, and prescribing antibiotics to patients with negative results.

If the penalty (weight) for each patient with antibiotic-treatable (AT) bacterial disease who is not prescribed antibiotics is *w*_*under*_, and the penalty (weight) for each patient with non-antibiotic-treatable (NAT) disease who is prescribed antibiotics is *w*_*over*_, then we choose a strategy that minimizes the expected total penalty per patient:

$$w_{under}\;Prob\left(no\;antibiotics,\;AT\;disease\right)+w_{over}\;Prob(antibiotics,\;NAT\;disease)$$

The first and second term corresponds to antibiotic underuse and overuse penalty, respectively, associated with each strategy. We rewrite this objective as

$$w_{under}Prob(under)+w_{over}Prob(over)$$ (1)

We derived analytical expressions of antibiotic overuse and underuse probabilities for each strategy, estimated the weights (S1 Text, Section 2), and evaluated all three generic strategies using the objective defined in Eq (1) in order to understand the role that overuse penalties, along with disease probabilities of occurrence and test accuracy parameters, play into determining the best strategy among the three options. Notably, we found simple conditions involving only the probabilities of disease occurrence and the ratio *w*_*under*_ */ w*_*over*_ that guarantee that administering a specific test leads to a lower total penalty than administering the other test or no test at all (and administering antibiotics to all patients).

### Viral- versus bacterial-endemic settings: a case study for Thailand

After gaining intuition from analyzing the simplified version of the balancing problem, we turned to a more realistic setting in order to account for multiple disease scenarios, incorporate test, treatment and other direct and indirect costs, as well as the effect of delays in seeking care and in test turnaround times (TAT).

We categorized sources of infections into the following five types based on whether they are bacterial or viral, and whether they are treatable with antibiotic: 1) Leptospirosis (bacterial, treatable with the antibiotic); 2) Scrub typhus (bacterial, treatable with antibiotic); 3) “Other bacterial”, treatable with antibiotic, such as spotted fever group rickettsioses [13]; 4) Dengue (viral, not treatable with antibiotic); and 5) All other possible causes of infection as a general category of “others” which are assumed not to be treatable with antibiotic. We assumed that all cases were malaria negative, which is consistent with current practices of screening with highly accurate rapid tests for malaria first [22–24].

We selected two contrasting settings in Thailand with varying infection occurrence probabilities (Table 1): Northern Thailand, where leptospirosis, scrub typhus (bacterial infections) are more endemic–Scenario A [1], and Bangkok, where dengue (viral) is the most common cause of acute fever–Scenario B [21].

**Table 1:** Probabilities of common causes of febrile diseases among hospital patients in Thailand in selected settings with contrasting occurrence probabilities (obtained from [1, 21]).

Scenarios	Leptospirosis	Scrub typhus	Other bacterial	Dengue	Others
A: Bacterial disease is predominant (Northern Thailand)	52.8%	4.6%	4.5%	12.2%	25.9%
B: Viral disease is predominant (Bangkok, Thailand)	6.8%	1.7%	8.6%	67.1%	15.8%

### The disease progression model

We developed detailed Markov cohort models to capture disease progression for each febrile disease and used the model outcomes to evaluate alternative strategies for diagnosis and antibiotic treatment. We modeled a hypothetical cohort of 40-year-old adult patients (age varied in sensitivity analysis) with acute, undifferentiated fever since symptom onset. We set the time horizon to 45 days since most patients would be either recovered or deceased by then. We tracked patient health states daily. On each day, patients may recover, progress to severe disease stage or die. We considered patients presenting to hospitals on their first, fourth or tenth day of illness, which captures the minimum, average, and maximum of time for patients presenting to hospitals seeking care. We simplified the health states for any infection type to four core states: Mild, Severe, Recovered and Death. The exact daily transition probabilities depended upon the specific infection etiology and treatment (i.e., with or without antibiotics). We assumed that all Severe patients were immediately hospitalized and assigned to a diagnostic and treatment strategy if it was the first hospital visit (no prior testing). We also assumed that deaths during the 45-day time horizon were caused only by severe complications of infections.

### Diagnostic and treatment strategies

In the two Thai settings, we evaluated fifteen strategies with different test and treatment options towards febrile disease management (Table 2). Treatment for leptospirosis and scrub typhus was assumed to be a one-week course of antibiotics (e.g., doxycycline) [12, 13]. For dengue and other viral infections, supportive care is standard practice and no antibiotic treatment was assumed. Diagnostic testing included pathogen-specific rapid diagnostics tests (RDT) and Polymerase Chain Reaction (PCR) tests for leptospirosis, typhus, and dengue. A positive bacterial test result or a negative viral test result would lead to the prescription of antibiotics. Although still not widely available in health care settings, we also considered the use of nucleic acid amplification for all three pathogens in a single test (e.g., multiplex PCR) [25, 26]. We assumed TAT was one day and two days for RDT and for PCR, respectively (varied in sensitivity analysis). In both Thailand settings examined, the probability of presenting with leptospirosis was higher than scrub typhus infection. As a consequence (and to limit the total number of possible candidate strategies), we did not include typhus-led testing strategies (i.e., single typhus RDT/PCR). We evaluated additional candidate strategies in S1Text, Section 7.

**Table 2:** Strategies evaluated for two Thai settings using Markov cohort models.

*Strategy*			*Test interpretation treatment decision*
**No tests**	1	No Antibiotics	Reference. No antibiotics for neither mild nor severe patients
	2	Empirical to all	Antibiotics to all patients
	3	Empirical to Severe	Antibiotics to patients in Severe states
***Single Tests***	4	Dengue RDT	Dengue RDT positive: out(in)patient care w/o antibiotics Dengue RDT negative: antibiotic
	5	Dengue PCR	Dengue PCR positive: out(in)patient care w/o antibiotics Dengue PCR negative: antibiotic
	6	Lepto RDT	Lepto RDT positive: antibiotic Lepto RDT negative: out(in)patient care w/o antibiotics
	7	Lepto PCR	Lepto PCR positive: antibiotic Lepto PCR negative: out(in)patient care w/o antibiotics
***Sequential Tests***	8	S: Lepto RDT, typhus RDT	Lepto RDT positive: antibiotic Lepto RDT negative: perform Typhus RDT Typhus RDT positive: antibiotic Typhus RDT negative: out(in)patient care w/o antibiotics
	9	S: Lepto PCR, typhus RDT	Lepto PCR positive: antibiotic Lepto PCR negative: perform Typhus RDT Typhus RDT positive: antibiotic Typhus RDT negative: out(in)patient care w/o antibiotics
	10	S: Lepto RDT, typhus PCR	Lepto RDT positive: antibiotic Lepto RDT negative: perform Typhus PCR Typhus PCR positive: antibiotic Typhus PCR negative: out(in)patient care w/o antibiotics
***Parallel Tests***	11	P: Lepto PCR, typhus PCR	Lepto PCR or Typhus PCR positive: antibiotic Lepto PCR and Typhus PCR negative: out(in)patient care w/o antibiotics
	12	P: Lepto RDT, typhus RDT	Lepto RDT or Typhus RDT positive: antibiotic Lepto RDT and Typhus RDT negative: out(in)patient care w/o antibiotics
	13	P: Lepto PCR, typhus RDT	Lepto PCR or Typhus RDT positive: antibiotic Lepto PCR and Typhus RDT negative: out(in)patient care w/o antibiotics
	14	P: Lepto RDT, typhus PCR	Lepto RDT or Typhus PCR positive: antibiotic Lepto RDT and Typhus PCR negative: out(in)patient care w/o antibiotics
***Multiplex PCR***	15	Multiplex PCR	Lepto or typhus PCR positive: antibiotic Dengue positive: out(in)patient care w/o antibiotics Lepto, typhus, or dengue negative: out(in)patient care w/o antibiotics

Each strategy consists of testing options from no testing to testing using: Rapid tests (RDT), polymerase chain reaction (PCR), and Multiplex PCR for leptospirosis, typhus, and dengue simultaneously, Sequential (S): run tests in sequence; Parallel (P): run tests simultaneously.

We assumed all Mild state patients received outpatient care (with or without antibiotic prescription), where practices such as follow-up of patients, adequate bed rest and fluid intake are common. Patients who progressed to Severe state (i.e., patients demonstrating various clinical signs in severe febrile illness) were hospitalized. The current protocol recommended by the WHO in resource-limited settings is empirical antibiotic treatment to patients presenting with severe illness [12]. For comparison purposes, we also considered an expanded empirical treatment strategy that includes treating both Mild and Severe patients with antibiotics without any diagnostic testing. Lastly, we also considered a “No Antibiotics” strategy to monitor natural disease progression, where no test and no antibiotics were given, but patients in Severe states were hospitalized. A diagram of patient flow through the model can be found in S1 Fig.

### Outcomes measured

Outcome measures included:

1. Costs and health outcomes: direct and indirect healthcare costs incurred and health burden (in DALYs, definition and calculation procedure in S1 Text, Section 3.1.3),
2. Antibiotics underuse and overuse. For a given strategy, we tracked the proportion of patients over- and under-treated with antibiotics (*Prob(over)* and *Prob(under)*).

To account for antibiotic overuse when evaluating the strategies, we first considered the three-dimensional outcome space consisting of DALYs, costs, and *Prob(over)* instead of the standard two-dimensional outcome space consisting of only DALYs and costs. Secondly, for each strategy, we assigned a penalty per unnecessarily prescribed course of antibiotics (*w*_*over*_), and monetized DALYs by assigning a willingness-to-pay (WTP) per DALY averted. DALYs averted, incremental costs and the incremental *Prob(over)* for a given strategy is the difference between the DALYs, costs, and *Prob(over)* incurred by the strategy and those incurred by the “No tests/No Antibiotics” strategy (Strategy 1 in Table 2). In our main analysis, we set the WTP value to be Thailand’s GDP per capita in 2016: $5,907.91 [27]. Then for each strategy, we computed its resulting net monetary benefit (NMB), where

$$NMB=(DALYs\;averted)\times WTP-(incremental\;costs)-w_{over}\;(incremental\;Prob(over)).$$

By considering a range of values for willingness-to-pay per DALY averted, and for penalty per unnecessary course of antibiotics, we quantified the ranges of the NMB of each strategy, and conducted a three-dimensional cost-effectiveness analysis comparing different strategies. We provide discussion on the form of augmented cost-effective analysis in S1 Text, Section 4, and the methodology for finding a three-dimensional effectiveness frontier in S1 Text, Section 5.

### Markov model calibration

The transition probabilities were calibrated to match quantities reported in the medical literature, such as mean duration of fever, and mortality rates with or without antibiotics. Test costs, test sensitivities and specificities, health costs and DALY-related parameters for all possible causes of infection were obtained from the published literature. More detailed explanations, exact values, and references are provided in S1 Text, Section 3.1 and S1 Table.

### Sensitivity analysis

We conducted univariate and probabilistic sensitivity analyses by varying the disease occurrence probabilities, antibiotic effectiveness to lower disease progression from Mild to Severe, delay in patients presenting for care, as well as other parameter values including test sensitivities and specificities, test and treatment costs, health utilities and disease progression parameters.

## Results

### Balancing antibiotic over- and underuse

We denoted by *p*_*bac*_ the probability of presenting with bacterial infection for which a test exists, $p_{{other}_{bac}}$ the probability of presenting with bacterial infection for which no test exists, *p*_*viral*_ the probability of presenting with a viral infection for which a test exists, and *p*_*other*_ the probability of presenting with infections not treatable with the primary antibiotic for which no test exists. We assumed that all test sensitivities and specificities fall between 50% and 100%, and that any test’s sensitivity is less than or equal to any specificity, and the sum of the four disease occurrence probabilities (*p*_*bac*_, $p_{{other}_{bac}}$, *p*_*viral*_ and *p*_*other*_) is 1.

Our metric for evaluation was the total weighted penalty of antibiotic under- and overuse (Eq (1)) in the generic setting with four infection types and three treatment strategies (antibiotic to all patients; bacterial test and if positive administer antibiotic; viral test and if negative administer antibiotic). When comparing two of the strategies at a time, we found the following sufficient conditions that guarantee strategy dominance. Notably, these conditions only involve a minimal number of parameters, and do not depend on test accuracy and illness stage:

- If ${p_{bac}+p}_{{other}_{bac}}\leq \frac{w_{over}}{w_{over}+w_{under}}$, then both the bacterial and the viral test strategies have a smaller overall penalty than the empiric antibiotic strategy. Here, ${p_{bac}+p}_{{other}_{bac}}$ is the overall probability of presenting with bacterial disease, treatable with antibiotics; *w*_*under*_ or *w*_*over*_ is the penalty per patient under- or over- treated with antibiotics, respectively. This condition states that, if the probability of presenting with bacterial disease is low (and therefore the probability of presenting with viral disease or other, non-bacterial disease is high), or the penalty for overuse of antibiotics is large, then using a strategy that tests for either viral or bacterial disease before prescribing antibiotics is preferred to the strategy that distributes antibiotic treatment to all patients.
- If $\frac{w_{under}}{w_{over}}({p_{bac}+p}_{{other}_{bac}})\leq p_{other}$, then the strategy administering the bacterial test incurs a smaller overall penalty than the strategy administering the viral test. This condition states that, when choosing between the viral and bacterial test, if the probability of presenting with an antibiotic-treatable infection is low, or the penalty for over-treating is high, then the bacterial test strategy outperforms the viral test strategy.
- If $\frac{w_{under}}{w_{over}}(p_{{other}_{bac}})>p_{other}+p_{viral}$, then the strategy administering the viral test leads to a smaller overall penalty than the strategy administering the bacterial test. This condition states that, when choosing between the viral and bacterial test, if the probability of presenting with infections non-treatable with antibiotic is low, or the penalty for under-treatment is high, then the viral test strategy outperforms the bacterial test strategy.

### Numerical results: Bacterial- versus viral-endemic settings in Thailand

**Fig 1:**
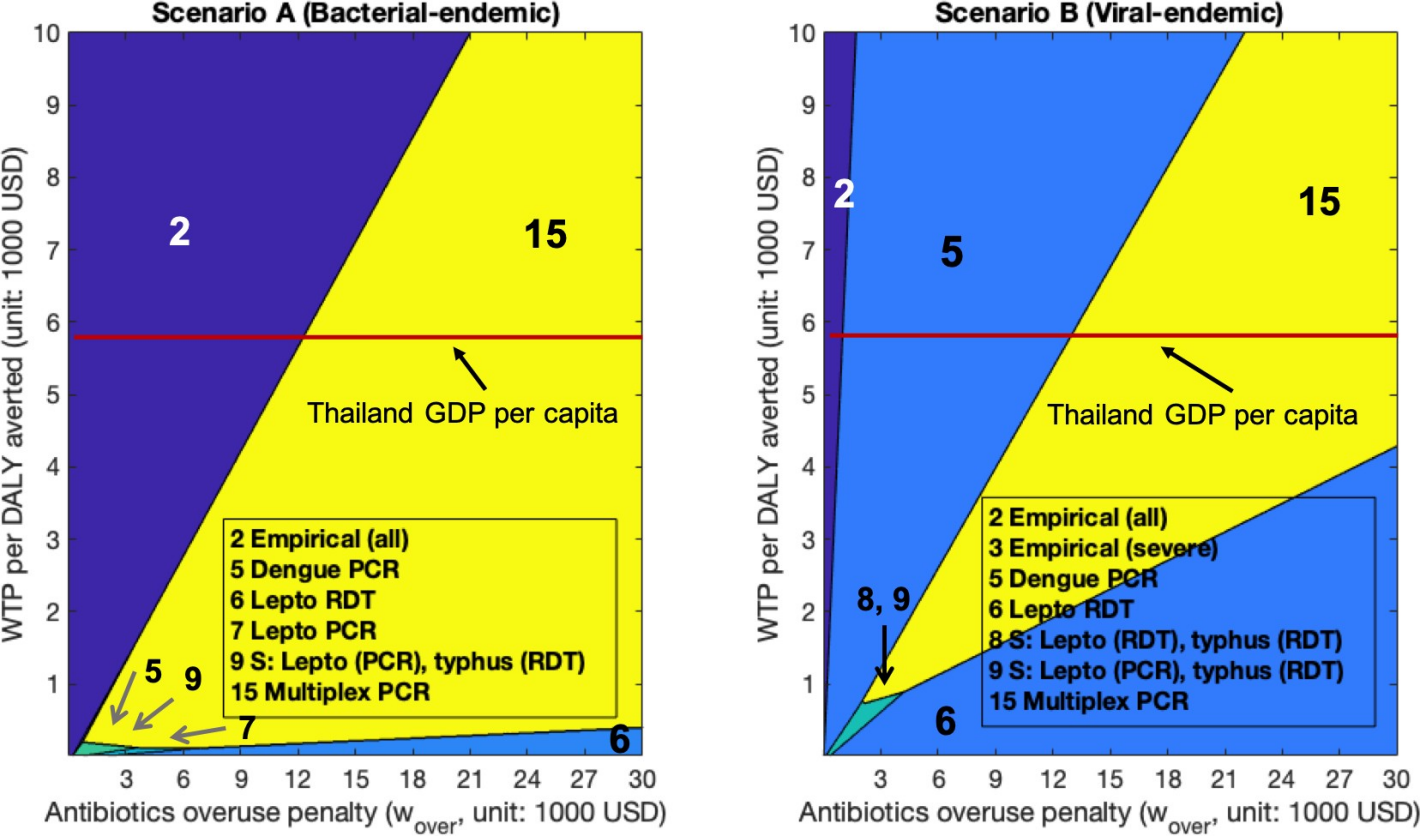
Highest net monetary benefit policies for patients presenting on the first day of illness. We vary willingness-to-pay (WTP) on the y-axis and penalty (w_over_) on the x-axis. (A): Bacterial-endemic Scenario A (B): Viral-endemic Scenario B.

**Fig 2:**
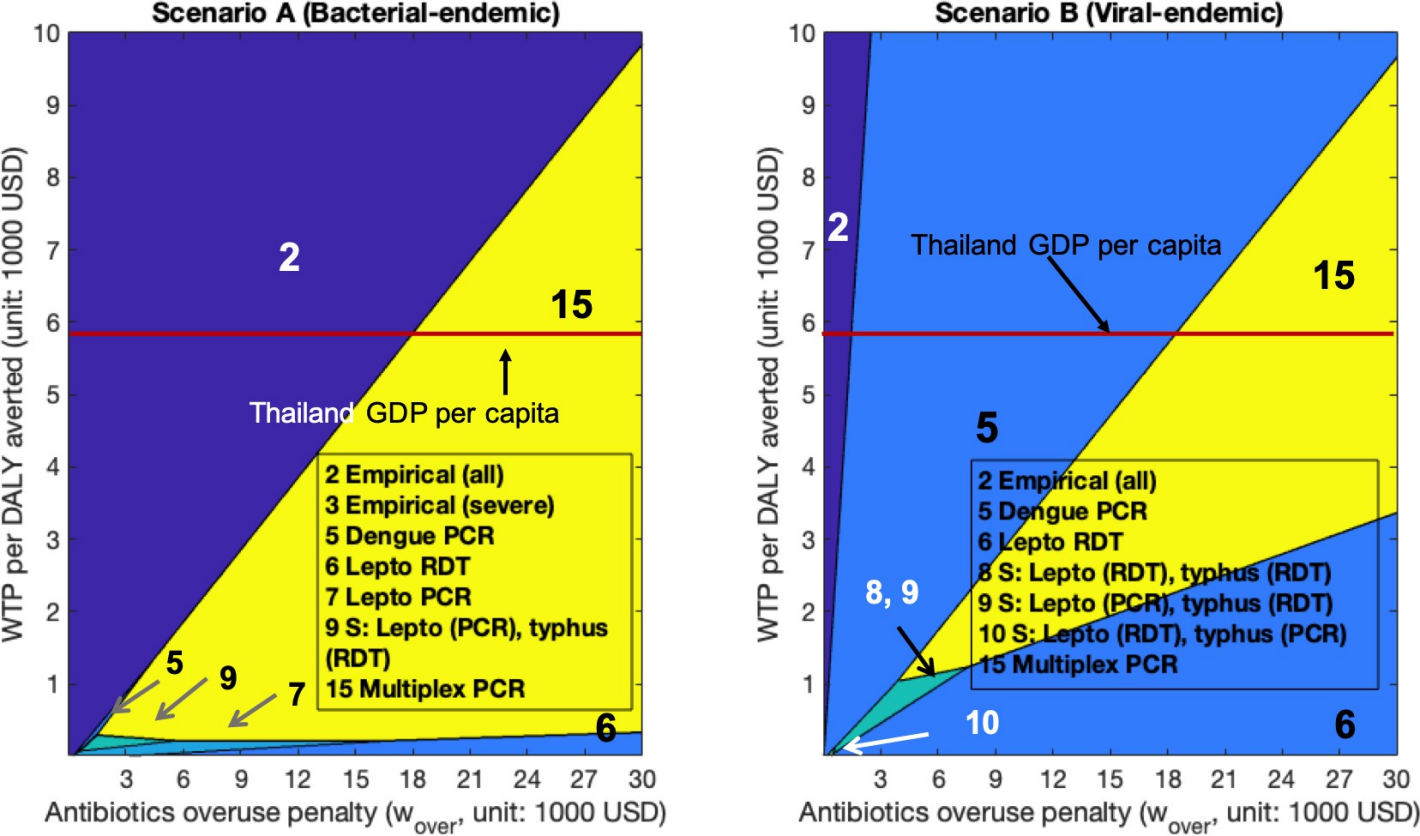
Highest net monetary benefit policies for patients presenting on the fourth day of illness. We vary willingness-to-pay (WTP) on the y-axis and penalty (w_over_) on the x-axis. (A): Bacterial-endemic Scenario A (B): Viral-endemic Scenario B.

**Fig 3:**
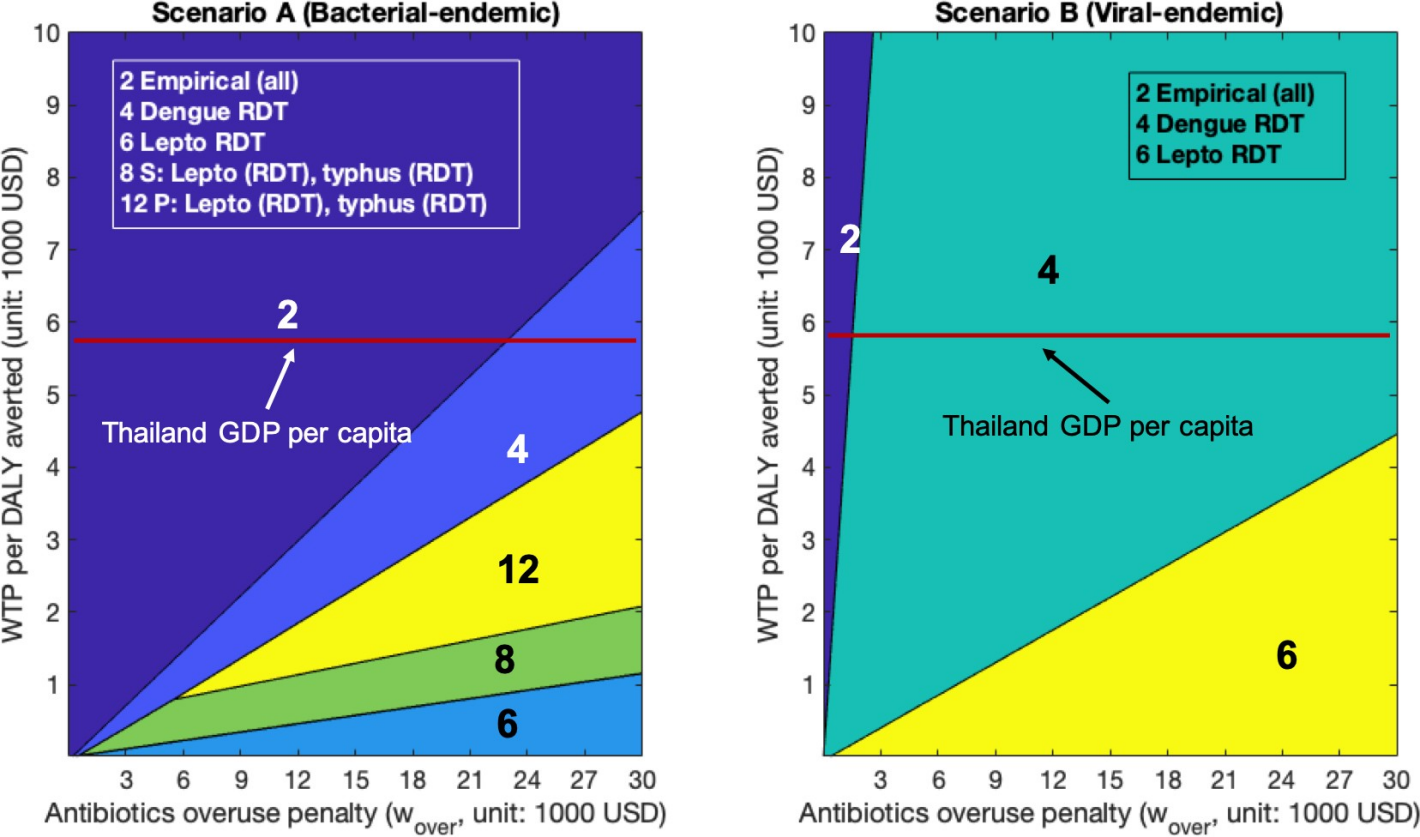
Highest net monetary benefit policies for patients presenting on the tenth day of illness. We vary willingness-to-pay (WTP) on the y-axis and penalty (w_over_) on the x-axis. (A): Bacterial-endemic Scenario A (B): Viral-endemic Scenario B.

We now turn to evaluate health outcomes, costs and antibiotic overuse outcomes in the more realistic bacterial-endemic setting (Scenario A) and viral-endemic setting (Scenario B) in Thailand. The per-patient costs incurred (in USD), health burden (in DALY), and antibiotics overuse (*Prob*(*over*)), underuse (*Prob*(*under*)) for each strategy are shown in Table 3 for patients seeking care on the fourth day of illness (average day at hospital presentation). Results for patients seeking care on the first (min), and tenth (max) day of illness can be found in S3 Table and S4 Table. Figs 1–3 show the optimal policy (highest NMB) at varying values of WTP and antibiotic overuse penalty (*w*_*over*_) for patients presenting on day one of illness (Fig 1), day four of illness (Fig 2) and day ten of illness (Fig 3).

**Table 3:** Per-patient costs (USD), DALYs incurred, antibiotic overuse (*Prob(over)*) and underuse (*Prob(under)*) for febrile patients seeking care on the fourth day (average day) of illness and undergoing various test and treat strategies.

Strategies		*Scenario A*: *Bacterial-Endemic*				*Scenario B*: *Viral-Endemic*			
		*Cost*	*DALY*	*P(over)*	*P(under)*	*Cost*	*DALY*	*P(over)*	*P(under)*
*1*	No Antibiotics *º	216.166	2.911	0	0.394	138.978	1.258	0	0.109
*2*	Empirical All *º	100.576	0.964	0.193	0	108.692	0.721	0.421	0
*3*	Empirical Severe *	137.058	1.440	0.055	0.248	118.573	0.851	0.119	0.068
*4*	Dengue RDT	137.329	1.546	0.102	0.085	119.048	0.881	0.144	0.023
*5*	Dengue PCR *º	122.947	1.254	0.102	0	116.484	0.801	0.095	0
*6*	Lepto RDT *º	160.500	1.940	0.003	0.163	133.291	1.127	0.007	0.072
*7*	Lepto PCR *	148.763	1.696	0.007	0.086	133.176	1.089	0.015	0.056
*8*	S: Lepto RDT, typhus RDT º	155.767	1.859	0.006	0.280	131.570	1.097	0.012	0.125
*9*	S: Lepto PCR, typhus RDT *º	152.117	1.627	0.009	0.143	138.125	1.063	0.019	0.096
*10*	S: Lepto RDT, typhus PCR º	152.708	1.844	0.008	0.254	129.575	1.092	0.016	0.113
*11*	P: Lepto PCR, typhus PCR	165.897	1.581	0.014	0.063	154.246	1.040	0.030	0.046
*12*	P: Lepto RDT, typhus RDT	163.842	1.838	0.007	0.143	140.368	1.089	0.014	0.064
*13*	P: Lepto PCR, typhus RDT	159.399	1.612	0.010	0.069	146.815	1.055	0.021	0.049
*14*	P: Lepto RDT, typhus PCR	175.228	1.881	0.010	0.121	148.564	1.085	0.021	0.055
*15*	Multiplex PCR*º	184.539	1.524	0.007	0.052	176.346	1.042	0.015	0.047

For each scenario, we identified the strategies that were on a three-dimensional effectiveness frontier made of DALYs, costs and antibiotic overuse. DALY = disability-adjusted life years

* = strategies on the effectiveness frontier (economically efficient) for Scenario A (bacterial-endemic)

º = strategies on the effectiveness frontier (economically efficient) for Scenario B (viral-endemic).

As shown in Table 3, Figs 1–3, S3 Table, and S4 Table, under a standard analysis that considers only the dimensions of health benefit (DALYs averted) and incremental costs, without penalizing antibiotic overuse, empirical antibiotic treatment to all patients (Strategy 2) always dominated all other strategies, regardless of when patients presented for care or which endemic scenario we considered. When patients sought care on day four of illness (Table 3), empirical antibiotic treatment incurred the lowest cost: $100.6/patient in bacterial-endemic Scenario A and $108.7/patient in viral-endemic Scenario B. This strategy also incurred the lowest DALYs of 0.9 DALYs/patient in Scenario A and 0.7 DALYs/ patient in Scenario B. If patients delayed in presenting for care to day ten of illness (S4 Table), both costs and DALYs increased (compared to day four), but still remained the lowest for Strategy 2 at $149.5/patient (Scenario A), $122.1/ patient (Scenario B) and 1.7 DALYs/patient in Scenario A, and 0.9 DALYs/patient in Scenario B. For patients presenting to the hospital on the first day of illness (S3 Table), both costs and DALYs decreased compared to day four: for Strategy 2, costs were $47.5/patient (Scenario A) and $94.1/ patient (Scenario B) and 0.2 DALYs/patient in Scenario A, and 0.5 DALYs/patient in Scenario B.

However, Strategy 2 resulted in the highest likelihood of antibiotic overuse per patient (Scenario A: 38.1%, 19.3%, 7.5% for patients presenting on day one, day four, and day ten respectively; Scenario B: 82.9%, 42.1%, 16.3% for day one, day four, and day ten respectively) (Table 3, S3 Table, S4 Table). In Scenario A, Strategy 2 became suboptimal with respect to NMB (at WTP = Thailand’s GDP per capita) when the antibiotic overuse penalty was larger than $12,800/course, $18,400/course, $23,900/course for patients presenting on day one, day four, and day ten, respectively (Figs 1–3). In the viral-endemic Scenario B, Strategy 2 became suboptimal when the antibiotic overuse penalty was larger than $1,100/course, $1,500/course, $1,600/course for patients presenting on day one, day four, and day ten, respectively (Figs 1–3). When setting the penalty (*w*_*over*_) to be equal to the WTP (Thailand’s GDP per capita), we found that empirical antibiotic treatment (Strategy 2) was only optimal in Scenario A (not in Scenario B), which is consistent with the analytical results (detailed estimation procedure in S1 Text, Section 3.2).

Strategy 3 (empirical antibiotic treatment to patient in Severe states only) was optimal in two settings and had very small optimal regions at low WTP and low penalty. In Scenario A on day four (Fig 2A), Strategy 3 was optimal for a 1 to 3 ratio of WTP to penalty, from $0 to $300 WTP. In Scenario B on day one (Fig 1B), Strategy 3 was optimal for WTP from $0 to $20 and penalty from $120/course to $130/course.

Compared to Scenario A, we observed that single test strategies (Dengue PCR or Dengue RDT, and/or Lepto RDT) had the highest NMB for a much wider range of WTP and penalty values in Scenario B than in Scenario A. For example, when we fixed the WTP to Thailand’s GDP per capita, and presentation day as the first day of illness (Fig 1), Dengue PCR was optimal in terms of NMB when the overuse penalty was about $13,000/course in Scenario A. On the other hand, Dengue PCR was optimal for penalty between $1,100/course to $13,000/course in Scenario B. These findings were consistent with our analytical results when balancing antibiotics overuse and underuse: testing strategies are more likely to be optimal when the probability of presenting with AT disease is smaller. Lastly, we observe from Fig 1 that Multiplex PCR test (Strategy 15), with sensitivities ≥ 90% for each disease component, dominated other strategies for penalty ≥ $13,000/course in both settings when the WTP per DALY averted is set to Thailand’s GDP per capita.

Policies with highest NMB for patients presenting on the fourth day of illness (Fig 2) are similar to those for patients presenting on the first day of illness (Fig 1). Lastly, RDT-led strategies had higher NMB when patients presented on the tenth day of illness (Fig 3). This is because test sensitivities of RDT tests are higher than those of PCR tests when patients present to hospitals late (S1 Table).

The flexibility of the model allows us to consider multiple additional strategies. For example, in settings where viral disease is more prevalent, healthcare decision-makers and administrators might consider sequential test strategies that include viral disease tests. We described in supplementary material (S1 Text Section 7, S8 Table and S9 Table) four additional sequential strategies starting with a dengue test, and if negative, a test for leptospirosis. The dengue-led strategies were optimal with respect to NMB for WTP values between $0 and $250 in Scenario A, and between $0 and $4,000 in Scenario B (S13– S15 Figs).

### Sensitivity analysis

The general structure of the optimal strategy sequence when varying antibiotic overuse penalty while setting the WTP to Thailand’s GDP per capita did not change when considering: 1) two additional disease probability distribution scenarios in Thailand (S1 Text, Section 6.1); and 2) reduced antibiotic effectiveness in preventing Mild patients progressing to Severe states (S1 Text, Section 6.2). In all cases (including the base-case) considered, RDT-led strategies became optimal in terms of NMB when patients presented on the tenth day of illness. We also observed that patients who presented early incurred better health outcomes.

Under additional disease probability distributions, test strategies were optimal at lower probability of bacterial disease and higher penalty (*w*_*over*_). In the average day-of-presentation (day four) scenario and the high bacterial disease prevalence setting (Set 1 in S5 Table), Strategy 15 (Multiplex PCR test) had the highest NMB for penalties greater than $20,000/course (S3 Fig). In the low bacterial disease prevalence setting (Set 2 in S5 Table), Strategy 15 (Multiplex PCR test) had the highest NMB for penalties greater than $8,500/course (S4 Fig).

Reduced antibiotic effectiveness in preventing progression to Severe states makes Strategy 2 (empirical antibiotic treatment to all patients) less preferable: For example, in Scenario A, when fixing the WTP to Thailand’s GDP per capita and assuming patients present on day four (S8 Fig), Strategy 2 had the highest NMB for penalties less than $18,000/course, $16,000/course, and $12,000/course when antibiotic effectiveness was assumed to be 100%, 75%, and 50% respectively.

We performed one-way sensitivity analysis for all variables while fixing the disease occurrence probabilities to the ones in Scenario A, and assumed that patients presented on the average day of presentation (day four) to hospitals (S1 Text, Section 6.3). We found that empirical antibiotic treatment to all patients had the highest NMB at younger patient age, lower leptospirosis-test sensitivity, higher disease-specific mortality rate, and longer TAT (S2 Table, S5 Fig). Varying remaining parameters, such as test, health costs and disability weights, did not change our conclusions.

Lastly, we conducted a probabilistic sensitivity analysis (PSA) by varying all model parameters simultaneously using Monte Carlo simulation (S1 Text, Section 6.4, and S6 Fig). Our analyses indicated that when the penalty on antibiotics overuse was between $0 to $10,000/course, empirical treatment to all patients was most likely optimal, while strategies with single dengue tests (Strategy 5 Dengue PCR and Strategy 4 Dengue RDT) could be alternatives to reduce antibiotics overuse. Another critical observation was that PCR-led strategies (Strategy 15, Strategy 11) had a much higher chance of being optimal (40% probability for Strategy 15, 25% probability for Strategy 11) than RDT-led strategies even when delay in seeking care (0–10 days) and TAT (0–3 days) were randomly sampled, suggesting that PCR-led strategies were robust under the sampled parameter distributions.

## Discussion

Febrile illness causing diseases is a significant public health burden worldwide [8, 28, 29]. Because of barriers to accessing proper diagnostic tests, empirical antibiotic administration is often recommended, in particular for severe illness [12]. Furthermore, previous cost-effectiveness studies have shown that empirical administration of antibiotic therapy is the optimal strategy if antibiotic overuse is not a concern [12, 13]. However, antibiotic overuse has indeed become a significant health threat globally, as it is a key driver for antibiotic resistance [30–32].

In this study, we first proposed a simple framework to evaluate the effectiveness of different strategies for diagnosis and treatment by balancing antibiotic overuse and underuse. Our framework produced three highly generalizable sufficient conditions that ensured pairwise strategy dominance in terms of a weighted overall penalty of antibiotics under- and overtreatment. We then built detailed Markov models of disease progression in order to capture relevant, practical impacts, such as test costs and turnaround times and delays in seeking care. In both the simple framework and a more realistic setting where more strategies were available, we found that empirical antibiotic treatment to all patients incurred the least DALYs and healthcare costs when the reduction of unnecessary antibiotic use was not a concern. This is because, for patients with a bacterial disease, early antibiotic treatment decreased illness duration in Mild state patients and effectively prevented patients in Mild state from progressing to Severe state which accrued significantly higher DALYs (S1 Table).

Considering the limitations in diagnostic laboratory access, empirical treatment is often recommended to patients suspected of a specific bacterial illness, in particular if severe [12, 33]. In our model, with the baseline assumption that antibiotic treatment was 100% effective in preventing progression from Mild to Severe, empirical antibiotic administration to solely patients with severe clinical signs was optimal only in a few scenarios with very low WTP and low penalty (Fig 1B and Fig 2A). When examining a more realistic setting in which early antibiotic treatment was not 100% effective in preventing progression from Mild to Severe stage, empirical antibiotics to only Severe patients became optimal in the high-penalty, high-WTP quadrant. For example, when setting the WTP to Thailand’s GDP per capita, empirical treatment only to Severe patients was optimal for penalty values from $12,000 to $27,000/course when assuming 50% antibiotic effectiveness and the average day of presentation in bacterial-endemic Scenario A (S8 Fig). This is because using an antibiotic with reduced ability to prevent progression from Mild to Severe state diminished the health benefit of early antibiotic treatment, and thus the relative importance of antibiotic underuse among patients in early (Mild) stage of illness decreased. In such circumstances, prescribing antibiotics to only Severe patients resulted in a balance between antibiotic underuse (i.e., missed antibiotics to AT patients only in the Mild stage) and overuse (i.e., unnecessary antibiotics to Severe NAT patients). Alternatively, when antibiotic overuse reduction was prioritized, high specificity bacterial tests such as Multiplex PCR test or leptospirosis RDT test became optimal (Figs 1–3, S3 Fig, S4 Fig) because many unnecessary antibiotic treatments were prevented by correctly identifying patients without the targeted bacterial disease.

Our three-dimensional cost-effectiveness analysis with case studies in Thailand showed that disease occurrence probabilities, test sensitivity and specificity, and patient illness duration at the time of seeking care are highly influential to the choice of an optimal strategy. Empirical antibiotic administration was less preferred in settings where the probability of viral infections was higher than that of bacterial infections since antibiotics were not effective for viral infections. Among all strategies considered in Table 2, dengue PCR test when presenting on day one or day four of illness, dengue RDT (day ten), and leptospirosis RDT (S1 Table) were optimal for a broader range of penalty and WTP values in viral-endemic Scenario B than in bacterial-endemic Scenario A (Figs 1–3). In most strategies we considered, a positive bacterial test result or a negative viral test result would lead to the prescription of antibiotics. Therefore, as NAT infections became predominant, viral tests with high sensitivities became optimal by preventing many NAT patients from antibiotic treatment initiation. Similarly, bacterial tests with high specificities could correctly identify those patients who did not require antibiotic therapy, reducing unnecessary antibiotic administration to NAT patients.

Increasing patients’ delay in seeking care from 1 day to 10 days increased costs threefold, and DALYs sevenfold (S3 Table, S4 Table). This is consistent with recommendations for early diagnosis and treatment in order to avoid worsened health outcomes [34, 35]. For patients referred to hospitals on their first or fourth day of illness, we found that PCR-led strategies were more likely to be on the effectiveness frontier compared to RDT-led strategies (Figs 1 and 2, S3 Fig, S4 Fig) since PCR tests had higher accuracy in the early stage of illness where RDTs were more accurate in the later stage (S1 Table). Therefore, if patients presented to hospitals on their fifth day of illness or later, RDT-led strategies outperformed PCR-led strategies (Fig 3, S3 Fig, S4 Fig). Consequently, a proper understanding of diagnostic test performance by patient stage of illness is critical to inform the right choice of diagnostic tests.

When all model parameters were varied simultaneously in PSA, PCR-led strategies were more likely to be optimal than RDT-led strategies (details in S1 Text, Section 6.4). We sampled patient illness duration at the time of seeking care as a triangle distribution from zero up to ten days with an average of four days. As a consequence, more instances were sampled where patients presented to hospitals with less than five days of illness. Given this distribution, more health benefits were accrued by prescribing PCR tests, which were more accurate in the early stage of illness (S1 Table). These results highlight the importance of accurately estimating not just the mean, but also the variation in patient presentation delays.

Lastly, our analysis also showed that the choice of optimal strategy was sensitive to patient age, the amount of resources available, TATs and the level of importance of antibiotic use reduction in the respective settings. Such conditions led to increased importance of antibiotic underuse. Specifically, younger patient age resulted in higher DALYs in case of death, more resources available led to higher penalty per DALY incurred, longer TATs resulted in less value of information gained from performing tests, and if antibiotic overuse reduction was not a concern, then ensuring that AT patients receive antibiotic treatment was prioritized. Our modeling results showed that empirical antibiotic administration to all patients outperformed testing strategies in terms of NMB (S3 Fig, S4 Fig, S5 Fig, S2 Table) under either of these conditions.

Our analysis has several limitations. First, we assumed that the underlying disease probabilities are known to policymakers. In practice, it might be difficult for health administrators to know the actual underlying probabilities of a competing source of infections. However, we considered scenarios that varied these probabilities from bacterial-endemic to viral-endemic in order to evaluate the importance of accurately estimating these probabilities. Improved surveillance and knowledge of local conditions are generally needed to inform clinical decision-making. Second, we did not model the dynamic transmission of diseases, as the focus of this present study was to address the key questions of what diagnostic test to use and when to administer antibiotics to already infected patients. Nevertheless, the optimal strategy structure remains the same through various sensitivity analyses performed. Thirdly, to limit the numbers of candidate strategies, we considered single viral test settings and reported a few additional multiple test strategies in the supplementary material (sequential testing with viral test components). Nevertheless, our model framework is capable of evaluating any potential strategies. Lastly, quantifying the effects of antibiotics overuse is difficult due to the long-term nature of such effects (i.e., not captured within the 45-day time horizon). To mitigate the difficulty in quantifying the consequences of antibiotics overuse, in our analysis, we provided a wide range of monetary penalties per course of antibiotics unnecessarily prescribed, which resulted in a sequence of optimal policies.

Laboratory disease diagnosis plays a key role in the provision of care, but it requires investments in financial and human resources. There are an increasing number of tests at the disposal of hospitals and providers, making the decision of which test to use non-trivial. Clinical trials have been the gold standard in evaluating candidate strategies, but oftentimes such trials cannot be carried out due to ethical, logistical or cost reasons [33]. Mathematical models are especially useful in such cases [36], including the present problem of differentiating febrile illnesses in order to inform antibiotic treatment. The mathematical modeling approach presented here can be used to inform the potential gains from making specific diagnostic tests available and their optimal use, while taking into consideration a full spectrum of parameters and settings. This study expanded on previous work on modeling studies of undifferentiated febrile illness [12, 13, 18–20] to incorporate additional features such as multiple competing infection etiologies, patients’ delays in seeking care and the illness-stage-dependent accuracy of diagnostic tests. To the best of our knowledge, this is the first study to propose a decision-making framework balancing antibiotic overuse and underuse to optimize the choice of treatment and diagnosis strategy. Our analysis highlights the importance of diagnostic tests in helping to reduce antibiotic usage in the clinical management of febrile illnesses, while pointing out that the choice of strategy depends on the disease distribution and diagnostic test parameters.

## Supporting information

S1 CHEERS Checklist Items to include when reporting economic evaluations of health interventions. (DOCX)

S1 Text TECHNICAL APPENDIX. (DOCX)

S1 Table Values for model variables. (DOCX)

S2 Table Results of one-way sensitivity analysis (numbers are the corresponding strategy orders). We fixed the disease occurrence probabilities to the ones in Scenario A, and set the WTP to Thailand GDP per capita. We assumed that patients presented to hospitals on day four (average day of illness). Strategy 2: Empirical to all; Strategy 5: Dengue PCR; Strategy 9: S: Lepto PCR, typhus RDT; Strategy 13: P: Lepto PCR, typhus RDT; Strategy 15: Multiplex PCR.
(DOCX)

S3 Table Strategy outcomes: per-patient costs and disability-adjusted life years (DALYs) incurred, antibiotic overuse (*Prob(over*)) and underuse (*Prob(under))* and for patients seeking care on the first day of illness. For each scenario, we identified the strategies that were on the three-dimensional effectiveness frontier, where the three dimensions are DALY, cost and antibiotic overuse. * = strategies on the effectiveness frontier (economically efficient) for Scenario A (bacterial-endemic); º = strategies on the effectiveness frontier (economically efficient) for Scenario B (viral-endemic).
(DOCX)

S4 Table Strategy outcomes: per-patient costs and disability-adjusted life years (DALYs) incurred, antibiotic overuse (*Prob(over)*) and underuse (*Prob(under)*) and for patients seeking care on the tenth day of illness. For each scenario, we identified the strategies that were on the three-dimensional effectiveness frontier, where the three dimensions are DALY, cost and antibiotic overuse. * = strategies on the effectiveness frontier (economically efficient) for Scenario A (bacterial-endemic); º = strategies on the effectiveness frontier (economically efficient) for Scenario B (viral-endemic).
(DOCX)

S5 Table Sensitivity analysis of disease occurrence probabilities. Values obtained from [6]
(DOCX)

S6 Table Per-patient costs (USD), DALYs incurred, antibiotic overuse (*Prob(over)*) and underuse (*Prob(under)*) for febrile patients seeking care on the fourth day (average day) of illness and undergoing various test and treat strategies, with 75% antibiotic effectiveness. * = strategies on the effectiveness frontier (economically efficient) for Scenario A (bacterial-endemic); º = strategies on the effectiveness frontier (economically efficient) for Scenario B (viral-endemic).
(DOCX)

S7 Table Per-patient costs (USD), DALYs incurred, antibiotic overuse (*Prob(over)*) and underuse (*Prob(under)*) for febrile patients seeking care on the fourth day (average day) of illness and undergoing various test and treat strategies, with 50% antibiotic effectiveness. * = strategies on the effectiveness frontier (economically efficient) for Scenario A (bacterial-endemic); º = strategies on the effectiveness frontier (economically efficient) for Scenario B (viral-endemic).
(DOCX)

S8 Table Additional Strategies evaluated for two Thai settings using Markov cohort models. (DOCX)

S9 Table Per-patient costs (USD), DALYs incurred, antibiotic overuse (*Prob(over)*) and underuse (*Prob(under)*) for febrile patients on different days of presentation and undergoing various test and treat strategies. * = strategies on the effectiveness frontier (economically efficient) for Scenario A (bacterial-endemic); º = strategies on the effectiveness frontier (economically efficient) for Scenario B (viral-endemic).
(DOCX)

S1 Fig Patient Flow Diagram. (TIFF)

S2 Fig Sample Markov trace of disease progression for leptospirosis. Patients enter the model in Mild state, during the 45-day horizon, they could progress to Severe state, become recovered, or dead.
(TIF)

S3 Fig Policies with highest NMB with by varying WTP (y-axis) and w_over_ (x-axis). We fixed disease occurrence probability vector as Set 1 in S5 Table. NMB = net monetary benefit, WTP = willingness-to-pay, w_over_ = antibiotic overuse penalty.
(TIF)

S4 Fig Policies with highest NMB with by varying WTP (y-axis) and w_over_ (x-axis). We fixed disease occurrence probability as Set 2 in S5 Table. NMB = net monetary benefit, WTP = willingness-to-pay, w_over_ = antibiotic overuse penalty.
(TIF)

S5 Fig Tornado diagram with variation in selected model parameters. One-way sensitivity analysis, each row (bar) displays the range of Augmented ICER between the empirical antibiotic to all strategy and the Multiplex PCR strategy (patients present to a hospital on day one). We only displayed leptospirosis-specific disease parameters, but all other disease categories share the same structure in the range of ICER change. Augmented ICER = augmented incremental cost-effectiveness ratio, calculated by the ratio of DALY difference and augmented cost difference. Augmented cost = cost + penalty * w_over_; DALY = disability-adjusted life year; PCR = Polymerase Chain Reaction tests.
(TIFF)

S6 Fig Results of probabilistic sensitivity analysis. We fixed WTP = Thailand GDP per capita. The optimal strategy for a given penalty, is the strategy with the highest NMB value.
(TIF)

S7 Fig Highest net monetary benefit policies for patients presenting on the first day of illness in bacterial-endemic Scenario A. We vary willingness-to-pay (WTP) on the y-axis and penalty (w_over_) on the x-axis. (A): 100% antibiotic effectiveness (B): 75% antibiotic effectiveness (C): 50% antibiotic effectiveness.
(TIF)

S8 Fig Highest net monetary benefit policies for patients presenting on the fourth day of illness in bacterial-endemic Scenario A. We vary willingness-to-pay (WTP) on the y-axis and penalty (w_over_) on the x-axis. (A): 100% antibiotic effectiveness (B): 75% antibiotic effectiveness (C): 50% antibiotic effectiveness.
(TIF)

S9 Fig Highest net monetary benefit policies for patients presenting on the tenth day of illness in bacterial-endemic Scenario A. We vary willingness-to-pay (WTP) on the y-axis and penalty (w_over_) on the x-axis. (A): 100% antibiotic effectiveness (B): 75% antibiotic effectiveness (C): 50% antibiotic effectiveness.
(TIF)

S10 Fig Highest net monetary benefit policies for patients presenting on the first day of illness in viral-endemic Scenario B. We vary willingness-to-pay (WTP) on the y-axis and penalty (w_over_) on the x-axis. (A): 100% antibiotic effectiveness (B): 75% antibiotic effectiveness (C): 50% antibiotic effectiveness.
(TIF)

S11 Fig Highest net monetary benefit policies for patients presenting on the fourth day of illness in viral-endemic Scenario B. We vary willingness-to-pay (WTP) on the y-axis and penalty (w_over_) on the x-axis. (A): 100% antibiotic effectiveness (B): 75% antibiotic effectiveness (C): 50% antibiotic effectiveness.
(TIF)

S12 Fig Highest net monetary benefit policies for patients presenting on the tenth day of illness in viral-endemic Scenario B. We vary willingness-to-pay (WTP) on the y-axis and penalty (w_over_) on the x-axis. (A): 100% antibiotic effectiveness (B): 75% antibiotic effectiveness (C): 50% antibiotic effectiveness.
(TIF)

S13 Fig Highest net monetary benefit policies for patients presenting on the first day of illness. We vary willingness-to-pay (WTP) on the y-axis and penalty (w_over_) on the x-axis. (A): Bacterial-endemic Scenario A (B): Viral-endemic Scenario B.
(TIF)

S14 Fig Highest net monetary benefit policies for patients presenting on the fourth day of illness. We vary willingness-to-pay (WTP) on the y-axis and penalty (w_over_) on the x-axis. (A): Bacterial-endemic Scenario A (B): Viral-endemic Scenario B.
(TIF)

S15 Fig Highest net monetary benefit policies for patients presenting on the tenth day of illness. We vary willingness-to-pay (WTP) on the y-axis and penalty (w_over_) on the x-axis. (A): Bacterial-endemic Scenario A (B): Viral-endemic Scenario B.
(TIF)

## Abbreviations

AT: antibiotic-treatable
DALY: disability-adjusted life year
NAT: non-antibiotic-treatable
PCR: polymerase chain reaction (test)
PSA: probabilistic sensitivity analysis
RDT: rapid diagnostic test
TAT: test turnaround times
WHO: World Health Organization
WTP: willingness-to-pay
