# Supplementary material for: When and what to test for: A cost-effectiveness analysis of febrile illness test-and-treat strategies in the era of responsible antibiotic use
Source: PLoS One. 2020 Jan 8;15(1):e0227409. doi: 10.1371/journal.pone.0227409 (PMC6948826; doi:10.1371/journal.pone.0227409)
Supplement: S1 CHEERS Checklist — (DOCX) [file pone.0227409.s001.docx]

**CHEERS Checklist**

**Items to include when reporting economic evaluations of health interventions**

The **ISPOR CHEERS Task Force Report**, *Consolidated Health Economic Evaluation Reporting Standards (CHEERS)—Explanation and Elaboration: A Report of the ISPOR Health Economic Evaluations Publication Guidelines Good Reporting Practices Task Force*, provides examples and further discussion of the 24-item CHEERS Checklist and the CHEERS Statement. It may be accessed via the *Value in Health* or via the ISPOR Health Economic Evaluation Publication Guidelines – CHEERS: Good Reporting Practices webpage: <http://www.ispor.org/TaskForces/EconomicPubGuidelines.asp>

| Section | Item No | Recommendation | Reported on page No/line No |
| --- | --- | --- | --- |
| Title and Abstract | | | |
| Title | 1 | Identify the study as an economic evaluation or use more  specific terms such as “cost-effectiveness analysis”, and  describe the interventions compared. | Title |
| Abstract | 2 | Provide a structured summary of objectives, perspective,  setting, methods (including study design and inputs), results  (including base case and uncertainty analyses), and  conclusions. | Abstract |
| Introduction | | | |
| Background and objectives | 3 | Provide an explicit statement of the broader context for the  study.  Present the study question and its relevance for health policy or practice decisions. | Introduction |
| Methods | | | |
| Target population and subgroups | 4 | Describe characteristics of the base case population and  subgroups analysed, including why they were chosen. | Methods: The Disease Progression Model |
| Setting and location | 5 | State relevant aspects of the system(s) in which the decision(s) need(s) to be made. | Introduction; Methods: Viral-versus bacterial-endemic settings: a cases study in Thailand |
| Study perspective | 6 | Describe the perspective of the study and relate this to the  costs being evaluated. | Methods: Outcomes Measured; S1 Text, Section 3 |
| Comparators | 7 | Describe the interventions or strategies being compared and  state why they were chosen. | Methods: Diagnostic and Treatment Strategies; Table 2 |
| Time horizon | 8 | State the time horizon(s) over which costs and consequences are being evaluated and say why appropriate. | Methods:  The Disease Progression Model; S1 Text, Section 3 |
| Discount rate | 9 | Report the choice of discount rate(s) used for costs and  outcomes and say why appropriate. | Not Applicable |
| Choice of health outcomes | 10 | Describe what outcomes were used as the measure(s) of  benefit in the evaluation and their relevance for the type of  analysis performed. | Methods: Outcomes Measured; S1 Text, Section 3 |
| Measurement of effectiveness | 11a | *Single study-based estimates*: Describe fully the design  features of the single effectiveness study and why the single  study was a sufficient source of clinical effectiveness data. | Not Applicable |
|  | 11b | *Synthesis-based estimates*: Describe fully the methods used for identification of included studies and synthesis of clinical effectiveness data. | Methods: Markov Model Calibration; S1 Text, Section 3.1 |
| Measurement and valuation of preference based outcomes | 12 | If applicable, describe the population and methods used to  elicit preferences for outcomes. | DALY-related, transition-probability-related data/references in S1 Table |
| Estimating resources and costs | 13a | *Single study-based economic evaluation*: Describe approaches used to estimate resource use associated with the alternative interventions. Describe primary or secondary research methods for valuing each resource item in terms of its unit cost. Describe any adjustments made to approximate to opportunity costs. | Not Applicable |
|  | 13b | *Model-based economic evaluation*: Describe approaches and data sources used to estimate resource use associated with model health states. Describe primary or secondary research methods for valuing each resource item in terms of its unit cost. Describe any adjustments made to approximate to opportunity costs. | Cost-related data/references in S1 Table |
| Currency, price date, and conversion | 14 | Report the dates of the estimated resource quantities and unit costs. Describe methods for adjusting estimated unit costs to the year of reported costs if necessary. Describe methods for converting costs into a common currency base and the exchange rate. | S1 Text, Section 3 |
| Choice of model | 15 | Describe and give reasons for the specific type of decision-analytical model used. Providing a figure to show model  structure is strongly recommended. | Methods: The Disease Progression Model; S1 Text Section 3 (page 8); S1 Fig |
| Assumptions | 16 | Describe all structural or other assumptions underpinning the decision-analytical model. | Methods: The Disease Progression Model; S1 Text Section 3 (page 8) |
| Analytical methods | 17 | Describe all analytical methods supporting the evaluation. This could include methods for dealing with skewed, missing, or censored data; extrapolation methods; methods for pooling data; approaches to validate or make adjustments (such as half cycle corrections) to a model; and methods for handling population heterogeneity and uncertainty. | Methods: Markov Model Calibration; Sensitivity Analysis; S1 Text Section 6 |
| Results | | | |
| Study parameters | 18 | Report the values, ranges, references, and, if used, probability distributions for all parameters. Report reasons or sources for distributions used to represent uncertainty where appropriate. Providing a table to show the input values is strongly recommended. | S1 Table |
| Incremental costs and outcomes | 19 | For each intervention, report mean values for the main  categories of estimated costs and outcomes of interest, as well as mean differences between the comparator groups. If  applicable, report incremental cost-effectiveness ratios. | Table 1, and Tables in S3 Table, S4 Table |

| Characterizing uncertainty | 20a | *Single study-based economic evaluation*: Describe the effects of sampling uncertainty for the estimated incremental cost and incremental effectiveness parameters, together with the impact of methodological assumptions (such as discount rate, study perspective). | Not Applicable |
| --- | --- | --- | --- |
|  | 20b | *Model-based economic evaluation*: Describe the effects on the results of uncertainty for all input parameters, and uncertainty related to the structure of the model and assumptions. | Results: Sensitivity Analysis |
| Characterizing heterogeneity | 21 | If applicable, report differences in costs, outcomes, or cost-effectiveness that can be explained by variations between subgroups of patients with different baseline characteristics or other observed variability in effects that are not reducible by more information. |  |
| Discussion | | | |
| Study findings, limitations, generalizability, and current knowledge | 22 | Summarise key study findings and describe how they support the conclusions reached. Discuss limitations and the  generalisability of the findings and how the findings fit with  current knowledge. | Discussion: paragraphs 2- 6 |
| Other | | | |
| Source of funding | 23 | Describe how the study was funded and the role of the funder in the identification, design, conduct, and reporting of the analysis. Describe other non-monetary sources of support. | Author Funding Statement |
| Conflicts of interest | 24 | Describe any potential for conflict of interest of study  contributors in accordance with journal policy. In the absence of a journal policy, we recommend authors comply with International Committee of Medical Journal Editors  recommendations. | Conflict Statement |

For consistency, the CHEERS Statement checklist format is based on the format of the CONSORT

statement checklist

The **ISPOR CHEERS Task Force Report** provides examples and further discussion of the 24-item

CHEERS Checklist and the CHEERS Statement. It may be accessed via the *Value in Health* link or via the ISPOR Health Economic Evaluation Publication Guidelines – CHEERS: Good Reporting Practices

webpage: <http://www.ispor.org/TaskForces/EconomicPubGuidelines.asp>

The citation for the CHEERS Task Force Report is:

Husereau D, Drummond M, Petrou S, et al. Consolidated health economic evaluation reporting standards (CHEERS)—Explanation and elaboration: A report of the ISPOR health economic evaluations publication guidelines good reporting practices task force. Value Health 2013;16:231-50.
