# Supplementary material for: When and what to test for: A cost-effectiveness analysis of febrile illness test-and-treat strategies in the era of responsible antibiotic use
Source: PLoS One. 2020 Jan 8;15(1):e0227409. doi: 10.1371/journal.pone.0227409 (PMC6948826; doi:10.1371/journal.pone.0227409)
Supplement: S1 Text — (DOCX) [file pone.0227409.s002.docx]

**S1 Text: TECHNICAL APPENDIX**

**WHEN AND WHAT TO TEST FOR: A COST-EEFFECTIVENESS ANALYSIS OF FEBRILE ILLNESS TEST-AND-TREAT STRATEGIES IN THE ERA OF RESPONSIBLE ANTIBIOTIC USE**

Authors: Anthony Zhenhuan Zhang, Diana Maria Negoescu, Claudia Munoz-Zanzi

***1. Analytic Overview***

We first studied a simplified setting where we did not consider disease progression, patient delays in seeking care, nor the impact of test costs and delays in test turnaround times. In this setting, we analytically evaluated three generic test-and-treat strategies with the goal of achieving a minimum weighted sum of penalized antibiotic underuse (i.e., the proportion of patients in the cohort presenting with antibiotic-treatable disease, but no antibiotics were prescribed) and overuse (i.e., the proportion of patients in the cohort presenting with not-antibiotic-treatable disease, but antibiotics were prescribed).

Once intuition was obtained from this simplified setting, we used Markov models that described febrile disease progression in detail to numerically estimate disability-adjusted life years (DALYs), pre-penalty costs (costs accrued prior to any antibiotic overuse penalty), and likelihood of antibiotic overuse per patient for fifteen different strategies (listed in Table 2) in two example settings in Thailand with contrasting diseases occurrence probabilities. We provide the detailed definition and estimation procedure of DALY in S1 Text, Section 3.1.3. Taking from the health system perspective, we considered pre-penalty costs accrued from diagnostic testing, antibiotic treatment, and baseline health expenditure, within the 45-day time horizon (S1 Table). The long-term consequences of antibiotic overuse, such as the development of bacterial resistance, were much more challenging to estimate since they are generally not captured within the time horizon. Consequently, we assigned a monetary penalty to every patient unnecessarily prescribed a course of antibiotics, given the disease etiology, in order to capture how different values for this weight might drive optimal decision-making.

***2. Balancing Antibiotic Overuse and Underuse***

We analyze the antibiotic overuse and underuse balancing problem in this section. We consider a simplified setting where patients presenting with fever can suffer from one of four causes:

1) a specific bacterial infection for which a test exists, and which is treatable with antibiotics (i.e., doxycycline);

2) other bacterial infections for which no test exists, but which are also treatable with the same antibiotics;

3) a specific viral infection for which a test exists (and which is not treatable with antibiotics);

4) other infections for which there is no test and are not treatable with antibiotics.

We summarize the notations for the disease occurrence probabilities in the table below：

**Table: Generic disease occurrence probability vector (sum of all probabilities is one)**

| Specified bacterial infection | Specified viral infection | Other bacterial infections | Other infections |
| --- | --- | --- | --- |
| $p_{bac}$ | $p_{viral}$ | $p_{other\_bac}$ | $p_{other}$ |

We assume there are two tests available: a bacterial test targeting the specified bacterial infection, with sensitivity ${sens}_{b}$ and specificity ${spec}_{b}$; a viral test targeting the specified viral infection with sensitivity $\mathrm{sens}_{v}$ and specificity $\mathrm{spec}_{v}$. We assume $0.5\leq sens\leq1$ and $0.5\leq spec\leq1$, and $spec\geq sens$ (i.e.,${spec}_{v}\geq{sens}_{b}$). We evaluate three types of strategies in our analysis:

1) empirical antibiotic treatment (antibiotic treatment for all patients, without any tests, henceforth “no testing” for short);

2) testing all patients using the bacterial infection test, and administering antibiotics to patients with positive results (henceforth “bacterial test” for short);

3) testing all patients using the viral infection test, and administering antibiotics to patients with negative results (henceforth “viral test” for short).

We represent the analytical expression of antibiotic underuse and overuse under each strategy in the table below:

**Table: Analytical expressions of antibiotic overuse and underuse**

| Strategy list | | $P(under)$ | $P(over)$ |
| --- | --- | --- | --- |
| 1 | “no testing” | 0 | ${(p}_{viral}+p_{other})$ |
| 2 | “bacterial test” | $p_{bac}\left( 1-sens_{b} \right)+p_{other_{bac}}{spec}_{b}$ | ${(p}_{viral}+p_{other})(1-spec_{b})$ |
| 3 | “viral test” | ${(p}_{bac}+p_{other_{bac}})(1-spec_{v})$ | $p_{viral}\left( 1-{sens}_{v} \right)+p_{other}spec_{v}$ |

We select the strategy $i$with the minimum weighted average between $Prob(under)$ and $Prob(over)$:

$$\min_{i} w_{under}Prob\left( under \right)_{i}+w_{over} Prob\left( over \right)_{i}$$

***2.1. Pairwise Strategy Evaluation***

In this section, we perform pairwise strategy evaluation by comparing the weighted average of $Prob(under)$ and $Prob\left( over \right)$ between any two strategies. Strategy “bacterial test” has a smaller objective value compared to “no testing” when the following condition is satisfied:

$$\left( \frac{w_{under}}{w_{over}}\cdot\frac{\left( 1-{sens}_{b} \right)}{{spec}_{b}}+1 \right)p_{bac}+\left( \frac{w_{under}}{w_{over}}+1 \right)p_{{other}_{bac}}\leq1 (2)$$

Similarly, Strategy “viral test” gives a smaller objective value compared to “no testing” when the following condition is satisfied:

$$\left( \frac{w_{over} {sens}_{v}}{w_{under}\left( 1-{spec}_{v} \right)}+1 \right)p_{viral}+\left( \frac{w_{over}}{w_{under}}+1 \right)p_{other}\geq1 (3)$$

We have also compared between the two testing strategies. “bacterial test” provides a smaller objective value compared to “viral test” when the following condition is satisfied (by comparing the analytical objective values):

$${-\frac{w_{under}}{w_{over}}p}_{bac}{sens}_{b}+p_{viral}{sens}_{v}+\left( \frac{w_{under}}{w_{over}}p_{{other}_{bac}}-p_{other}-p_{viral} \right){spec}_{b}+\left( \frac{w_{under}}{w_{over}}\left( p_{{other}_{bac}}+p_{bac} \right)-p_{other} \right)spec_{v}-\left( \frac{w_{under}}{w_{over}}p_{{other}_{bac}}-p_{other} \right) \leq0 (4)$$

On the other hand, “viral test” provides a smaller objective value compared to “bacterial test” when the following condition is satisfied:

$${-\frac{w_{under}}{w_{over}}p}_{bac}{sens}_{b}+p_{viral}{sens}_{v}+\left( \frac{w_{under}}{w_{over}}p_{{other}_{bac}}-p_{other}-p_{viral} \right){spec}_{b}+\left( \frac{w_{under}}{w_{over}}\left( p_{{other}_{bac}}+p_{bac} \right)-p_{other} \right)spec_{v}-\left( \frac{w_{under}}{w_{over}}p_{{other}_{bac}}-p_{other} \right)\geq0 (5)$$

We simplify the first two inequalities above to determine the disease distribution condition (such that the two testing strategies outperform “no testing”) for any given weights. We note that the left-hand side (henceforth LHS) of Inequality (2) is bounded above (reaches its maximum) by setting is ${sens}_{b}$ and ${spec}_{b}$ to the smallest possible values (50% each):

$\max\left( \frac{w_{under}}{w_{over}}\cdot\frac{\left( 1-{sens}_{b} \right)}{{spec}_{b}}+1 \right)p_{bac}=\left( \frac{w_{under}}{w_{over}}+1 \right)p_{bac}$

Then, we have the following simplified condition:

$$p_{bac}+p_{{other}_{bac}}\leq\frac{w_{over}}{w_{over}+w_{under}} (6)$$

Similarly, we simplify Condition (3) to

$p_{viral}+p_{other}\geq\frac{w_{under}}{w_{over}+w_{under}} (7)$

In addition, note that $p_{bac}+p_{{other}_{bac}}+p_{viral}+p_{other}=1$. Then, we can rewrite Condition (7) as: $1-\frac{w_{under}}{w_{over}+w_{under}}\geq p_{bac}+p_{{other}_{bac}},$ which is the same as Condition (6).

We then derive sufficient conditions (without test sensitivity and specificity requirements) for “bacterial test” to outperform “viral test”, and vice versa. We first make the following observations about the LHS (the same for Inequality (4) and (5)):

1. $\mathrm{sens}_{b}$ has a non-positive coefficient.
2. $\mathrm{sens}_{v}$ has a non-negative coefficient.
3. if $\frac{w_{under}}{w_{over}}\left( p_{{other}_{bac}}+p_{bac} \right)\leq p_{other}$ (denoted as * henceforth), the coefficient of $\mathrm{spec}_{v}$ is non-positive (otherwise positive).
4. if $\frac{w_{under}}{w_{over}}\left( p_{{other}_{bac}} \right)>p_{other}+p_{viral}$ (denoted as $^{\circ}$ henceforth), the coefficient of $\mathrm{spec}_{b}$ is positive (otherwise non-positive).

We claim that (*) and ($^{\circ}$) are mutually exclusive. To see this, first note that all disease occurrence probabilities and the weight ratio are non-negative. Then, given ($^{\circ}$), we have:

$\frac{w_{under}}{w_{over}}\left( p_{{other}_{bac}}+p_{bac} \right)\geq\frac{w_{under}}{w_{over}}\left( p_{{other}_{bac}} \right)>p_{other}+p_{viral}\geq p_{other}$*,*

which leads to $\frac{w_{under}}{w_{over}}\left( p_{{other}_{bac}}+p_{bac} \right)>p_{other}$. This is a contradiction to (*).

Similarly, given (*), we have

$$\frac{w_{under}}{w_{over}}\left( p_{{other}_{bac}} \right)\leq\frac{w_{under}}{w_{over}}\left( p_{{other}_{bac}}+p_{bac} \right)\leq p_{other}\leq p_{other}+p_{viral}$$

which leads to $\frac{w_{under}}{w_{over}}\left( p_{{other}_{bac}} \right)\leq p_{other}+p_{viral}$, a contradiction to ($^{\circ}$).

Now, suppose (*) is true. Then, from the LHS of inequality (4), we have

${-\frac{w_{under}}{w_{over}}p}_{bac}{sens}_{b}+p_{viral}{sens}_{v}+\left( \frac{w_{under}}{w_{over}}p_{{other}_{bac}}-p_{other}-p_{viral} \right){spec}_{b}+\left( \frac{w_{under}}{w_{over}}\left( p_{{other}_{bac}}+p_{bac} \right)-p_{other} \right)spec_{v}-\left( \frac{w_{under}}{w_{over}}p_{{other}_{bac}}-p_{other} \right)$

$$\leq{-\frac{w_{under}}{w_{over}}p}_{bac}{sens}_{b}+\left( \frac{w_{under}}{w_{over}}p_{{other}_{bac}}-p_{other} \right){spec}_{b}+\left( \frac{w_{under}}{w_{over}}\left( p_{{other}_{bac}}+p_{bac} \right)-p_{other} \right)spec_{v}-\left( \frac{w_{under}}{w_{over}}p_{{other}_{bac}}-p_{other} \right)$$

$$\leq{-\frac{w_{under}}{w_{over}}p}_{bac}0.5+\left( \frac{w_{under}}{w_{over}}p_{{other}_{bac}}-p_{other} \right)0.5+\left( \frac{w_{under}}{w_{over}}\left( p_{{other}_{bac}}+p_{bac} \right)-p_{other} \right)0.5-\left( \frac{w_{under}}{w_{over}}p_{{other}_{bac}}-p_{other} \right)=0$$

The first inequality follows because $p_{viral}\left( {sens}_{v}-{spec}_{b} \right)\leq0$, the second inequality follows by setting $\mathrm{sens}_{b}=spec_{b}={spec}_{v}=50\%$ (i.e., smallest possible values) since they all have non-positive coefficients.

Then, under (*), we have the LHS of (4) bounded above by 0. Thus, (*) is a sufficient condition to (4).

We also derive the sufficient guarantee of “viral test” outperforming “bacterial test”.

Now suppose ($^{\circ}$) is true. From the LHS of inequality (5), we have

${-\frac{w_{under}}{w_{over}}p}_{bac}{sens}_{b}+p_{viral}{sens}_{v}+\left( \frac{w_{under}}{w_{over}}p_{{other}_{bac}}-p_{other}-p_{viral} \right){spec}_{b}+\left( \frac{w_{under}}{w_{over}}\left( p_{{other}_{bac}}+p_{bac} \right)-p_{other} \right)spec_{v}-\left( \frac{w_{under}}{w_{over}}p_{{other}_{bac}}-p_{other} \right)$

$$\geq p_{viral}{sens}_{v}+\left( \frac{w_{under}}{w_{over}}p_{{other}_{bac}}-p_{other}-p_{viral} \right){spec}_{b}+\left( \frac{w_{under}}{w_{over}}p_{{other}_{bac}}-p_{other} \right)spec_{v}-\left( \frac{w_{under}}{w_{over}}p_{{other}_{bac}}-p_{other} \right)$$

$$\geq p_{viral}0.5+\left( \frac{w_{under}}{w_{over}}p_{{other}_{bac}}-p_{other}-p_{viral} \right)0.5+\left( \frac{w_{under}}{w_{over}}p_{{other}_{bac}}-p_{other} \right)0.5-\left( \frac{w_{under}}{w_{over}}p_{{other}_{bac}}-p_{other} \right)=0$$

The first inequality follows since $-{\frac{w_{under}}{w_{over}}p}_{bac}\left( {sens}_{b}-{spec}_{v} \right)\geq0$, the second inequality follows by setting ${sens}_{v}=spec_{b}=spec_{V}=50\%$ (similar reason as before). Then, we conclude that the LHS of (5) is bounded below by 0 given ($^{\circ}$). Thus, ($^{\circ}$) is a sufficient condition to (5).

As a conclusion, we showed that (*) is a sufficient condition on “bacterial test” outperforming “viral test”, and ($^{\circ}$) is a sufficient condition on “viral test” outperforming “bacterial test”.

***3. Febrile Disease Progression Model***

We developed detailed Markov cohort models to capture febrile disease progression and used the model outcomes (DALYs, costs, antibiotic overuse) to evaluate alternative strategies for diagnosis and antibiotic treatment. We modeled a hypothetical cohort of 40-year-old adult patients (age varied in sensitivity analysis) with acute, undifferentiated fever since symptom onset. We set the time horizon to 45 days since most patients would be either recovered or deceased by then. We tracked patient health states daily. On each day, patients could recover, progress to severe disease stage or die. Patients presented to hospitals on their first, fourth or tenth day of illness, which captured the minimum, average, and maximum of time for patients presenting to hospitals seeking care. We simplified the health states for any infection type to four core states: Mild, Severe, Recovered and Death. The exact daily transition probabilities depended upon the specific infection etiology and treatment (i.e., with or without antibiotics, S1 Table). We assumed that all Severe patients were immediately hospitalized and assigned to a diagnostic and treatment strategy if it was the first hospital visit (no prior testing). We also assumed that deaths during the 45-day time horizon were caused only by severe complications of infections.

We provided the patient flow diagram through our model in S1 Fig. Patients presented to the hospital (in either Mild or Severe state) for the first time were prescribed one of the strategies listed in Table 2. Patients who progressed to Severe state (i.e., they demonstrate various clinical signs in severe febrile illness) were assumed to be hospitalized. Because the purpose of the testing strategies was to inform antibiotic treatment (not for disease diagnosis or confirmation), we assumed that antibiotics would only be prescribed to patients when all pending test results (if any) were obtained. A positive bacterial test result or a negative viral test result would generally lead to the prescription of antibiotics, whereas a negative bacterial test result or a positive viral test result would not.

***3.1. Model Calibration***

***3.1.1. Transition Probabilities***

Transition probabilities among health states were time-homogeneous. Transition probabilities for each disease were calibrated to match the mean duration of fever in Mild state, and mean duration of hospitalization in Severe state, and mortality rates reported in the medical literature. We assumed that antibiotic treatment reduced mean illness duration (mean days of hospitalization) by half for mild (severe) leptospirosis and scrub typhus patients [1, 2]. We also assumed that antibiotic treatment could prevent Mild patients progressing to Severe states [1] (i.e., 100% antibiotic effectiveness). We varied this assumption in S1 Text, Section 6.2. Transition probabilities for “other” infections not treatable with antibiotics were assumed to be similar to dengue, and were varied in sensitivity analysis. We provide a sample Markov trace plot for leptospirosis disease progression in S2 Fig. Exact values and references for each parameter are provided in S1 Table.

***3.1.2. Costs, DALYs, test sensitivities and specificities***

We obtained test costs, health costs and DALY-related parameters for all five possible causes of infection from the published literature and WHO life table for Thailand [3]. Disability weights (DW) for “other bacterial” and “other” infections were assumed to be of the same magnitude as dengue, leptospirosis and scrub typhus (i.e., DW in Mild state: 0.2; DW in Severe state: 0.5) [4, 5]. Sensitivities and specificities of leptospirosis, scrub typhus and dengue diagnostic tests were reported in various studies [6-11]. Detailed references can be found in S1 Table. Due to the lack of studies reporting sensitivity and specificity of multiplex PCR tests in Thailand, we set the sensitivity and specificity of multiplex PCR diagnostic test (except for the scrub typhus component [12]) to be similar (but slightly lower in order to be conservative) than values reported for other places in the medical literature [13, 14].

***3.1.3. DALY Calculation and Estimation***

DALY, disability-adjusted life years, is a measure of overall disease burden. We first calculate the Years of Life Lost (YLL) due to premature mortality in the population, then, we compute the Years Lost due to Disability (YLD) for people living with the disease. The total DALY is calculated by taking the sum of YLL and YLD: DALY = YLL + YLD. Specifically, the YLL equals to the number of deaths multiplied by the standard remaining life expectancy given patient age at death. To estimate YLD for a particular disease in a given time horizon, the number of incident cases in that period is multiplied by the average duration of the disease and a weight factor (disability weight) that reflects illness severity on a scale from 0 (perfect health) to 1 (dead) [15].

As an example, if a 40-year-old male dengue patient (in Thailand) spent 10 days in Mild state (disability weight, DW = 0.2), and 10 days in Severe state (DW = 0.5), and dead afterwards: We have YLL = 34.9 (expected life expectancy is 74.9 in Thailand [15]), and YLD = $\frac{10}{365}\times\left( 0.2+0.5 \right)=0.019$. Then DALY = 34.9 + 0.019 = 34.919.

In the Result Section, we show the per-patient (expected) DALYs incurred where we divide the total DALYs incurred in the population by the population size.

***3.2. Determining Weights of Antibiotic Overuse/Underuse***

In this section, we provide the estimation procedure to inform the weights of antibiotic overuse and underuse (i.e., $w_{\mathrm{over}}\mathrm{and}w_{\mathrm{under}}$), where the goal is to compute the weighted sum of antibiotic overuse and underuse (S1 Text, Section 2).

For each antibiotic-treatable (bacterial) disease, we first computed the difference in estimated monetary and clinical outcomes (i.e., costs, DALYs) by our disease progression model between the strategy with and without antibiotic treatment. In particular, for patients prescribed with antibiotics, treatments were assumed to be initiated on the first day of illness, to better capture the difference in health outcomes. We then followed the classical approach in healthcare economics to combine the two objectives (incremental costs, DALYs averted, or equivalently cost, DALY difference) into a single net monetary benefit (NMB) objective (which is the weight $w_{\mathrm{under}}$) on cost scale by associating a willingness-to-pay (WTP) threshold per DALY averted: $w_{\mathrm{under}}$ = WTP $\times$DALYs averted – incremental costs. In Thailand setting, we choose WTP to be Thailand GDP per capita in 2016: 5,907.91 USD. Then, we estimated $w_{\mathrm{under}}$ for leptospirosis: 25777 USD; scrub typhus: 25780 USD; and other bacterial: 25765 USD. We assumed a single weight (denoted by $w_{under}$) for all antibiotic-treatable diseases: 25777 USD (the median) in our case study.

For non-bacterial diseases, the health burden of over-prescribing antibiotics was not captured within the 45-day study horizon. We represented the weight using a monetary penalty (denoted by $w_{over}$, and the base value was set to the WTP level used when calculating$w_{under}$) on every course of antibiotics (over)prescribed to non-bacterial patients. There have been studies investigating the cost of resistance by consuming antibiotics. For example, Shrestha et al. [16] reported the estimated economic cost of resistance per standard unit (per course) of penicillin (an alternative antibiotic to treat febrile bacterial diseases) was $0.8 ($11.5) in Thailand. Nevertheless, the model might underestimate the cost of resistance to a considerable degree. As discussed in [16], their model does not take transmission and future medical cost into account. Second, their model only counts a subset of resistant infections, and one death only results in 10 life years lost. More importantly, their definition of the estimated cost of resistance is different from our definition of antibiotic overuse penalty: we only penalize unnecessary antibiotics prescription, not including those patients with a bacterial disease whom should indeed receive antibiotic therapy. A higher penalty translates to a higher priority in antibiotics overuse reduction by preventing possible future harm due to antibiotics resistance, not the myopic healthcare costs immediately incurred for patients.

We computed the test sensitivity requirements for testing strategies to dominate “no testing” in Scenario A and B (by grouping scrub typhus under the “other bacterial” category). We set $w_{\mathrm{over}}$= 5,907.91 USD (Thailand GDP per capita in 2016), and $w_{\mathrm{under}}=25777 USD$. For Scenario A (bacterial-endemic), model results showed that, even if the bacterial or the viral test has 100% sensitivity, empirical antibiotic treatment to all patients (without any test) was optimal over any testing strategy. On the other hand, testing strategies involving a bacterial test with any sensitivity value or a viral test with sensitivity$\geq8.76\%$ (most tests have sensitivities $\geq50\%$) would be better than empirical antibiotic treatment in Scenario B (viral-endemic).

***4. Three-dimensional Cost-effectiveness Analysis***

In this section, we discuss the form of the augmented cost-effectiveness analysis (three-dimensional cost-effectiveness analysis evaluating outcomes in terms of DALYs, pre-penalty costs, and antibiotic overuse). Previous work on cost-effectiveness analyses tends to focus on two-dimensional outcomes by considering only the trade-off between health benefits (i.e., QALYs gained or DALYs averted) and costs [17]. However, as antibiotic overuse has been identified as one of the key drivers for antimicrobial resistance, its long-term consequences (such as antimicrobial resistance development) should be considered when performing economic evaluation of public health policies. More importantly, for our problem, without considering the dimension of “antibiotic overuse” - a primary focus on our study, Strategy 2 (Empirical antibiotics to all patients) will dominate all other strategies by incurring the least DALYs and costs. We expand the standard cost-effectiveness analysis by considering a third dimension of non-direct health outcomes (i.e., amount of antibiotic overuse) in the economic evaluation of health policies. This enables health policy makers to analyze the economic value of febrile illness test-and-treat strategies while keeping track of antibiotic usage.

Our definition of three-dimensional cost-effectiveness analysis shares similarity with the so-called extended cost-effectiveness analysis, developed to evaluate the consequences of health policies in the domains of relative costs, health outcomes, and financial risk protection (non-health benefits [18, 19]. Instead of financial risk protection (a third dimension that does not exist in standard cost-effectiveness analysis), we consider the penalized antibiotic overuse, which is a potential harm in health outcomes (years of life lost, additional deaths, etc.) that could not be captured within the time horizon (45 days).

To combine the three objectives (DALYs, costs, and antibiotic overuse) into a single objective, we expanded the classical approach in healthcare economics which combines the two objectives (incremental costs, DALYs averted) into a single net monetary benefit (NMB) objective on a cost scale. First, we define DALYs averted for a given strategy as the difference between the DALYs incurred by the strategy and those incurred by the “No Antibiotics” strategy. We defined the incremental costs and the incremental *Prob(over)* similarly. Second, we associate a willingness-to-pay (WTP) threshold per DALY averted, and a penalty ($w_{over}$) per unnecessary course of antibiotics. Then, we compute the NMB as:

$$NMB=(DALYs averted)\times WTP-(incremental costs)-w_{over} (incremental Prob\left( over \right)).$$

By considering a range of values for willingness-to-pay per DALY averted, and for penalty per unnecessary course of antibiotics, we first compute the three-dimensional effectiveness frontier (S1 Text, Section 5), then quantify the ranges of the NMB of each strategy.

***5. Linear Program to identify the 3-Dimensional Pareto Frontier***

In this section, we develop a method to determine whether a given strategy output is on the three-dimensional (3D) Pareto Frontier (i.e., the effectiveness frontier) in terms of DALYs, costs, and antibiotic overuse. As described in Section 4, when only considering the tradeoff between health benefits and costs, empirical antibiotics to all patients (Strategy 2 in Table 2) dominated all other strategies with the least DALYs and costs. However, when considering the trade-offs in three dimensions – costs, DALYs, and antibiotic overuse, different strategies may appear on the 3D Pareto frontier.

To identify the three-dimensional Pareto frontier, we employ the idea that a given point (i.e., strategy output consisting of the tuple (DALYs, costs, antibiotics overuse)) is on the frontier if and only if the convex set formulated by perturbations of this point in one improving direction (i.e., smaller DALYs, costs or antibiotic overuse) is disjoint from the convex hull determined by the original set of points (including the point from which the perturbation was obtained).

We define $e_{j}$ as the unit vector along *j*th dimension in $R^{3}$ (i.e. $e_{2}=\left( 0,1,0 \right)$ ). Let $p_{i,j}$ represent the model output from strategy $i$ along $j$th dimension (“DALYs”, “costs”, or “antibiotics overuse”) and $p_{i}$ be the three-dimensional output. Denote $P$ as the set of all outputs. We then define the convex hull formulated from $P$ as $Conv\left( P \right)=\left\{ \sum_{k=1}^{n} \alpha_{k}p_{k}|(\forall k:\alpha_{k}\geq0)\wedge\sum_{k=1}^{n} \alpha_{k}=1,n=\left| P \right| \right\}$ in the “DALYs”, “costs” and “antibiotics overuse” space. For each $p_{i}$, we first construct three points ($q_{i}^{\left( 1 \right)},q_{i}^{\left( 2 \right)},q_{i}^{\left( 3 \right)}$) where $q_{i}^{\left( j \right)}=p_{i}$ – $\epsilon e_{j}, j=1, 2, 3$: subtract a sufficiently small positive $\epsilon$from “DALYs”, “costs” and “antibiotics overuse” dimensions respectively (i.e. $\epsilon$ should be much smaller than the minimum pairwise difference in any dimension: $\epsilon<\min{|p}_{i,j}-p_{i^{'},j}|, \forall i,i^{'}=1,\ldots,n, j=1, 2, 3$). Denote $Q_{i}=\{q_{i}^{\left( j \right)}|j=1,2,3\}$. We then construct the convex set $\mathrm{Conv}\left( Q_{i} \right)=\left\{ \sum_{l=1}^{3} \beta_{l}q_{i}^{\left( l \right)}|(\forall l:\beta_{l}\geq0)\wedge\sum_{l=1}^{3} \beta_{l}=1 \right\}$. The intuition behind $\mathrm{Conv}\left( Q_{i} \right)$ is to construct a convex set containing points in the space that dominates $p_{i}$ in one or more dimensions. By the separating hyperplane theorem [20], we know that if two convex sets are disjoint, there exists a hyperplane $G=\left\{ x \epsilon R^{3}:z^{T}x= z_{0} \right\}$ that separates one set from the other. Without loss of generality, we assume $\left| P \right|=n<\infty$. Then, both $Conv(P)$ and $Conv\left( Q_{i} \right)$ are polytopes (i.e., convex hulls of finite number of points).

We formulate the following feasibility testing linear program to find a separating hyperplane between $Conv\left( Q_{i} \right)$ and $Conv\left( P \right)$. This hyperplane (defined by decision variable $\left( z_{0},z \right)$ with $z_{0}\in R,z\in R^{3}$), if it exists, restricts $Conv(P)$ to be at one side (i.e. satisfying the first inequality), and $Conv\left( Q_{i} \right)$ at another side (i.e., satisfying the second inequality) of the hyperplane.


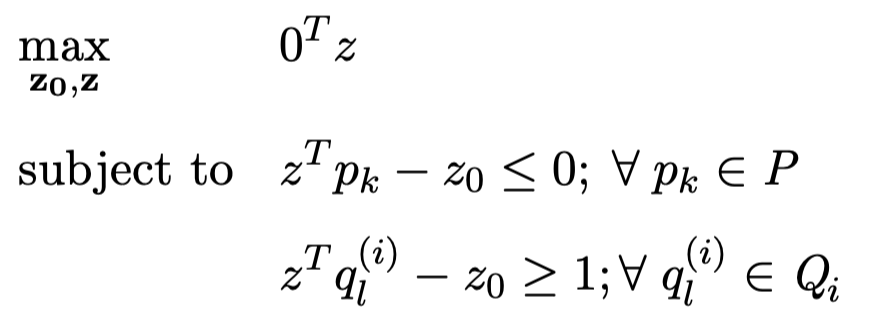


*Theorem:*

Strategy output $p_{i}$ is on the 3-D Pareto Frontier if and only if there exists (at least) one feasible solution ($z_{0},z$) defining a hyperplane which separates $Conv(P)$ and $Conv\left( Q_{i} \right).$

Proof

We use the definition of Pareto optimality from [20], page 177: Given a set of feasible points *P*, a point $p_{i}\in R^{3}$ is Pareto optimal if and only if $\left( p_{i}+R_{-}^{3} \right)\cap P=\{p_{i}\}$. The convex cone $\left( p_{i}+R_{-}^{3} \right)$ can be interpreted as the set containing points whose values that are smaller than or equal to $p_{i}$. The above condition states that the only point that is smaller than or equal to $p_{i}$ in set $P$ should be $p_{i}$itself. We would like to show the condition $\left( p_{i}+R_{-}^{3} \right)\cap P=\{p_{i}\}$ is equivalent to $Conv\left( Q_{i} \right)\cap Conv\left( P \right)=\emptyset$*.*

Proof of the forward direction: We first show $\left( p_{i}+R_{-}^{3} \right)\cap P=\left\{ p_{i} \right\}$ leads to $\left( p_{i}+R_{-}^{3} \right)/\{p_{i}\}\cap Conv(P)=\emptyset$. This can be proved by contradiction: suppose $\left( p_{i}+R_{-}^{3} \right)/\{p_{i}\}\cap Conv(P)\neq\emptyset$*,* since $Conv(P)$ is a polytope, there must exist at least one vertex of $Conv(P)$ (and thus in set $P$) inside the set $\left( p_{i}+R_{-}^{3} \right)/\{p_{i}\}$, which contradicts our assumption. At the same time, by definition of $Conv\left( Q_{i} \right)$,we know that$Conv\left( Q_{i} \right)$ is a subset of $\left( p_{i}+R_{-}^{3} \right)/\{p_{i}\}$. Together with $\left( p_{i}+R_{-}^{3} \right)/\{p_{i}\}\cap Conv(P)=\emptyset$, we conclude $Conv\left( Q_{i} \right)\cap Conv\left( P \right)=\emptyset$*.* Q.E.D.

Proof of the reverse direction: (prove by contradiction) Suppose $\left( p_{i}+R_{-}^{3} \right)/\{p_{i}\}\cap P\neq\emptyset$. Let $p_{j}$ be one of the points in the intersection. Since we restricted $\epsilon$ should be smaller than the minimum pairwise difference in any dimension (i.e., $\epsilon<\min{|p}_{i,j}-p_{i^{'},j}|, \forall i,i^{'}=1,\ldots,n, j=1, 2, 3$)), then the line segment ${\lambda p}_{i}+\left( 1-\lambda\right)p_{j},0\leq\lambda\leq1$ intersect with $\mathrm{Conv}\left( Q_{i} \right)$. By definition of $\mathrm{Conv}\left( P \right)$, we have ${\lambda p}_{i}+\left( 1-\lambda\right)p_{j}\subset\mathrm{Conv}\left( P \right)$. Together, these two conditions lead us to $\mathrm{Conv}\left( Q_{i} \right)\cap Conv\left( P \right)\neq\emptyset$. Given $\left( p_{i}+R_{-}^{3} \right)/\{p_{i}\}\cap P\neq\emptyset\Rightarrow Conv\left( Q_{i} \right)\cap Conv\left( P \right)\neq\emptyset$, we have $\mathrm{Conv}\left( Q_{i} \right)\cap Conv\left( P \right)=\emptyset. \Rightarrow\left( p_{i}+R_{-}^{3} \right)\cap P=\{p_{i}\}$. Q.E.D.

***6. Sensitivity Analysis***

We performed sensitivity analyses to evaluate the robustness of our results. We refer the analysis in the main manuscript, numerical results section as the base-case analysis.

***6.1. Varying Disease Occurrence Probability***

We performed sensitivity analysis on the level endemicity of different diseases at different locations in Thailand [6] (i.e., Set 1 and Set 2 in S5 Table). We then computed the optimal strategy sequence for Set 1 and Set 2 when patients present to hospitals at their first, fourth and tenth day of illness (S3 Fig and S4 Fig respectively).

We have the followings observations from Figures S3 Fig and S4 Fig. First, the main observations were consistent with the base-case analysis: Empirical antibiotic treatment to all patients (Strategy 2) had the highest NMB at small antibiotics overuse penalty values. Depending on the specific disease occurrence probability and WTP level, the threshold (for a strategy to be optimal in terms of NMB) may vary. Second, as the probability of dengue (or equivalently, viral-diseases) increases, single-test strategies (i.e., Dengue PCR or Dengue RDT, or Lepto RDT) had the highest NMB for a wider range of WTP and penalty values. Third, when WTP equals the Thai GDP per capita, the optimal strategy sequence was consistent with the base-case: Empirical antibiotics to all patients was optimal in low penalty region. Strategy 15 (Multiplex PCR test) became optimal for penalty $\geq$ $13,000/course and $\geq$ 20,000/course for patients presenting on day one and day four, respectively. Strategy 12 (P: Lepto RDT, typhus RDT) became optimal for penalty $\geq$30,000/course on day ten with Set 1 disease occurrence probability vector. With Set 2 disease occurrence probability vector, and Strategy 15 (Multiplex PCR test) became optimal for penalty $\geq$6,000/course and $\geq$ 8,500/course, for patients presenting on day one, and day four, respectively. Strategy 12 (P: Lepto RDT, typhus RDT) became optimal for penalty $\geq$$15,000/course if presenting on day ten. Lastly, when patients present late (i.e., on the tenth day of illness), strategies with rapid diagnostic tests such as Strategy 4 (dengue RDT), Strategy 12 (P: Lepto RDT, typhus RDT) and Strategy 13 (P: Lepto PCR, typhus RDT) became optimal. This is also consistent with our base case analysis since RDT is more accurate in the later stage of diseases.

***6.2. Varying Antibiotic Effectiveness***

We made the simplifying assumption in our model that all severe cases of antibiotic-treatable infections can be prevented by initiating antibiotic treatment in Mild states (i.e., 100% antibiotic effectiveness). This has been reported in the literature [1]. However, in this section, we varied this assumption by assuming 50%, and 75% antibiotic effectiveness in preventing progression to Severe state in Mild state patients. We kept all other parameters the same as in the base-case.

The per-patient costs incurred (in USD), health burden (in DALY), and antibiotics overuse ($Prob(over)$), underuse ($Prob(under)$) for each strategy are shown in Table 1, S6 Table, S7 Table for patients seeking care on the fourth day of illness (average day of presentation), under different antibiotic effectiveness scenarios. From these tables, we found that as antibiotic effectiveness decreases, costs and DALYs averted increases for all strategies except for strategies with no antibiotic treatment (Strategy 1) or antibiotic treatment for only Severe state patients (Strategy 3). $Prob(over)$), and $Prob(under)$) remained the same with varying antibiotic effectiveness. For clarity purpose, we did not show the table results for patients seeking care on the first, and the tenth day of illness. The conclusion was similar.

Figures S7 – S12 Fig visualize the resulting cost-effectiveness (CE) plots with different days of presentation and disease occurrence probabilities. From these figures, we observed that strategies on the cost-effectiveness frontier generally remained the same as in the base-case analysis. When patients presenting on day ten (S9 Fig and S12 Fig), the cost-effectiveness frontier remained exactly the same. As the benefit of early antibiotic treatment diminishes (i.e., as antibiotic effectiveness to prevent progression to Severe state decreases), empirical antibiotic treatment to all patients (Strategy 2) became optimal (in terms of NMB) for a narrower range of $w_{over}$ and WTP, regardless of the disease probability distribution and the day of presentation to hospital (Figures S7 – S12 Fig). The change is more obvious in bacterial-endemic Scenario A than in viral-endemic Scenario B, since there is a larger portion of antibiotic-treatable patients under Scenario A. On the other hand, with reduced antibiotic effectiveness, empirical antibiotics to only Severe patients (Strategy 3) became optimal for a wider range of $w_{over}$ and WTP for patients present on day one and day four (Figures S7 – S8 Fig and S10 – S11 Fig).

Our base-case analysis assumed 100% effectiveness of antibiotic treatment to prevent progression to Severe state. As antibiotic effectiveness was reduced, testing strategies, or strategies with non-aggressive use of antibiotics (Strategy 3) became optimal for wider ranges of $w_{over}$ and WTP, compared to the base-case. This was because the reduced antibiotic effectiveness led to a reduced importance of antibiotic underuse. As a consequence, a reduced antibiotic effectiveness makes empirical antibiotic treatment to all patients (Strategy 2) less preferable.

***6.3. Remaining One-way Sensitivity Analysis***

We performed one-way sensitivity analysis for all variables other than the disease occurrence probabilities over the estimated data ranges (S1 Table). We kept the disease occurrence probabilities the same as in bacterial-endemic Scenario A (i.e., 52.8% chance of leptospirosis infection, Table 1) and assumed patients present to hospitals on their first day of illness. We display optimal strategies at penalty ($w_{\mathrm{over}}$) = $ 0, $10,000, and $50,000 in S2 Table. In all cases, empirical treatment was optimal in terms of NMB if we only consider DALYs and costs incurred (i.e., penalty = $0).

The results were sensitive to a few variables, including cohort age, test turnaround time (wait time), as well as the sensitivity and specificity of diagnostic tests. For a cohort of younger patients, empirical antibiotic treatment to all patients is more beneficial by avoiding high DALYs caused by deaths. For a cohort of older patients (i.e., 60 years old), Strategy 15 (Multiplex PCR) is optimal with a moderate penalty in antibiotic overuse. In bacterial-endemic Scenario A, our analyses show that strategies consisting of high sensitivity and specificity leptospirosis diagnostic had a higher chance of being optimal in leptospirosis-endemic settings.

Our results were not sensitive to disease progression probabilities, disability weights (DWs) and treatments costs or productivity loss costs in either Mild or Severe state. This is because even though values of DWs, costs, transition probabilities were varied, they were still on different magnitudes for different health states (i.e., the minimum DW in Severe state is higher than the maximum DW in Mild state). Among all variable ranges considered, Strategy 2 (Empirical All), 15 (Multiplex PCR test) were generally on the 3D effectiveness frontier. These results imply that Multiplex PCR test can be of value when antibiotic overuse is a concern.

We observed from Figure 1 and Figure 2 that empirical antibiotic treatment (Strategy 2) and Multiplex PCR (Strategy 15) were optimal in terms of NMB for a wide range of $w_{over}$ and WTP. We then restricted our attention by comparing the relative cost-effectiveness of these two strategies (S4 Fig). We define the augmented incremental cost-effectiveness ratio (Augmented ICER) as the ratio of DALY difference and augmented cost difference, where augmented cost is the sum of all cost incurred, plus the probability of antibiotic overuse times the penalty (i.e., augmented cost = $costs+w_{over}\times Prob\left( over \right)$ ).

We found that the empirical antibiotic treatment to all was always on the 3D effectiveness frontier, and resulted in a smaller augmented ICER (and therefore it was preferred when compared against Multiplex PCR) at younger patient age, lower leptospirosis-component sensitivity, higher disease-specific mortality rate, and longer test turnaround time. The remaining parameters, such as test and health costs, and disability weights were not sensitive to variation. All these conclusions were consistent with the results shown in S2 Table.

***6.4. Probabilistic Sensitivity Analysis***

We conducted a probabilistic sensitivity analysis using Monte Carlo simulation, examining the cost-effectiveness of each strategy for each set of sampled parameters. The willingness-to-pay threshold was set to be Thailand GDP per capita in 2016: 5907.91 USD. We randomly generated 10,000 problem instances (i.e., 10,000 scenarios with different parameter sets). For each instance, all parameters that are on a continuous scale were randomly sampled from a triangle distribution with mode given by the base case value, lowest and highest values corresponding to the ranges in S1 Table. The disease occurrence probabilities were randomly generated given the ranges of values reported in [6]. Parameters that are on discrete scales (i.e., the turnaround time of diagnostic tests) were sampled from either triangle distributions (rounded to the nearest integer) or uniform distributions.

The Monte Carlo simulation results are shown in S6 Fig. Our analyses indicated that when the penalty on antibiotic overuse is low ($0/course to $10,000/course), empirical treatment was most likely to be optimal in terms of NMB, while strategies with single dengue tests (Strategy 5, Dengue PCR and Strategy 4, Dengue RDT) could be alternatives to reduce antibiotic overuse. However, as the penalty increased, strategies involving bacterial tests started to dominate the empirical treatment strategy. For penalty between $25,000/course to $150,000/course, Strategy 15 (Multiplex PCR) was optimal in terms of NMB. Other than Strategy 15, Strategy 11 (P: Lepto PCR, typhus PCR) had a higher likelihood of rendering the highest NMB compared to other strategies at a moderate or high penalty on antibiotic overuse. Similar to previous analyses, once antibiotic reduction was prioritized (i.e., penalty $\geq$ $150,000 course), strategies with single leptospirosis PCR test (Strategy 7) would eventually attain the highest NMB. We observe in PSA analysis that PCR-led strategies dominated RDT-led strategies even when patients’ health seeking behavior (delay) were heterogeneous (min: 0 day, mean: 4 days, max: 10 days). Observing the descriptive statistics from S1 Table, we make the observation that PCR is more accurate (higher sensitivity and specificity) during the early days of illness, whereas RDT is more accurate in the later stage of diseases. However, patients gained much more benefit if tested early and antibiotic treatment was initiated early before they progressed to worsened health states. Consequently, even though we varied the delay of seeking care in PSA analyses, more health benefits were accrued from early detection and early antibiotic treatment. This is consistent with findings from another study [21]. Meanwhile, at the average day of presentation (fourth day of illness), PCR had better performance than RDT (i.e., higher sensitivities and specificities). Lastly, Empirical antibiotics to only Severe patients can be optimal in terms of NMB with at most 35% chance at penalty = $15,000/course.

***7. Considering four additional dengue-led strategies***

In settings where viral disease is more prevalent, healthcare decision-makers and administrators might consider sequential test strategies that include viral disease tests. In this section, we consider four additional sequential strategies in which patients are tested with a dengue test first, and if negative, patients are tested for leptospirosis [22, 23] (see S8 Table for strategy description). We did not consider parallel testing of dengue and leptospirosis because testing for the two diseases simultaneously does not provide additional gains over the single test strategy scenario while avoiding the extra test costs (i.e., the treatment decision relies on a single test result)

Similar to the base-case, we evaluated health outcomes, costs and antibiotic overuse outcomes in bacterial-endemic setting (Scenario A) and viral-endemic setting (Scenario B) in Thailand. The per-patient costs incurred (in USD), health burden (in DALY), and antibiotic overuse ($Prob(over)$), underuse ($Prob(under)$) for each strategy are shown in S9 Table for patients seeking care on the first, fourth, and tenth day of illness respectively. In addition, Figures S13 – S15 Fig show the optimal policy (highest NMB) at varying values of WTP and antibiotic overuse penalty ($w_{over}$) for patients presenting on day one of illness (S13 Fig), day four of illness (S14 Fig) and day ten of illness (S15 Fig).

In both settings, the base-case Pareto-efficient strategies remained on the effectiveness frontier, and the optimal strategy sequence when fixing WTP to Thailand GDP per capita did not change. In bacterial-endemic Scenario A, Strategy 17 (S: Dengue PCR, lepto RDT) was added onto the frontier for day one and day four; and Strategy 19 (S: Dengue RDT, lepto RDT) was added onto the frontier for day ten. From Figures S13 – S15 Fig, we observe that both Strategy 17 and Strategy 19 were only optimal (in NMB) for WTP between $0 to $250.

In viral-endemic Scenario B, however, on day one and day four, all four new strategies were added onto the frontier. On the tenth day of illness, Strategy 19 (S: Dengue RDT, lepto RDT) was added onto the frontier. From Figures S13 – S14 Fig, we observe that Strategy 16 and Strategy 17 (both starts with a dengue PCR test) were optimal at low WTP (between $0 to $4000), high penalty region (the lower-right corner). On the other hand, Strategy 18 and 19 (both started with a dengue RDT test) were only optimal for a very small region (low WTP and low penalty) at the lower-left corner. These results were mainly driven by two-fold reasons. First, when patients showed up in hospitals early (i.e., less than their fifth days of illness), dengue PCR test was more accurate than dengue RDT test. As a consequence, strategies having dengue PCR tests (Strategy 5 and Strategy 15, 16) were optimal for a wide range of WTPs and penalties whereas strategies having dengue RDT tests strategy were either not optimal (e.g., Strategy 4), or optimal only for a very small range of WTPs and penalties (e.g., Strategy 17, 18). Second, since leptospirosis was the most frequent bacterial infections, it is beneficial to have a follow-up leptospirosis test. On the tenth day of illness, only Strategy 19 was added onto the frontier (S15 Fig). This is because on the tenth day of illness, RDT for both leptospirosis and dengue dominated the corresponding PCR tests in both test sensitivity and specificity, thus strategies with PCR components were not on the effectiveness frontier.

**S1 Table: Values for model variables**

| **Variable** | **Base Value** | **Range** | | **Source** |
| --- | --- | --- | --- | --- |
|  |  | **Min** | **Max** |  |
| ***Population*** | | | | |
| Cohort Age | 40 | 20 | 60 | assumed |
| Antibiotic effectiveness | 100% | 50% | 100% | [1] |
| ***Daily transition probabilities: Leptospirosis*** | | | | |
| From Mild (with antibiotics) |  |  |  |  |
| to Mild | 0.510 |  |  | [1] |
| to Severe | 0.000 | 0.000 | 0.000 | [1] |
| to Recovered | 0.490 | 0.243 | 1.000 | [1] |
| From Severe (with antibiotics) |  |  |  |  |
| to Severe | 0.833 |  |  | [2, 24] |
| to Recovered | 0.146 | 0.156 | 0.137 | [2, 24] |
| to Dead | 0.021 | 0.011 | 0.029 |  |
| From Mild (without antibiotics) |  |  |  |  |
| to Mild | 0.815 |  |  | [1] |
| to Severe | 0.074 | 0.067 | 0.083 | [1] |
| to Recovered | 0.111 | 0.100 | 0.125 | [1] |
| From Severe (without antibiotics) |  |  |  |  |
| to Severe | 0.929 |  |  | [25] |
| to Recovered | 0.048 | 0.059 | 0.039 | [25] |
| to Dead | 0.023 | 0.012 | 0.033 | [4] |
|  |  |  |  |  |
| ***Tests Parameters, Leptospirosis*** | | | | |
| Rapid Test-costs, $ | 5.7 | 3 | 7 | [1] |
| Sensitivity ($\leq$ 4 day of illness) | 62% | 41% | 79% | [7] |
| Specificity ($\leq$ 4 day of illness) | 98% | 93% | 99% | [7] |
| Sensitivity ($\geq$ 5 day of illness) | 81% | 69% | 90% | [7] |
| Specificity ($\geq$ 5 day of illness) | 93% | 88% | 96% | [7] |
| Turnaround time, TAT (days) | 1 | 0 | 2 | assumed |
| PCR Test costs, $ | 14 | 10 | 20 | assumed |
| Sensitivity ($\leq$ 4 day of illness) | 85.0% | 73% | 97.50% | [11] |
| Specificity ($\leq$ 4 day of illness) | 95.0% | 90% | 100% | [11] |
| Sensitivity ($\geq$ 5 day of illness) | 41.5% | 26.70% | 57.80% | [11] |
| Specificity ($\geq$ 5 day of illness) | 95.0% | 90% | 100% | [11] |
| Turnaround time, TAT (days) | 2 | 0 | 3 | assumed |
| ***Health Costs, Leptospirosis, $*** | | | | |
| doxycycline prescription (one course) | 2.00 | 1 | 3 | [1] |
| Daily costs in Mild | 5.25 | 5 | 10 | [1] |
| Daily costs in Severe | 48.71 | 30 | 70 | [1] |
| ***DALYs, Leptospirosis*** | | | | |
| DW: Mild | 0.21 | 0.15 | 0.3 | [25] |
| DW: Severe | 0.56 | 0.4 | 0.6 | [25] |
| YLL | 34.90 |  |  | [3, 4] |
| ***Daily transition probabilities, other bacterial*** | | | | |
| From Mild (with antibiotics) |  |  |  |  |
| to Mild | 0.625 |  |  | [1] |
| to Severe | 0.000 | 0.000 | 0.000 | [1] |
| to Recovered | 0.375 | 0.063 | 1.000 | [1] |
| From Severe (with antibiotics) |  |  |  |  |
| to Severe | 0.833 |  |  | [2, 24] |
| to Recovered | 0.146 | 0.156 | 0.137 |  |
| to Dead | 0.021 | 0.011 | 0.029 | [2, 24] |
| From Mild (without antibiotics) |  |  |  |  |
| to Mild | 0.811 |  |  | [1] |
| to Severe | 0.075 | 0.051 | 0.148 | [1] |
| to Recovered | 0.113 | 0.076 | 0.222 | [1] |
| From Severe (without antibiotics) |  |  |  |  |
| to Severe | 0.929 |  |  | [26] |
| to Recovered | 0.048 | 0.059 | 0.039 | [26] |
| to Dead | 0.023 | 0.012 | 0.033 | [26] |
| ***Health Costs, other bacterial, $*** | | | | |
| doxycycline prescription (one course) | 2.00 | 1 | 3 | [1] |
| Daily costs in Mild | 5.25 | 5 | 10 | [1] |
| Daily costs in Severe | 48.71 | 30 | 70 | [1] |
| ***DALYs, other bacterial*** |  |  |  |  |
| DW: Mild | 0.2 | 0.15 | 0.3 | [4] |
| DW: Severe | 0.5 | 0.4 | 0.6 | [4] |
| YLL | 34.9 |  |  | [3, 4] |
| ***Daily transition probabilities, typhus*** | | | | |
| From Mild (with antibiotics) |  |  |  |  |
| to Mild | 0.383 |  |  | [1] |
| to Severe | 0.000 | 0.000 | 0.000 | [1] |
| to Recovered | 0.617 | 0.338 | 1.000 | [1] |
| From Severe (with antibiotics) |  |  |  |  |
| to Severe | 0.833 |  |  | [2, 24] |
| to Recovered | 0.146 | 0.156 | 0.137 | [2, 24] |
| to Dead | 0.021 | 0.011 | 0.029 | [2, 24] |
| From Mild (without antibiotics) |  |  |  |  |
| to Mild | 0.811 |  |  | [1] |
| to Severe | 0.075 | 0.076 | 0.222 | [1] |
| to Recovered | 0.113 | 0.051 | 0.148 | [1] |
| From Severe (without antibiotics) |  |  |  |  |
| to Severe | 0.929 |  |  | [26] |
| to Recovered | 0.048 | 0.059 | 0.039 | [26] |
| to Dead | 0.023 | 0.012 | 0.033 | [26] |
| ***Tests parameters, typhus*** | | | | |
| Rapid Test-costs, $ | 5.7 | 3 | 7 | [1] |
| Sensitivity ($\leq$ 4 day of illness) | 67% | 41% | 79% | [8] |
| Specificity ($\leq$ 4 day of illness) | 98% | 93% | 99% | [8] |
| Sensitivity ($\geq$ 5 day of illness) | 80% | 69% | 90% | assumed similar as leptospirosis |
| Specificity ($\geq$ 5 day of illness) | 95% | 88% | 96% | assumed similar as leptospirosis |
| Turnaround time, TAT (days) | 1 | 0 | 2 | assumed |
| PCR Test costs, $ | 14 | 10 | 20 | assumed |
| Sensitivity ($\leq$ 4 day of illness) | 85.0% | 73% | 97.50% | assumed similar to leptospirosis |
| Specificity ($\leq$ 4 day of illness) | 95.0% | 90% | 100% | assumed similar to leptospirosis |
| Sensitivity ($\geq$ 5 day of illness) | 41.5% | 26.70% | 57.80% | assumed similar to leptospirosis |
| Specificity ($\geq$ 5 day of illness) | 95.0% | 90% | 100% | assumed similar to leptospirosis |
| Turnaround time, TAT (days) | 2 | 0 | 3 | assumed |
| ***Health Costs, typhus, $*** | | | | |
| doxycycline prescription (one course) | 2.00 | 1 | 3 | [1] |
| Daily costs in Mild | 5.25 | 5 | 10 | [1] |
| Daily costs in Severe | 48.71 | 30 | 70 | [1] |
| **DALYs, typhus** |  |  |  |  |
| DW: Mild | 0.21 | 0.15 | 0.3 | [4] |
| DW: Severe | 0.56 | 0 | 0.6 | [4] |
| YLL | 34.90 |  |  | [3, 4] |
| ***Daily transition probabilities, dengue*** | | | | |
| From Mild (with standard care) |  |  |  |  |
| to Mild | 0.815 |  |  | [27, 28] |
| to Severe | 0.027 | 0.061 | 0.214 | [27, 28] |
| to Recovered | 0.159 | 0.010 | 0.036 | [27, 28] |
| From Severe |  |  |  |  |
| to Severe | 0.898 |  |  | [29, 30] |
| to Recovered | 0.089 | 0.095 | 0.073 | [29, 30] |
| to Dead | 0.013 | 0.007 | 0.013 | [29, 30] |
| ***Tests Parameters, dengue*** |  |  |  |  |
| Rapid Test-costs, $ | 5.7 | 3 | 7 | assumed similar as lepto |
| Sensitivity ($\leq$ 4 day of illness) | 68% | 60% | 75% | [9, 31] |
| Specificity ($\leq$ 4 day of illness) | 76% | 71% | 80% | [9, 31] |
| Sensitivity ($\geq$ 5 day of illness) | 90% | 80% | 99% | [9, 31] |
| Specificity ($\geq$ 5 day of illness) | 98% | 95% | 100% | [9, 31] |
| Turnaround time, TAT (days) | 1 | 0 | 2 | assumed |
| PCR Test-costs, $ | 14.00 | 10 | 20 | assumed |
| Sensitivity ($\leq$ 4 day of illness) | 85% | 51% | 100% | [10] |
| Specificity ($\leq$ 4 day of illness) | 100% | 100% | 100% | [10] |
| Sensitivity ($\geq$ 5 day of illness) | 50% | 30% | 70% | [10] |
| Specificity ($\geq$ 5 day of illness) | 100% | 100% | 100% | [10] |
| Turnaround time, TAT (days) | 2 | 0 | 3 | assumed |
| ***Health Costs, dengue*** | | | | |
| Daily costs in Mild, $ | 5.25 | 5 | 10 | [32] |
| Daily costs in Severe, $ | 59.68 | 40 | 60 | [32] |
| ***DALY-related, dengue*** | | | | |
| DW: Mild | 0.20 | 0.15 | 0.3 | [5] |
| DW: Severe | 0.56 | 0.4 | 0.6 | [5] |
| YLL | 34.90 |  |  | [3, 4] |
| ***Real-time Multiplex PCR*** | | | | |
| Price per test, $ | 50 | 30 | 80 | assumed |
| Lepto Sensitivity ($\leq$ 4 day of illness) | 90% | 70% | 100% | assumed |
| Lepto Specificity ($\leq$ 4 day of illness) | 95% | 90% | 100% | assumed |
| Typhus Sensitivity ($\leq$ 4 day of illness) | 87% | 74.2% | 94.4% | [12] |
| Typhus Specificity ($\leq$ 4 day of illness) | 100% | 97.3% | 100% | [12] |
| Dengue Sensitivity ($\leq$ 4 day of illness) | 90% | 70% | 100% | assumed |
| Dengue Specificity ($\leq$ 4 day of illness) | 95% | 90% | 100% | assumed |
| Lepto Sensitivity ($\geq$ 5 day of illness) | 60% | 40% | 80% | assumed |
| Lepto Specificity ($\geq$ 5 day of illness) | 90% | 85% | 98% | assumed |
| Typhus Sensitivity ($\geq$ 5 day of illness) | 55% | 40% | 80% | assumed |
| Typhus Specificity ($\geq$ 5 day of illness) | 97% | 85% | 98% | assumed |
| Dengue Sensitivity ($\geq$ 5 day of illness) | 60% | 40% | 80% | assumed |
| Dengue Specificity ($\geq$ 5 day of illness) | 90% | 85% | 98% | assumed |
| Turnaround time, TAT (days) | 2 | 0 | 3 | assumed |

**S2 Table: Results of one-way sensitivity analysis (numbers are the corresponding strategy orders).** We fixed the disease occurrence probabilities to the ones in Scenario A, and set the WTP to Thailand GDP per capita. We assumed that patients presented to hospitals on day four (average day of illness). Strategy 2: Empirical to all; Strategy 5: Dengue PCR; Strategy 9: S: Lepto PCR, typhus RDT; Strategy 13: P: Lepto PCR, typhus RDT; Strategy 15: Multiplex PCR.

| **Variable** | **Parameter Value (min/max)** | **Penalty on antibiotic overuse (in USD)** | | |
| --- | --- | --- | --- | --- |
|  |  | **$0** | **$10,000** | **$50,000** |
| *Base-case optimal strategy sequence* | | 2 | 2 | 15 |
| Cohort Age | 40 (max) | 2 | 15 | 15 |
| Probability from Severe to Death (with antibiotics) | 0.029 (max) | 2 | 15 | 15 |
| Lepto PCR sensitivity | 97.50% (max) | 2 | 2 | 13 |
| Lepto PCR turnaround time (days) | 0 day (min) | 2 | 2 | 9 |
| Dengue PCR turnaround time (days) | 0 day (min) | 2 | 5 | 15 |
| Multiple PCR sensitivity | 70% (min) | 2 | 2 | 13 |
| *All the remaining parameters in S1 Table, either with their min or max values* | | 2 | 2 | 15 |

**S3 Table: Strategy outcomes: per-patient costs and disability-adjusted life years (DALYs) incurred, antibiotic overuse (*Prob(over))* and underuse *(Prob(under))* and for patients seeking care on the first day of illness.**

| Strategies/Scenarios | | *Scenario A: Bacterial-Endemic* | | | | *Scenario B: Viral-Endemic* | | | |
| --- | --- | --- | --- | --- | --- | --- | --- | --- | --- |
|  |  | ***Cost*** | ***DALY*** | ***P(over)*** | ***P(under)*** | ***Cost*** | ***DALY*** | ***P(over)*** | ***P(under)*** |
| 1 | No Antibiotics *º | 216.166 | 2.911 | 0.000 | 0.619 | 138.978 | 1.258 | 0.000 | 0.171 |
| 2 | Empirical All *º | 47.549 | 0.239 | 0.381 | 0.000 | 94.107 | 0.520 | 0.829 | 0.000 |
| 3 | Empirical Severe º | 130.630 | 1.323 | 0.055 | 0.248 | 116.784 | 0.819 | 0.119 | 0.068 |
| 4 | Dengue RDT | 104.111 | 1.034 | 0.198 | 0.132 | 112.024 | 0.739 | 0.282 | 0.036 |
| 5 | Dengue PCR *º | 85.512 | 0.628 | 0.197 | 0.000 | 110.094 | 0.628 | 0.184 | 0.000 |
| 6 | Lepto RDT *º | 137.518 | 1.576 | 0.006 | 0.253 | 132.471 | 1.078 | 0.014 | 0.112 |
| 7 | Lepto PCR * | 122.619 | 1.239 | 0.014 | 0.131 | 134.053 | 1.025 | 0.029 | 0.085 |
| 8 | S: Lepto RDT, typhus RDT º | 129.498 | 1.462 | 0.011 | 0.430 | 129.077 | 1.036 | 0.024 | 0.192 |
| 9 | S: Lepto PCR, typhus RDT *º | 119.643 | 1.142 | 0.017 | 0.216 | 134.098 | 0.988 | 0.037 | 0.146 |
| 10 | S: Lepto RDT, typhus PCR | 127.176 | 1.440 | 0.014 | 0.387 | 128.716 | 1.027 | 0.031 | 0.173 |
| 11 | P: Lepto PCR, typhus PCR | 131.534 | 1.079 | 0.026 | 0.097 | 148.671 | 0.958 | 0.057 | 0.071 |
| 12 | P: Lepto RDT, typhus RDT | 135.543 | 1.437 | 0.013 | 0.222 | 135.924 | 1.025 | 0.028 | 0.100 |
| 13 | P: Lepto PCR, typhus RDT | 125.833 | 1.122 | 0.019 | 0.106 | 141.617 | 0.979 | 0.041 | 0.075 |
| 14 | P: Lepto RDT, typhus PCR | 148.592 | 1.492 | 0.019 | 0.186 | 144.128 | 1.020 | 0.041 | 0.084 |
| 15 | Multiplex PCR*º | 148.696 | 1.001 | 0.014 | 0.080 | 170.800 | 0.961 | 0.029 | 0.072 |

For each scenario, we identified the strategies that were on the three-dimensional effectiveness frontier, where the three dimensions are DALY, cost and antibiotic overuse. * = strategies on the effectiveness frontier (economically efficient) for Scenario A (bacterial-endemic); º = strategies on the effectiveness frontier (economically efficient) for Scenario B (viral-endemic).

**S4 Table: Strategy outcomes: per-patient costs and disability-adjusted life years (DALYs) incurred, antibiotic overuse (*Prob(over)*) and underuse (*Prob(under)*) and for patients seeking care on the tenth day of illness.**

| Strategies/Scenarios | | *Scenario A: Bacterial-Endemic* | | | | *Scenario B: Viral-Endemic* | | | |
| --- | --- | --- | --- | --- | --- | --- | --- | --- | --- |
|  |  | ***Cost*** | ***DALY*** | ***P(over)*** | ***P(under)*** | ***Cost*** | ***DALY*** | ***P(over)*** | ***P(under)*** |
| 1 | No Antibiotics *º | 216.166 | 2.911 | 0.000 | 0.220 | 138.978 | 1.258 | 0.000 | 0.060 |
| 2 | Empirical All *º | 149.508 | 1.720 | 0.075 | 0.000 | 122.128 | 0.930 | 0.163 | 0.000 |
| 3 | Empirical Severe º | 160.095 | 1.857 | 0.055 | 0.248 | 124.963 | 0.967 | 0.119 | 0.068 |
| 4 | Dengue RDT | 155.717 | 1.838 | 0.045 | 0.004 | 122.864 | 0.962 | 0.038 | 0.001 |
| 5 | Dengue PCR *º | 160.426 | 1.906 | 0.047 | 0.000 | 124.658 | 0.981 | 0.072 | 0.000 |
| 6 | Lepto RDT *º | 172.964 | 2.142 | 0.005 | 0.060 | 133.316 | 1.147 | 0.010 | 0.035 |
| 7 | Lepto PCR * | 196.730 | 2.548 | 0.003 | 0.117 | 137.110 | 1.203 | 0.006 | 0.041 |
| 8 | S: Lepto RDT, typhus RDT º | 172.399 | 2.084 | 0.007 | 0.099 | 134.200 | 1.124 | 0.015 | 0.060 |
| 9 | S: Lepto PCR, typhus RDT *º | 201.927 | 2.483 | 0.005 | 0.203 | 145.614 | 1.181 | 0.010 | 0.070 |
| 10 | S: Lepto RDT, typhus PCR | 172.814 | 2.118 | 0.006 | 0.096 | 133.345 | 1.138 | 0.014 | 0.057 |
| 11 | P: Lepto PCR, typhus PCR | 220.263 | 2.490 | 0.005 | 0.107 | 162.760 | 1.183 | 0.012 | 0.037 |
| 12 | P: Lepto RDT, typhus RDT | 179.633 | 2.070 | 0.008 | 0.046 | 142.403 | 1.117 | 0.016 | 0.029 |
| 13 | P: Lepto PCR, typhus RDT | 210.424 | 2.463 | 0.005 | 0.102 | 153.892 | 1.173 | 0.012 | 0.035 |
| 14 | P: Lepto RDT, typhus PCR | 193.554 | 2.165 | 0.006 | 0.047 | 151.943 | 1.138 | 0.014 | 0.029 |
| 15 | Multiplex PCR*º | 233.338 | 2.332 | 0.007 | 0.078 | 183.249 | 1.156 | 0.015 | 0.032 |

For each scenario, we identified the strategies that were on the three-dimensional effectiveness frontier, where the three dimensions are DALY, cost and antibiotic overuse. * = strategies on the effectiveness frontier (economically efficient) for Scenario A (bacterial-endemic); º = strategies on the effectiveness frontier (economically efficient) for Scenario B (viral-endemic).

**S5 Table: Sensitivity analysis of disease occurrence probabilities** Values obtained from [6]

| *Probability of infections* | *leptospirosis* | *typhus* | *Other bacterial* | *dengue* | *other* |
| --- | --- | --- | --- | --- | --- |
| SEt 1 | 0.358 | 0.209 | 0.047 | 0.071 | 0.315 |
| SET 2 | 0.076 | 0.204 | 0.022 | 0.222 | 0.476 |

**S6 Table: Per-patient costs (USD), DALYs incurred, antibiotic overuse (*Prob(over)*) and underuse (*Prob(under)*) for febrile patients seeking care on the fourth day (average day) of illness and undergoing various test and treat strategies, with 75% antibiotic effectiveness.**

| Strategies | | *Scenario A: Bacterial-Endemic* | | | | *Scenario B: Viral-Endemic* | | | |
| --- | --- | --- | --- | --- | --- | --- | --- | --- | --- |
|  |  | ***Cost*** | ***DALY*** | ***P(over)*** | ***P(under)*** | ***Cost*** | ***DALY*** | ***P(over)*** | ***P(under)*** |
| 1 | No Antibiotics *º | 216.166 | 2.911 | 0.000 | 0.394 | 138.978 | 1.258 | 0.000 | 0.109 |
| 2 | Empirical All *º | 107.943 | 1.074 | 0.193 | 0.000 | 110.656 | 0.750 | 0.421 | 0.000 |
| 3 | Empirical Severe | 137.058 | 1.440 | 0.055 | 0.248 | 118.573 | 0.851 | 0.119 | 0.068 |
| 4 | Dengue RDT | 141.889 | 1.614 | 0.102 | 0.085 | 120.261 | 0.900 | 0.144 | 0.023 |
| 5 | Dengue PCR º | 127.833 | 1.327 | 0.102 | 0.000 | 117.781 | 0.821 | 0.095 | 0.000 |
| 6 | Lepto RDT *º | 163.998 | 1.992 | 0.003 | 0.163 | 133.758 | 1.134 | 0.007 | 0.072 |
| 7 | Lepto PCR * | 152.617 | 1.754 | 0.007 | 0.086 | 133.705 | 1.097 | 0.015 | 0.056 |
| 8 | S: Lepto RDT, typhus RDT º | 159.298 | 1.911 | 0.006 | 0.280 | 132.052 | 1.104 | 0.012 | 0.125 |
| 9 | S: Lepto PCR, typhus RDT *º | 155.984 | 1.685 | 0.009 | 0.143 | 138.665 | 1.071 | 0.019 | 0.096 |
| 10 | S: Lepto RDT, typhus PCR º | 156.261 | 1.897 | 0.008 | 0.254 | 130.067 | 1.099 | 0.016 | 0.113 |
| 11 | P: Lepto PCR, typhus PCR | 169.802 | 1.640 | 0.014 | 0.063 | 154.814 | 1.049 | 0.030 | 0.046 |
| 12 | P: Lepto RDT, typhus RDT | 167.390 | 1.892 | 0.007 | 0.143 | 140.858 | 1.096 | 0.014 | 0.064 |
| 13 | P: Lepto PCR, typhus RDT | 163.273 | 1.670 | 0.010 | 0.069 | 147.360 | 1.063 | 0.021 | 0.049 |
| 14 | P: Lepto RDT, typhus PCR | 178.179 | 1.925 | 0.010 | 0.121 | 148.990 | 1.091 | 0.021 | 0.055 |
| 15 | Multiplex PCR*º | 188.618 | 1.585 | 0.007 | 0.052 | 176.905 | 1.051 | 0.015 | 0.047 |

* = strategies on the effectiveness frontier (economically efficient) for Scenario A (bacterial-endemic); º = strategies on the effectiveness frontier (economically efficient) for Scenario B (viral-endemic).

**S7 Table: Per-patient costs (USD), DALYs incurred, antibiotic overuse (*Prob(over)*) and underuse (*Prob(under)*) for febrile patients seeking care on the fourth day (average day) of illness and undergoing various test and treat strategies, with 50% antibiotic effectiveness.**

| Strategies | | *Scenario A: Bacterial-Endemic* | | | | *Scenario B: Viral-Endemic* | | | |
| --- | --- | --- | --- | --- | --- | --- | --- | --- | --- |
|  |  | ***Cost*** | ***DALY*** | ***P(over)*** | ***P(under)*** | ***Cost*** | ***DALY*** | ***P(over)*** | ***P(under)*** |
| 1 | No Antibiotics *º | 216.166 | 2.911 | 0.000 | 0.394 | 138.978 | 1.258 | 0.000 | 0.109 |
| 2 | Empirical All *º | 115.311 | 1.185 | 0.193 | 0.000 | 112.619 | 0.780 | 0.421 | 0.000 |
| 3 | Empirical Severe º | 137.058 | 1.440 | 0.055 | 0.248 | 118.573 | 0.851 | 0.119 | 0.068 |
| 4 | Dengue RDT | 146.449 | 1.682 | 0.102 | 0.085 | 121.474 | 0.918 | 0.144 | 0.023 |
| 5 | Dengue PCR *º | 132.720 | 1.400 | 0.102 | 0.000 | 119.078 | 0.840 | 0.095 | 0.000 |
| 6 | Lepto RDT *º | 167.497 | 2.044 | 0.003 | 0.163 | 134.225 | 1.141 | 0.007 | 0.072 |
| 7 | Lepto PCR * | 156.471 | 1.812 | 0.007 | 0.086 | 134.235 | 1.105 | 0.015 | 0.056 |
| 8 | S: Lepto RDT, typhus RDT º | 162.830 | 1.964 | 0.006 | 0.280 | 132.534 | 1.112 | 0.012 | 0.125 |
| 9 | S: Lepto PCR, typhus RDT *º | 159.851 | 1.743 | 0.009 | 0.143 | 139.205 | 1.079 | 0.019 | 0.096 |
| 10 | S: Lepto RDT, typhus PCR º | 159.814 | 1.950 | 0.008 | 0.254 | 130.558 | 1.106 | 0.016 | 0.113 |
| 11 | P: Lepto PCR, typhus PCR | 173.708 | 1.698 | 0.014 | 0.063 | 155.382 | 1.057 | 0.030 | 0.046 |
| 12 | P: Lepto RDT, typhus RDT | 170.938 | 1.945 | 0.007 | 0.143 | 141.347 | 1.103 | 0.014 | 0.064 |
| 13 | P: Lepto PCR, typhus RDT | 167.147 | 1.728 | 0.010 | 0.069 | 147.905 | 1.072 | 0.021 | 0.049 |
| 14 | P: Lepto RDT, typhus PCR | 181.131 | 1.969 | 0.010 | 0.121 | 149.416 | 1.098 | 0.021 | 0.055 |
| 15 | Multiplex PCR*º | 192.698 | 1.647 | 0.007 | 0.052 | 177.463 | 1.059 | 0.015 | 0.047 |

* = strategies on the effectiveness frontier (economically efficient) for Scenario A (bacterial-endemic); º = strategies on the effectiveness frontier (economically efficient) for Scenario B (viral-endemic).

|  | ***Strategy*** | ***Test interpretation treatment decision*** |
| --- | --- | --- |
| 16 | S: Dengue PCR, lepto PCR | Dengue PCR positive: out(in)patient care w/o antibiotics  Dengue PCR negative: perform Lepto PCR  Lepto PCR positive: antibiotic  Lepto PCR negative: out(in)patient care w/o antibiotics |
| 17 | S: Dengue PCR, lepto RDT | Dengue PCR positive: out(in)patient care w/o antibiotics  Dengue PCR negative: perform Lepto RDT  Lepto RDT positive: antibiotic  Lepto RDT negative: out(in)patient care w/o antibiotics |
| 18 | S: Dengue RDT, lepto PCR | Dengue RDT positive: out(in)patient care w/o antibiotics  Dengue RDT negative: perform Lepto PCR  Lepto PCR positive: antibiotic  Lepto PCR negative: out(in)patient care w/o antibiotics |
| 19 | S: Dengue RDT, lepto RDT | Dengue RDT positive: out(in)patient care w/o antibiotics  Dengue RDT negative: perform Lepto RDT  Lepto RDT positive: antibiotic  Lepto RDT negative: out(in)patient care w/o antibiotics |

**S8 Table: Additional Strategies evaluated for two Thai settings using Markov cohort models**

**S9 Table: Per-patient costs (USD), DALYs incurred, antibiotic overuse (*Prob(over)*) and underuse (*Prob(under)*) for febrile patients on different days of presentation and undergoing various test and treat strategies.**

| Strategies | | *Scenario A: Bacterial-Endemic* | | | | *Scenario B: Viral-Endemic* | | | |
| --- | --- | --- | --- | --- | --- | --- | --- | --- | --- |
|  |  | ***Cost*** | ***DALY*** | ***P(over)*** | ***P(under)*** | ***Cost*** | ***DALY*** | ***P(over)*** | ***P(under)*** |
| First (min) day of illness | | | | | | | | | |
| 16 | S: Dengue PCR, lepto PCR º | 150.216 | 1.702 | 0.005 | 0.086 | 140.603 | 1.093 | 0.005 | 0.056 |
| 17 | S: Dengue PCR, lepto RDT *º | 162.273 | 1.864 | 0.003 | 0.181 | 142.908 | 1.119 | 0.003 | 0.080 |
| 18 | S: Dengue RDT, lepto PCR º | 157.232 | 1.915 | 0.005 | 0.204 | 135.054 | 1.122 | 0.007 | 0.083 |
| 19 | S: Dengue RDT, lepto RDT º | 166.438 | 2.049 | 0.003 | 0.285 | 136.533 | 1.143 | 0.004 | 0.104 |
| Fourth (average) day of illness | | | | | | | | | |
| 16 | S: Dengue PCR, lepto PCR º | 169.362 | 2.044 | 0.003 | 0.058 | 142.491 | 1.141 | 0.003 | 0.038 |
| 17 | S: Dengue PCR, lepto RDT *º | 179.805 | 2.155 | 0.001 | 0.121 | 144.863 | 1.158 | 0.001 | 0.053 |
| 18 | S: Dengue RDT, lepto PCR º | 172.103 | 2.194 | 0.003 | 0.134 | 135.707 | 1.161 | 0.004 | 0.055 |
| 19 | S: Dengue RDT, lepto RDT º | 180.463 | 2.287 | 0.001 | 0.187 | 137.694 | 1.175 | 0.002 | 0.068 |
| Tenth (max) day of illness | | | | | | | | | |
| 16 | S: Dengue PCR, lepto PCR | 207.182 | 2.670 | 0.001 | 0.084 | 147.064 | 1.225 | 0.002 | 0.029 |
| 17 | S: Dengue PCR, lepto RDT | 191.320 | 2.322 | 0.002 | 0.046 | 145.790 | 1.175 | 0.004 | 0.027 |
| 18 | S: Dengue RDT, lepto PCR | 197.713 | 2.652 | 0.001 | 0.093 | 139.508 | 1.222 | 0.001 | 0.032 |
| 19 | S: Dengue RDT, lepto RDT*º | 180.539 | 2.280 | 0.002 | 0.053 | 137.739 | 1.169 | 0.002 | 0.030 |

* = strategies on the effectiveness frontier (economically efficient) for Scenario A (bacterial-endemic); º = strategies on the effectiveness frontier (economically efficient) for Scenario B (viral-endemic).

**S1 Fig: Patient Flow Diagram**


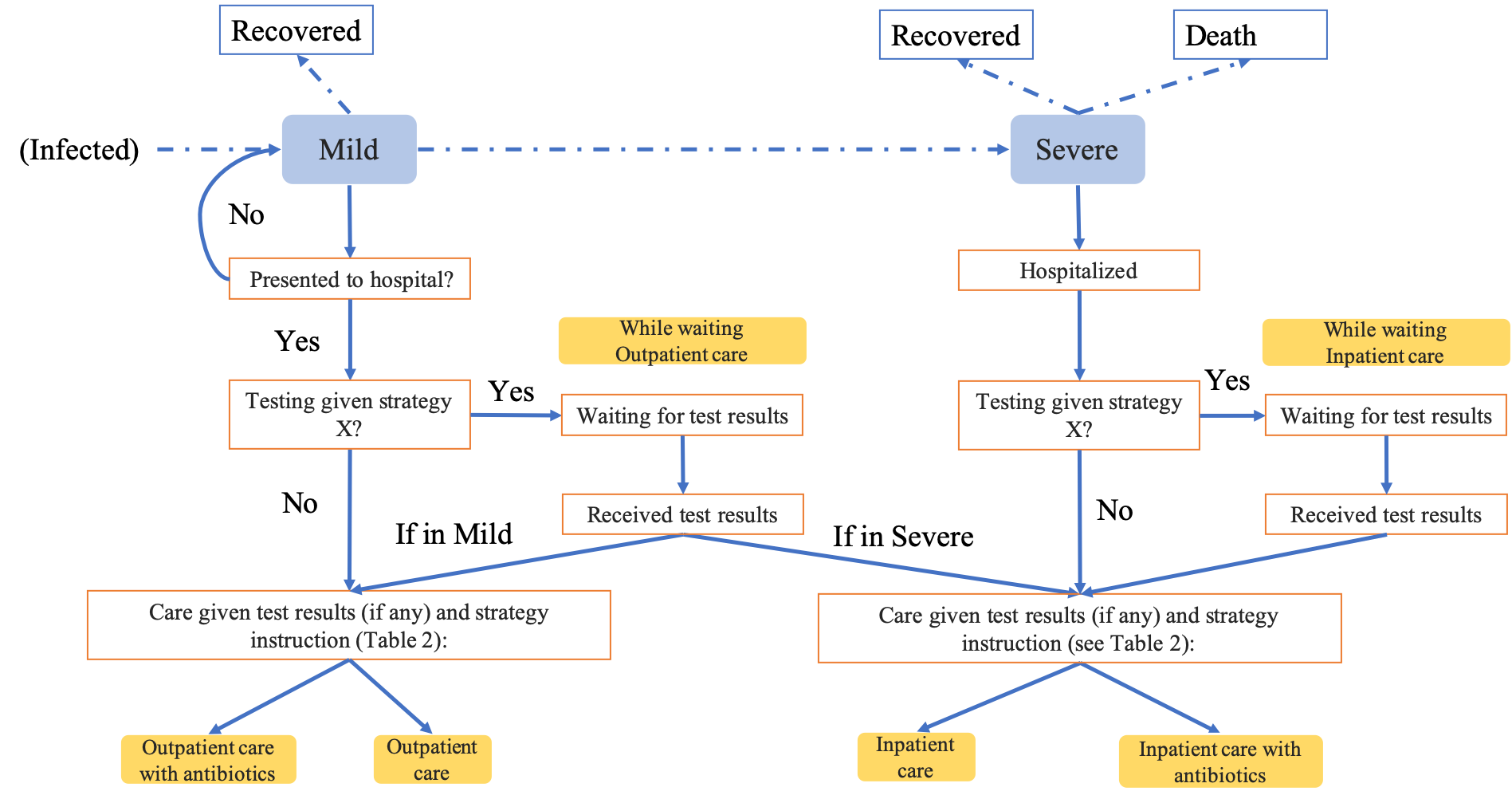


**S2 Fig: Sample Markov trace of disease progression for leptospirosis.** Patients enter the model in Mild state, during the 45-day horizon, they could progress to Severe state, become recovered, or dead.


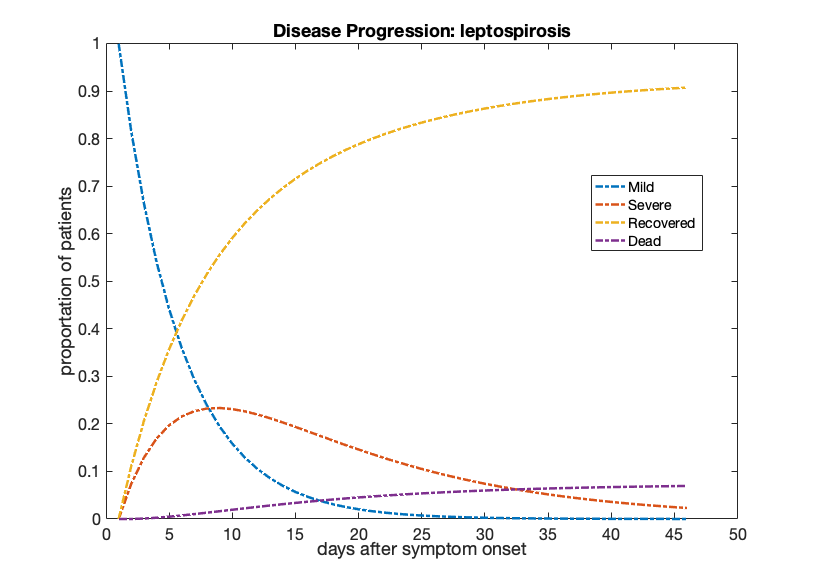


**S3 Fig: Policies with highest NMB with by varying WTP (y-axis) and** $\mathbf{w}_{\mathbf{over}}$ **(x-axis).** We fixed disease occurrence probability vector as Set 1 in S5 Table. NMB = net monetary benefit, WTP = willingness-to-pay, w_over_ = antibiotic overuse penalty

**
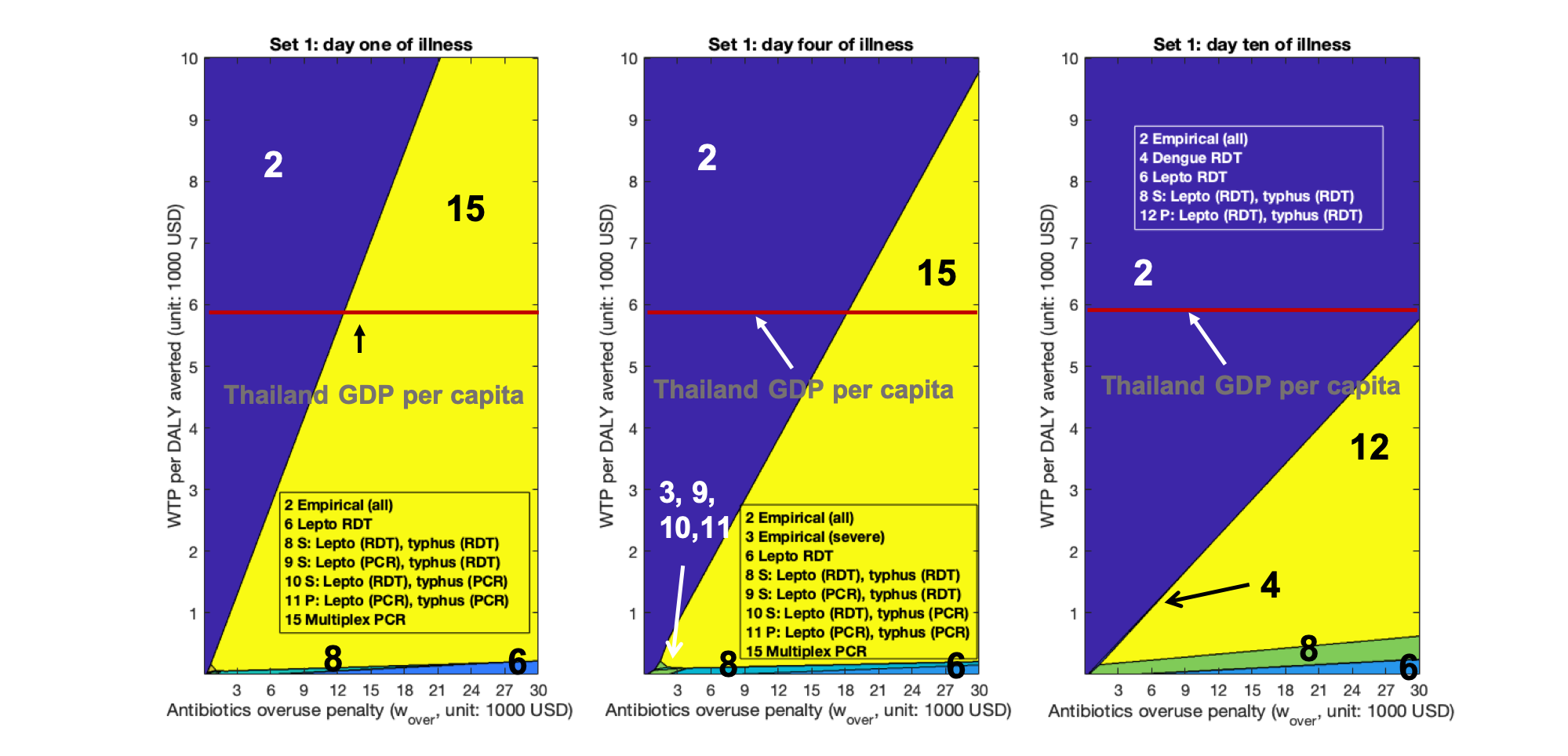
**

**S4 Fig: Policies with highest NMB with by varying WTP (y-axis) and** $\mathbf{w}_{\mathbf{over}}$ **(x-axis).** We fixed disease occurrence probability as Set 2 in S5 Table. NMB = net monetary benefit, WTP = willingness-to-pay, w_over_ = antibiotic overuse penalty


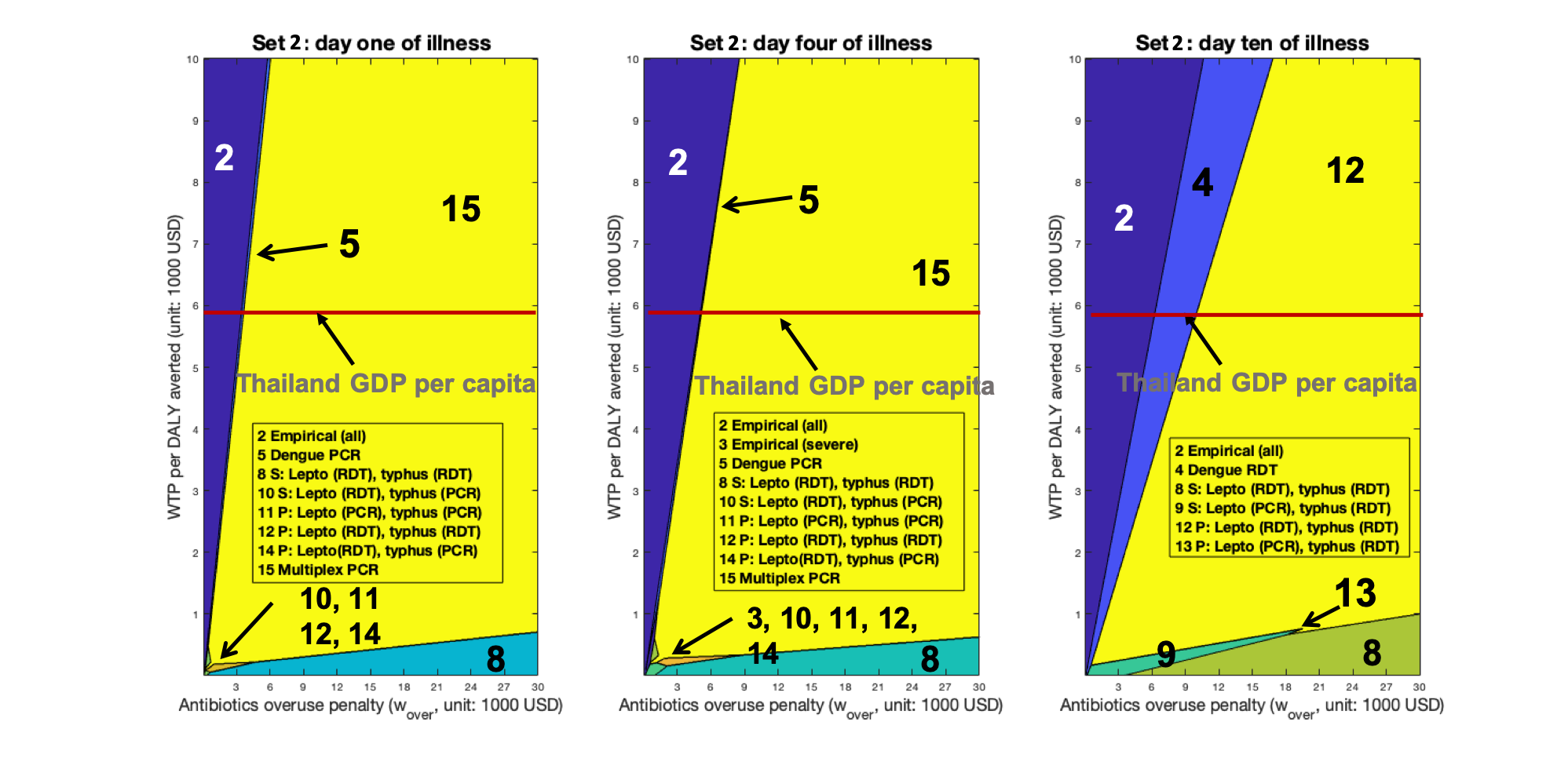


**S5 Fig: Tornado diagram with variation in selected model parameters.** One-way sensitivity analysis, each row (bar) displays the range of Augmented ICER between the empirical antibiotic to all strategy and the Multiplex PCR strategy (patients present to a hospital on day one). We only displayed leptospirosis-specific disease parameters, but all other disease categories share the same structure in the range of ICER change. Augmented ICER = augmented incremental cost-effectiveness ratio, calculated by the ratio of DALY difference and augmented cost difference. Augmented cost = cost + penalty * $w_{\mathrm{over}}$; DALY = disability-adjusted life year; PCR = Polymerase Chain Reaction tests.


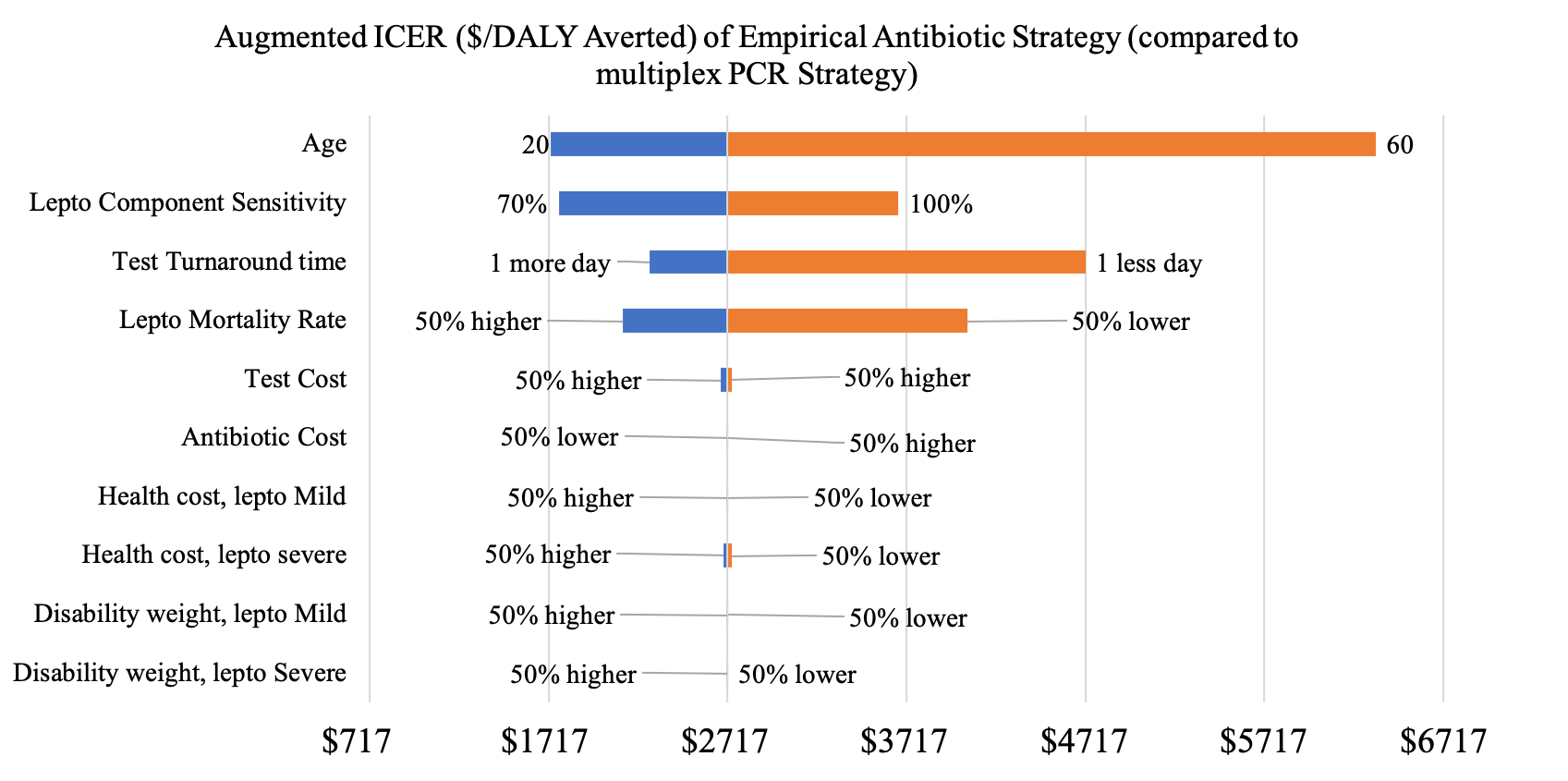


**S6 Fig: Results of probabilistic sensitivity analysis.** We fixed WTP = Thailand GDP per capita. The optimal strategy for a given penalty, is the strategy with the highest NMB value.

**
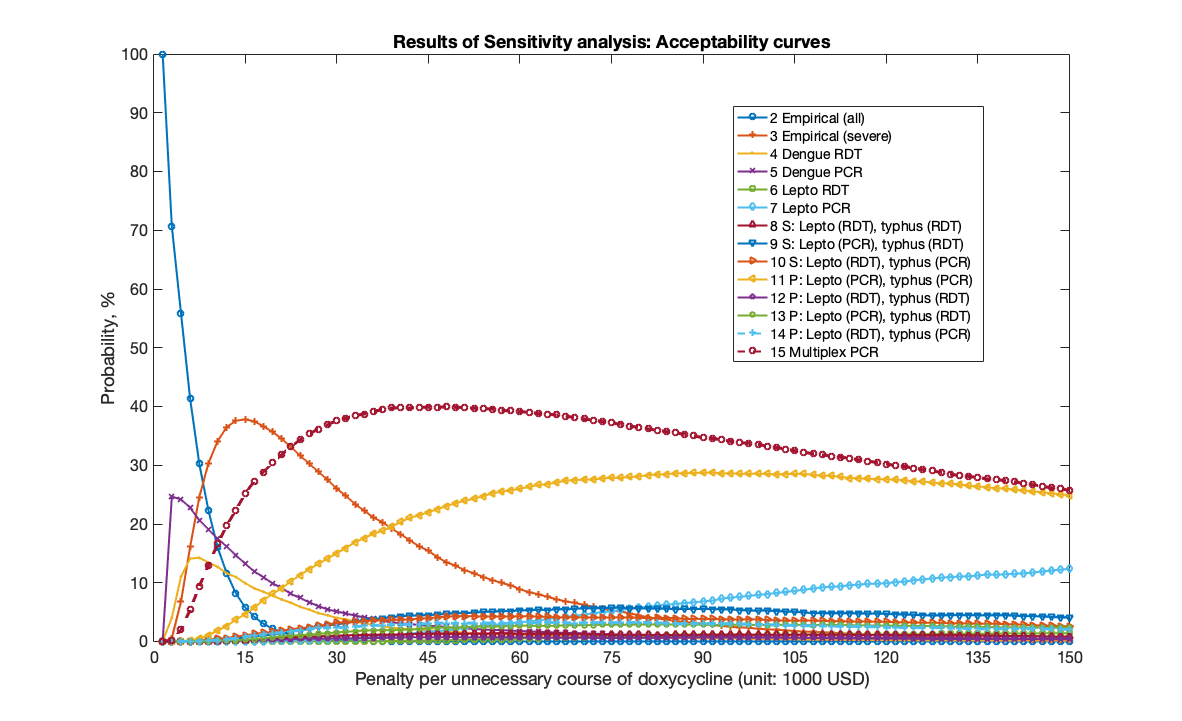
**

**S7 Fig: Highest net monetary benefit policies for patients presenting on the first day of illness in bacterial-endemic Scenario A.** We vary willingness-to-pay (WTP) on the y-axis and penalty ($w_{\mathrm{over}}$) on the x-axis. (A): 100% antibiotic effectiveness (B): 75% antibiotic effectiveness (C): 50% antibiotic effectiveness


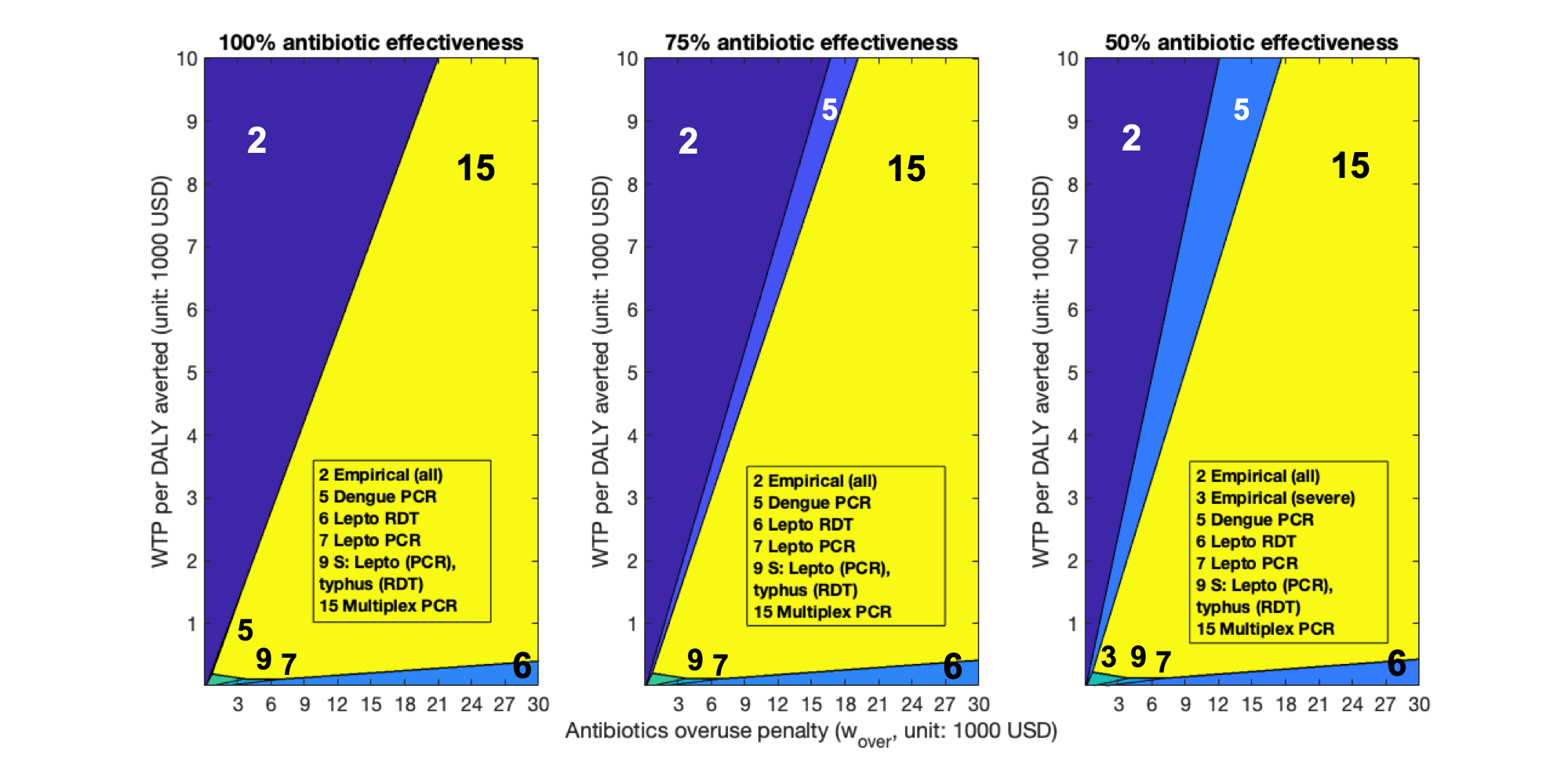


**S8 Fig: Highest net monetary benefit policies for patients presenting on the fourth day of illness in bacterial-endemic Scenario A.** We vary willingness-to-pay (WTP) on the y-axis and penalty ($w_{\mathrm{over}}$) on the x-axis. (A): 100% antibiotic effectiveness (B): 75% antibiotic effectiveness (C): 50% antibiotic effectiveness


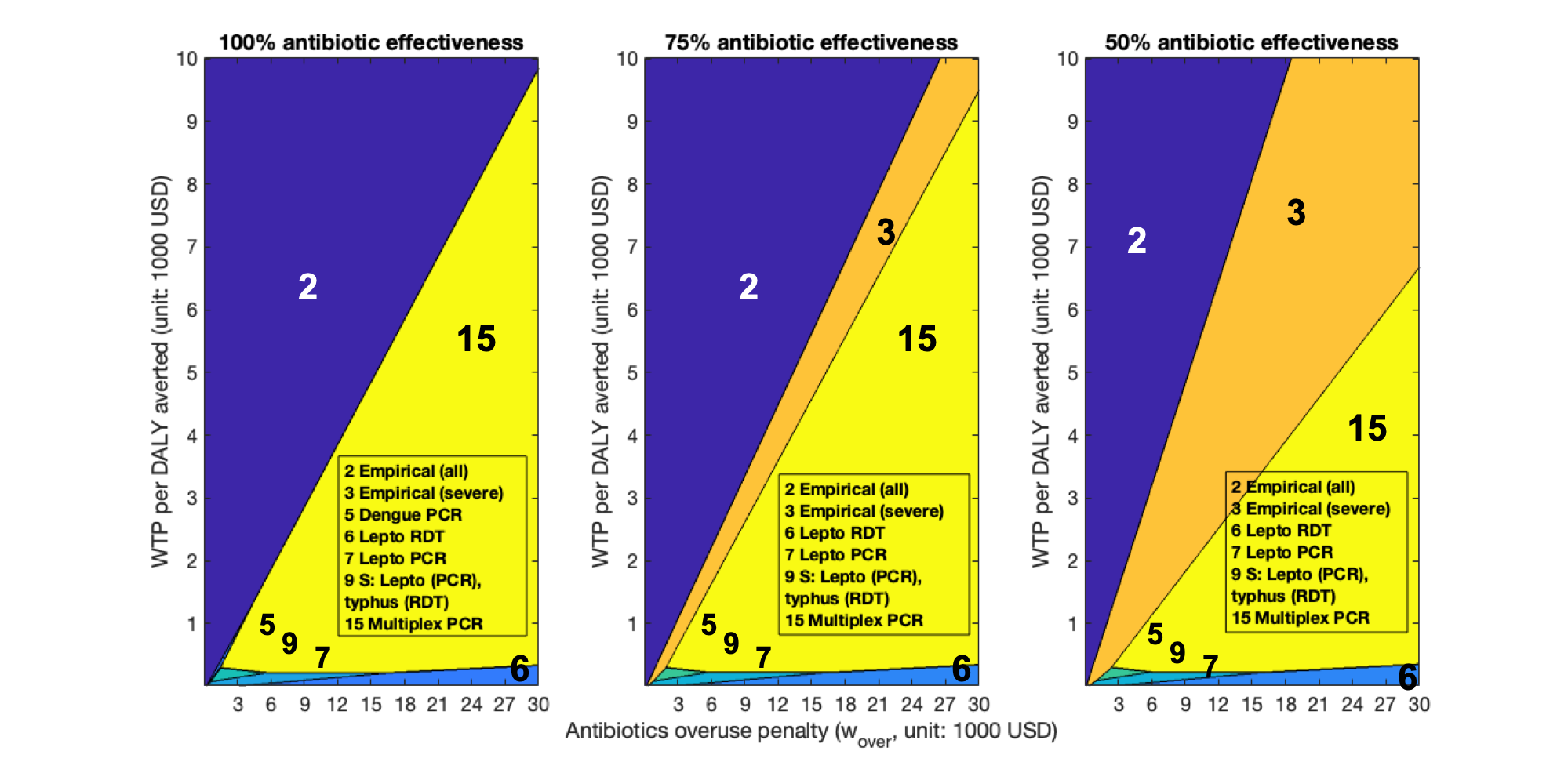


**S9 Fig: Highest net monetary benefit policies for patients presenting on the tenth day of illness in bacterial-endemic Scenario A.** We vary willingness-to-pay (WTP) on the y-axis and penalty ($w_{\mathrm{over}}$) on the x-axis. (A): 100% antibiotic effectiveness (B): 75% antibiotic effectiveness (C): 50% antibiotic effectiveness


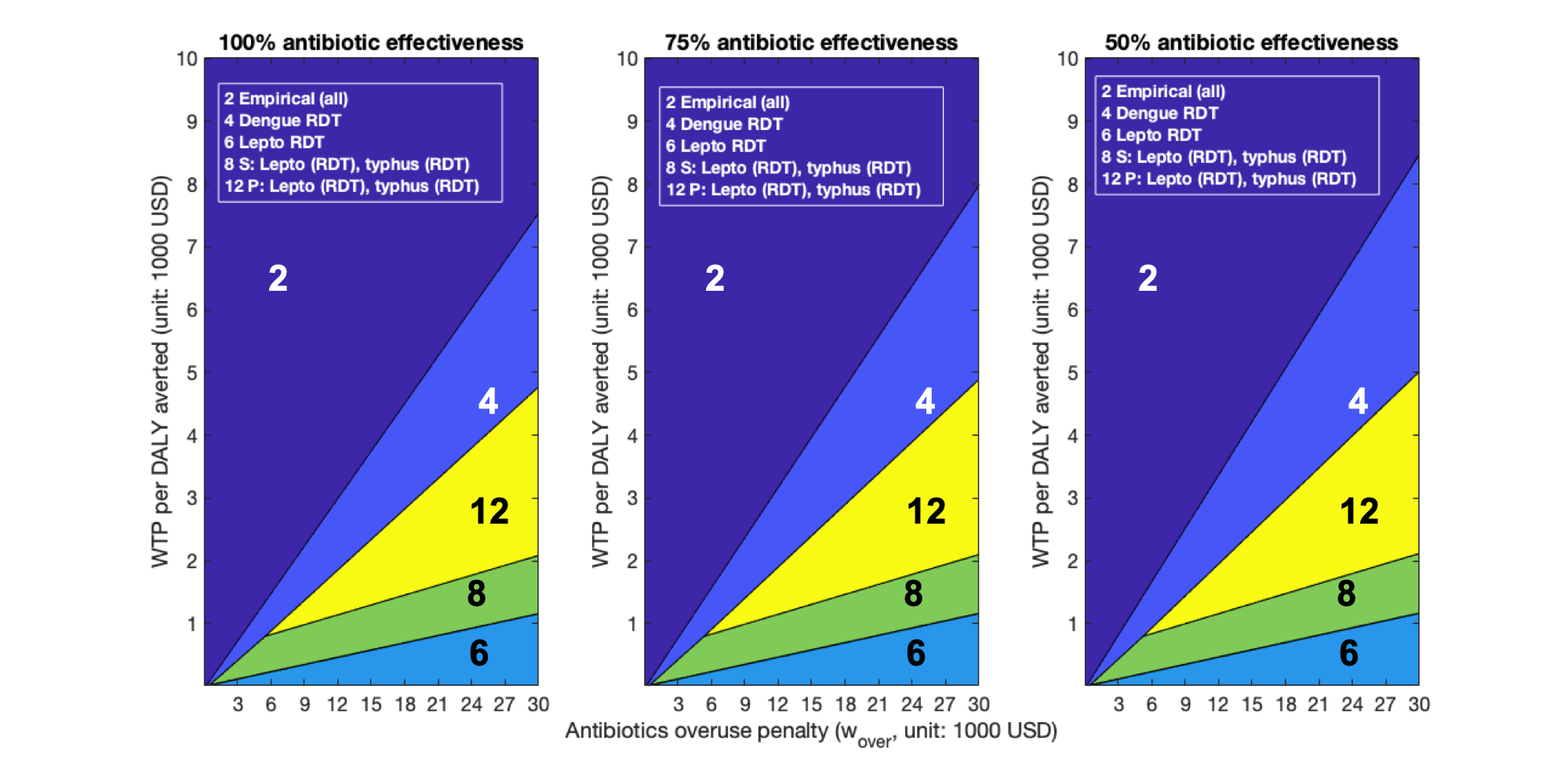


**S10 Fig: Highest net monetary benefit policies for patients presenting on the first day of illness in viral-endemic Scenario B.** We vary willingness-to-pay (WTP) on the y-axis and penalty ($w_{\mathrm{over}}$) on the x-axis. (A): 100% antibiotic effectiveness (B): 75% antibiotic effectiveness (C): 50% antibiotic effectiveness


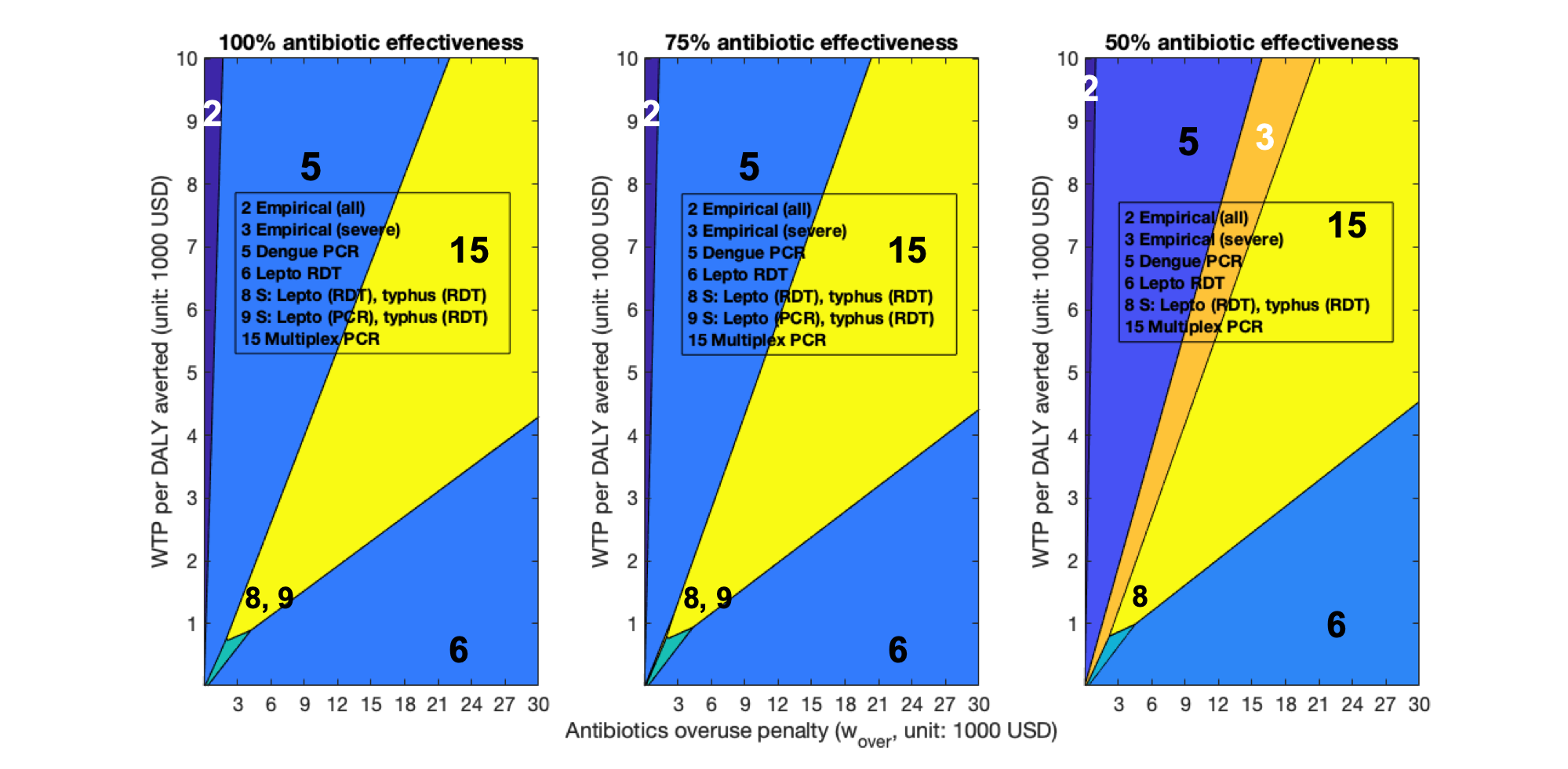


**S11 Fig: Highest net monetary benefit policies for patients presenting on the fourth day of illness in viral-endemic Scenario B.** We vary willingness-to-pay (WTP) on the y-axis and penalty ($w_{\mathrm{over}}$) on the x-axis. (A): 100% antibiotic effectiveness (B): 75% antibiotic effectiveness (C): 50% antibiotic effectiveness


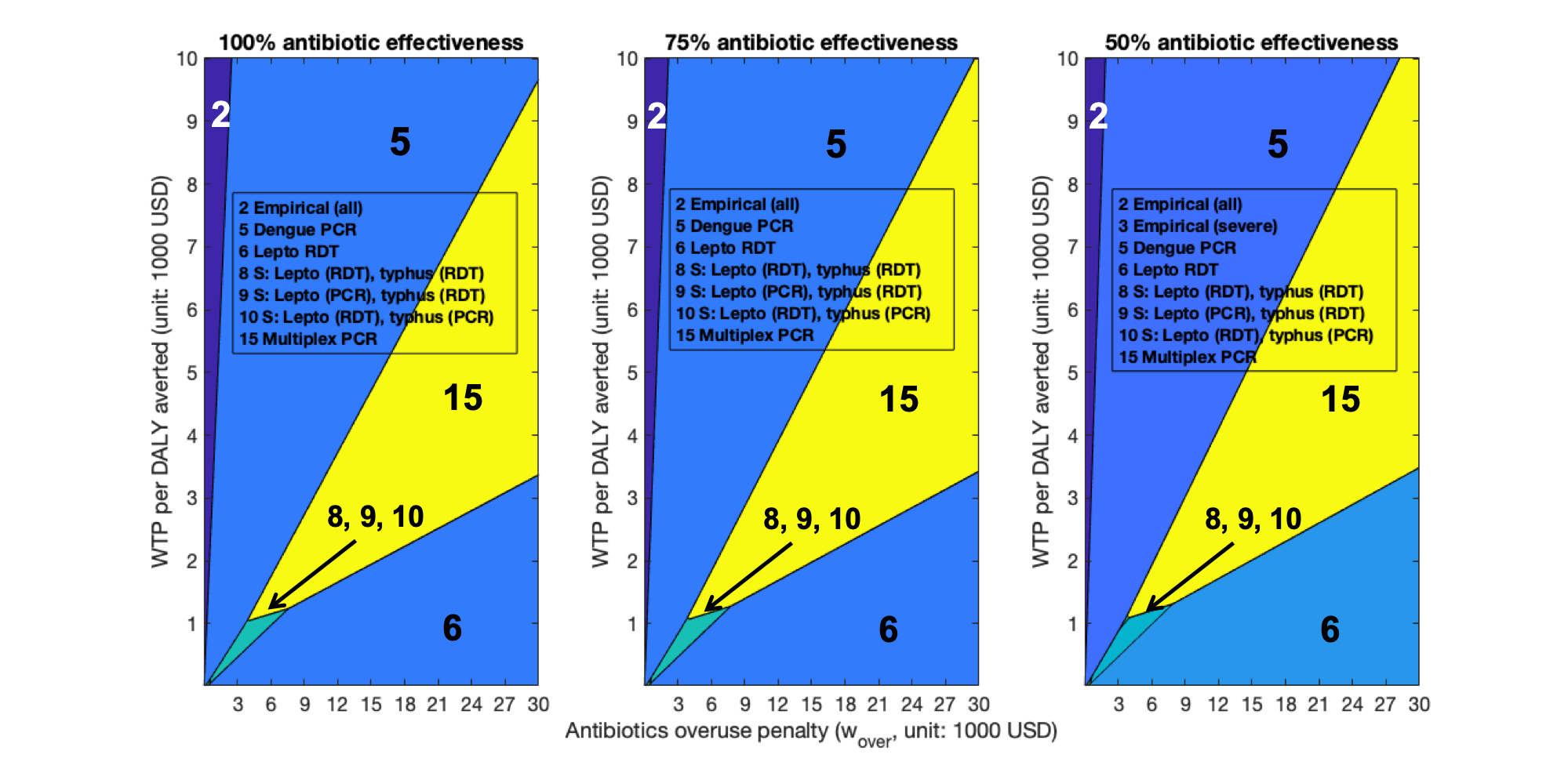
**S12 Fig: Highest net monetary benefit policies for patients presenting on the tenth day of illness in viral-endemic Scenario B.** We vary willingness-to-pay (WTP) on the y-axis and penalty ($w_{\mathrm{over}}$) on the x-axis. (A): 100% antibiotic effectiveness (B): 75% antibiotic effectiveness (C): 50% antibiotic effectiveness
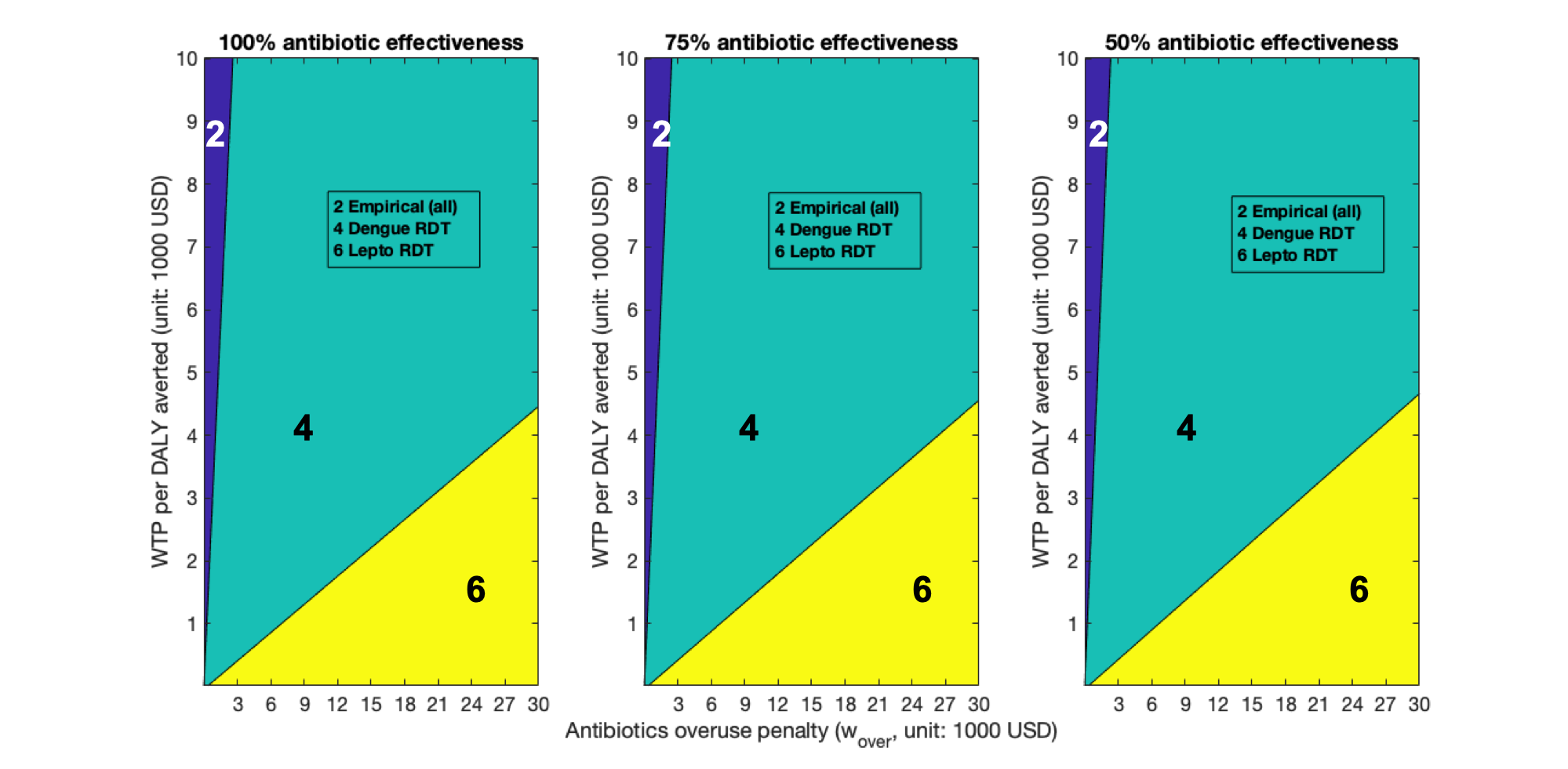


**S13 Fig: Highest net monetary benefit policies for patients presenting on the first day of illness.** We vary willingness-to-pay (WTP) on the y-axis and penalty ($w_{\mathrm{over}}$) on the x-axis. (A): Bacterial-endemic Scenario A (B): Viral-endemic Scenario B

**
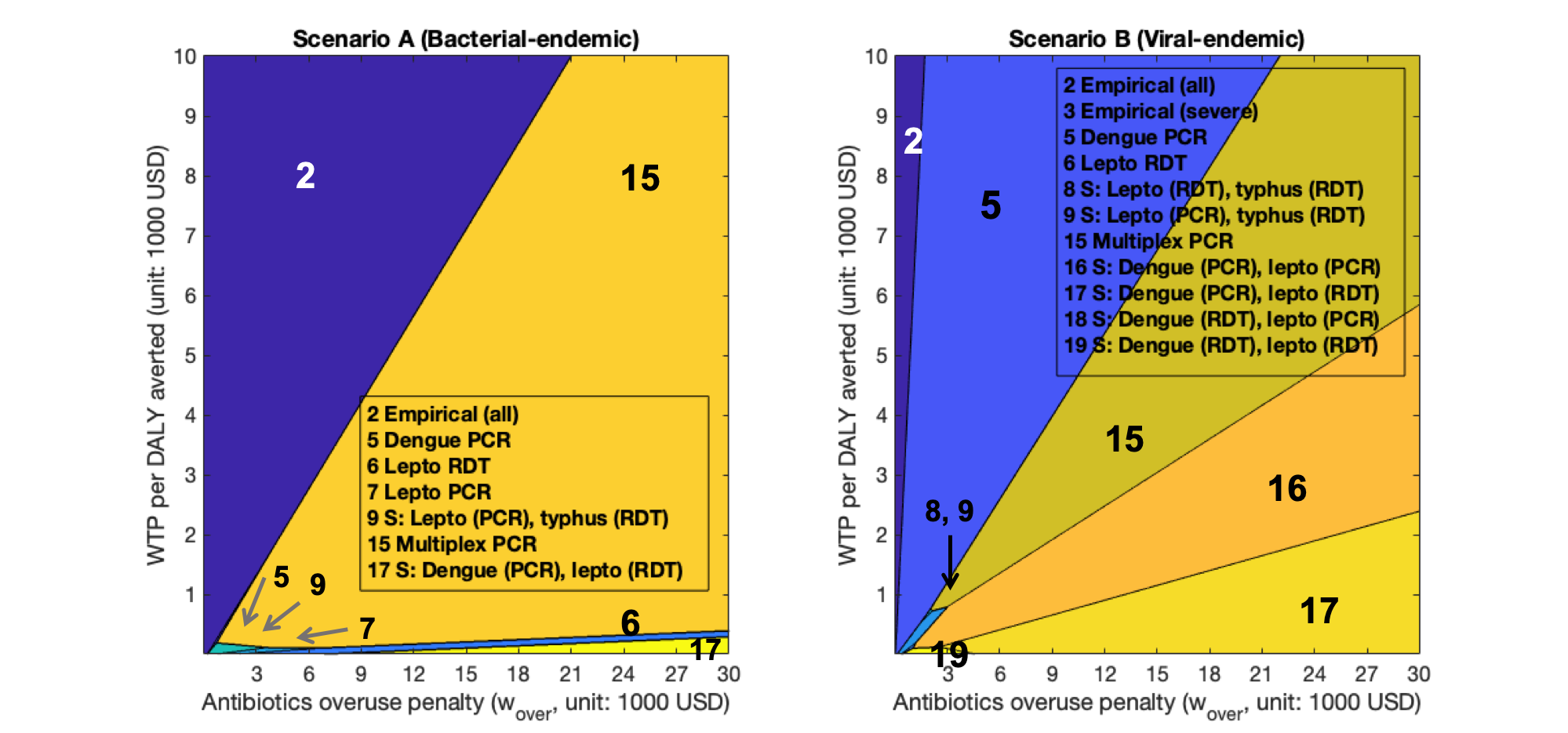
**

**S14 Fig: Highest net monetary benefit policies for patients presenting on the fourth day of illness.** We vary willingness-to-pay (WTP) on the y-axis and penalty ($w_{\mathrm{over}}$) on the x-axis. (A): Bacterial-endemic Scenario A (B): Viral-endemic Scenario B

**
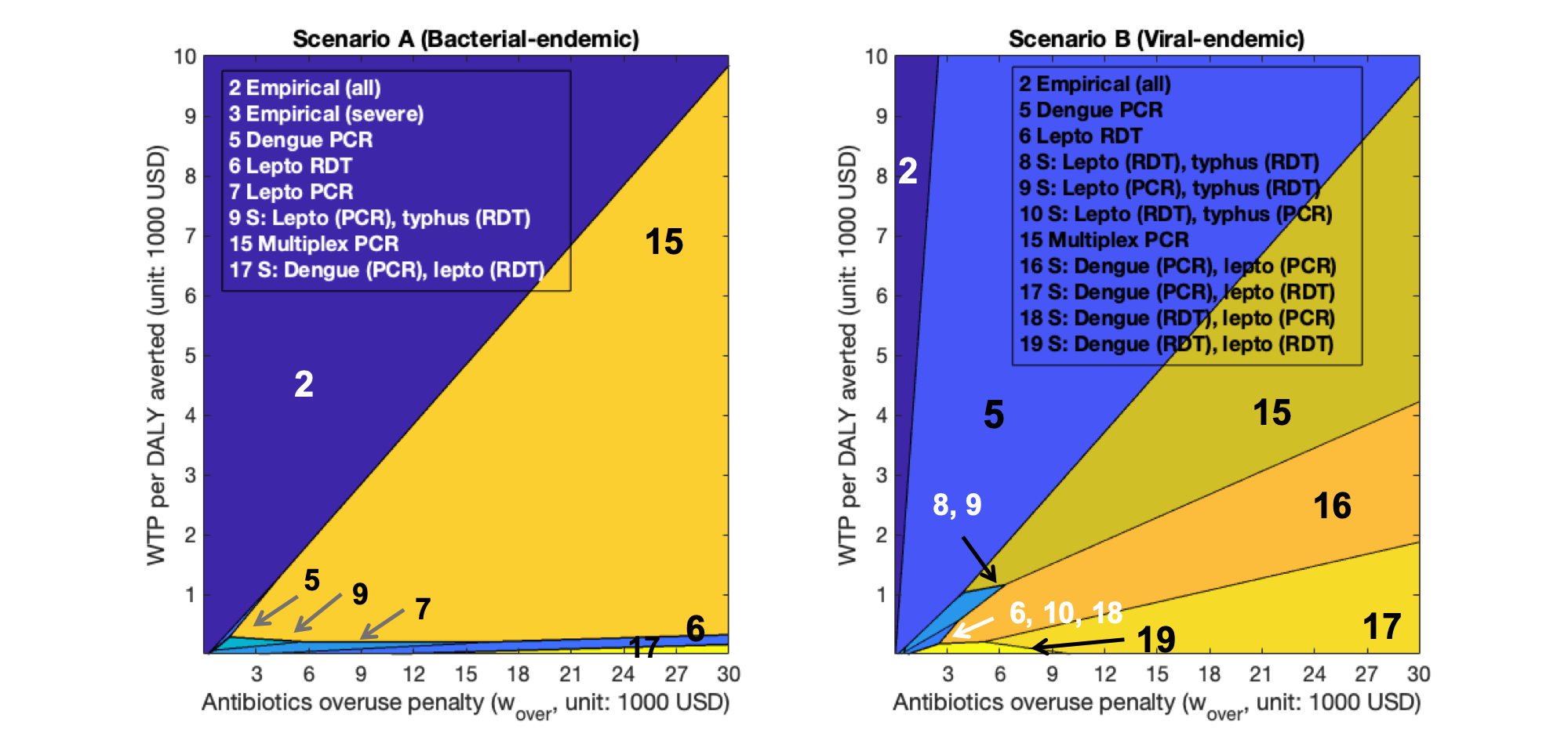
**

**S15 Fig: Highest net monetary benefit policies for patients presenting on the tenth day of illness.** We vary willingness-to-pay (WTP) on the y-axis and penalty ($w_{\mathrm{over}}$) on the x-axis. (A): Bacterial-endemic Scenario A (B): Viral-endemic Scenario B


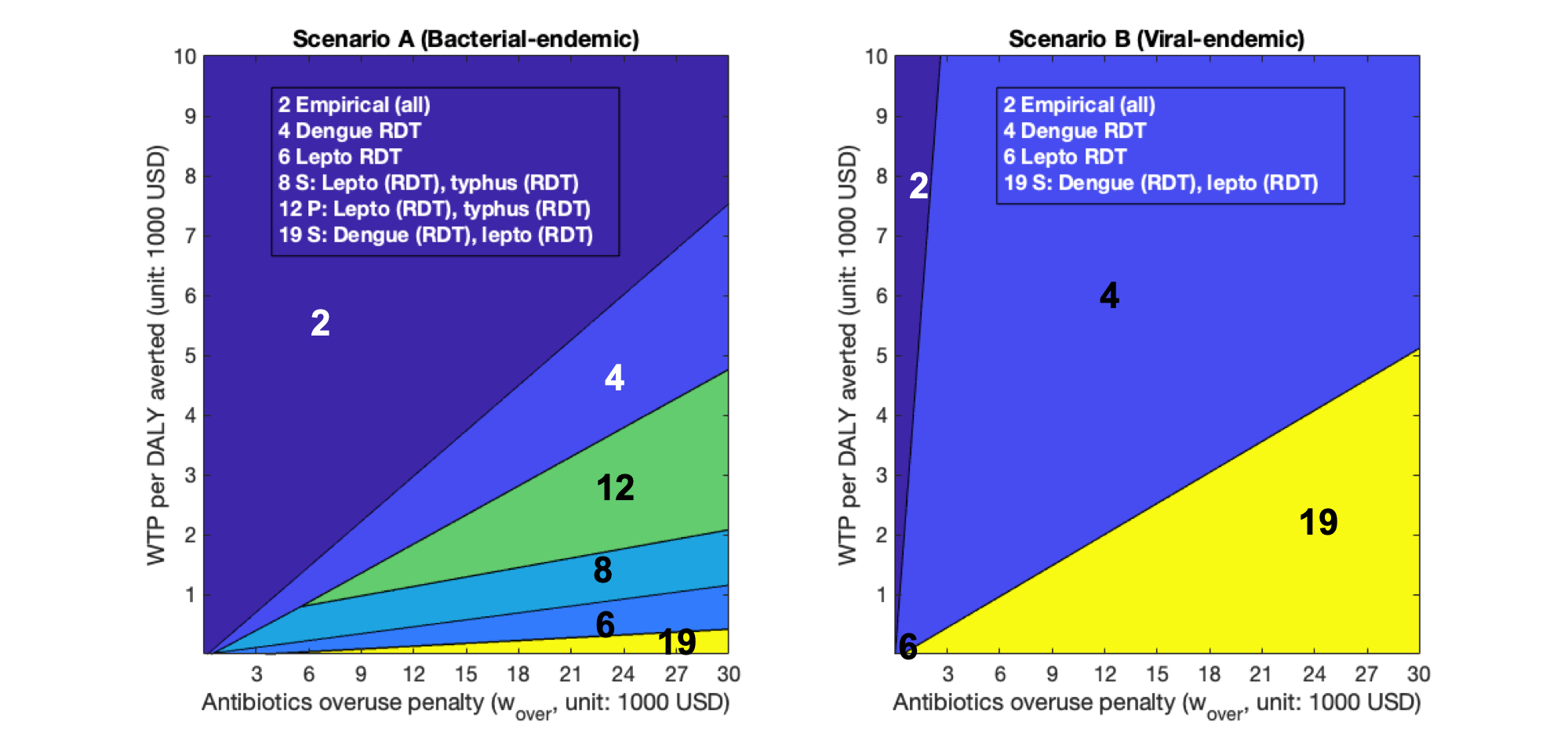


**Reference**

1. Suputtamongkol Y, Pongtavornpinyo W, Lubell Y, Suttinont C, Hoontrakul S, Phimda K, et al. Strategies for Diagnosis and Treatment of Suspected Leptospirosis: A Cost-Benefit Analysis. PLOS Neglected Tropical Diseases. 2010;4(2):e610. doi: 10.1371/journal.pntd.0000610.

2. Watt G, Linda Tuazon MA, Santiago E, Padre L, Calubaquib C, Ranoa C, et al. PLACEBO-CONTROLLED TRIAL OF INTRAVENOUS PENICILLIN FOR SEVERE AND LATE LEPTOSPIROSIS. The Lancet. 1988;331(8583):433-5. doi: <https://doi.org/10.1016/S0140-6736(88)91230-5>.

3. World Health Organization. Global health observatory (GHO) data: World Health Organization; 2015 [cited 2019 June 28]. Available from: <https://www.who.int/gho/mortality_burden_disease/life_tables/life_tables/en/>.

4. Torgerson PR, Hagan JE, Costa F, Calcagno J, Kane M, Martinez-Silveira MS, et al. Global Burden of Leptospirosis: Estimated in Terms of Disability Adjusted Life Years. PLOS Neglected Tropical Diseases. 2015;9(10):e0004122. doi: 10.1371/journal.pntd.0004122.

5. Lee BY, Connor DL, Kitchen SB, Bacon KM, Shah M, Brown ST, et al. Economic value of dengue vaccine in Thailand. The American Society of Tropical Medicine and Hygiene. 2011;84(5):764-72.

6. Suttinont C, Losuwanaluk K, Niwatayakul K, Hoontrakul S, Intaranongpai W, Silpasakorn S, et al. Causes of acute, undifferentiated, febrile illness in rural Thailand: results of a prospective observational study. Annals of Tropical Medicine & Parasitology. 2006;100(4):363-70. doi: 10.1179/136485906X112158.

7. Goris MGA, Leeflang MMG, Loden M, Wagenaar JFP, Klatser PR, Hartskeerl RA, et al. Prospective Evaluation of Three Rapid Diagnostic Tests for Diagnosis of Human Leptospirosis. PLOS Neglected Tropical Diseases. 2013;7(7):e2290. doi: 10.1371/journal.pntd.0002290.

8. Hoontrakul S, Suttinont C, Losuwanaluk K, Suputtamongkol Y. Performance of SD Bioline Tsutsugamushi assays for the diagnosis of scrub typhus in Thailand. J Med Assoc Thai. 2012;95(2):S18-S22.

9. Guzman MG, Harris E. Dengue. The Lancet. 2015;385(9966):453-65. doi: <https://doi.org/10.1016/S0140-6736(14)60572-9>.

10. Ahmed NH, Broor S. Comparison of NS1 antigen detection ELISA, real time RT-PCR and virus isolation for rapid diagnosis of dengue infection in acute phase. Journal of Vector Borne Diseases. 2014;51(3):194.

11. Riediger IN, Stoddard RA, Ribeiro GS, Nakatani SM, Moreira SDR, Skraba I, et al. Rapid, actionable diagnosis of urban epidemic leptospirosis using a pathogenic Leptospira lipL32-based real-time PCR assay. PLOS Neglected Tropical Diseases. 2017;11(9):e0005940. doi: 10.1371/journal.pntd.0005940.

12. Tantibhedhyangkul W, Wongsawat E, Silpasakorn S, Waywa D, Saenyasiri N, Suesuay J, et al. Use of Multiplex Real-Time PCR To Diagnose Scrub Typhus. Journal of Clinical Microbiology. 2017;55(5):1377-87. doi: 10.1128/jcm.02181-16.

13. Waggoner JJ, Abeynayake J, Balassiano I, Lefterova M, Sahoo MK, Liu Y, et al. Multiplex Nucleic Acid Amplification Test for Diagnosis of Dengue Fever, Malaria, and Leptospirosis. Journal of Clinical Microbiology. 2014;52(6):2011-8. doi: 10.1128/jcm.00341-14.

14. Giry C, Roquebert B, Li-Pat-Yuen G, Gasque P, Jaffar-Bandjee M-C. Simultaneous detection of chikungunya virus, dengue virus and human pathogenic Leptospira genomes using a multiplex TaqMan® assay. BMC Microbiology. 2017;17(1):105. doi: 10.1186/s12866-017-1019-1.

15. World Health Organization. Disability-adjusted life year (DALY): World Health Organization; 2012 [cited 2019 June 28]. Available from: <https://www.who.int/healthinfo/global_burden_disease/metrics_daly/en/>.

16. Shrestha P, Cooper BS, Coast J, Oppong R, Do Thi Thuy N, Phodha T, et al. Enumerating the economic cost of antimicrobial resistance per antibiotic consumed to inform the evaluation of interventions affecting their use. Antimicrobial Resistance & Infection Control. 2018;7(1):98. doi: 10.1186/s13756-018-0384-3.

17. Fenwick E, O'Brien BJ, Briggs A. Cost-effectiveness acceptability curves – facts, fallacies and frequently asked questions. Health Economics. 2004;13(5):405-15. doi: 10.1002/hec.903.

18. Johansson KA, Memirie ST, Pecenka C, Jamison DT, Verguet S. Health Gains and Financial Protection from Pneumococcal Vaccination and Pneumonia Treatment in Ethiopia: Results from an Extended Cost-Effectiveness Analysis. PLOS ONE. 2015;10(12):e0142691. doi: 10.1371/journal.pone.0142691.

19. Verguet S, Kim JJ, Jamison DT. Extended Cost-Effectiveness Analysis for Health Policy Assessment: A Tutorial. Pharmacoeconomics. 2016;34(9):913-23. doi: 10.1007/s40273-016-0414-z.

20. Boyd S, Vandenberghe L. Convex optimization: Cambridge university press; 2004.

21. Katz AR, Ansdell VE, Effler PV, Middleton CR, Sasaki DM. Assessment of the Clinical Presentation and Treatment of 353 Cases of Laboratory-Confirmed Leptospirosis in Hawaii, 1974–1998. Clinical Infectious Diseases. 2001;33(11):1834-41. doi: 10.1086/324084.

22. Pokharel S, White LJ, Aguas R, Celhay O, Pellé KG, Dittrich S. Algorithm in the Diagnosis of Febrile Illness Using Pathogen-specific Rapid Diagnostic Tests. Clinical Infectious Diseases. 2019. doi: 10.1093/cid/ciz665.

23. Conroy AL, Gélvez M, Hawkes M, Rajwans N, Liles WC, Villar-Centeno LA, et al. Host biomarkers distinguish dengue from leptospirosis in Colombia: a case–control study. BMC Infectious Diseases. 2014;14(1):35. doi: 10.1186/1471-2334-14-35.

24. Suputtamongkol Y, Niwattayakul K, Suttinont C, Losuwanaluk K, Limpaiboon R, Chierakul W, et al. An Open, Randomized, Controlled Trial of Penicillin, Doxycycline, and Cefotaxime for Patients with Severe Leptospirosis. Clinical Infectious Diseases. 2004;39(10):1417-24. doi: 10.1086/425001.

25. Costa F, Hagan JE, Calcagno J, Kane M, Torgerson P, Martinez-Silveira MS, et al. Global Morbidity and Mortality of Leptospirosis: A Systematic Review. PLOS Neglected Tropical Diseases. 2015;9(9):e0003898. doi: 10.1371/journal.pntd.0003898.

26. Taylor AJ, Paris DH, Newton PN. A Systematic Review of Mortality from Untreated Scrub Typhus (Orientia tsutsugamushi). PLOS Neglected Tropical Diseases. 2015;9(8):e0003971. doi: 10.1371/journal.pntd.0003971.

27. Luh D-L, Liu C-C, Luo Y-R, Chen S-C. Economic cost and burden of dengue during epidemics and non-epidemic years in Taiwan. Journal of Infection and Public Health. 2018;11(2):215-23. doi: <https://doi.org/10.1016/j.jiph.2017.07.021>.

28. Carrasco LR, Lee LK, Lee VJ, Ooi EE, Shepard DS, Thein TL, et al. Economic Impact of Dengue Illness and the Cost-Effectiveness of Future Vaccination Programs in Singapore. PLOS Neglected Tropical Diseases. 2011;5(12):e1426. doi: 10.1371/journal.pntd.0001426.

29. Thanachartwet V, Oer-areemitr N, Chamnanchanunt S, Sahassananda D, Jittmittraphap A, Suwannakudt P, et al. Identification of clinical factors associated with severe dengue among Thai adults: a prospective study. BMC Infectious Diseases. 2015;15(1):420. doi: 10.1186/s12879-015-1150-2.

30. Laoprasopwattana K, Chaimongkol W, Pruekprasert P, Geater A. Acute Respiratory Failure and Active Bleeding Are the Important Fatality Predictive Factors for Severe Dengue Viral Infection. PLOS ONE. 2014;9(12):e114499. doi: 10.1371/journal.pone.0114499.

31. Guzman MG, Halstead SB, Artsob H, Buchy P, Farrar J, Gubler DJ, et al. Dengue: a continuing global threat. Nature Reviews Microbiology. 2010;8:S7. doi: 10.1038/nrmicro2460.

32. Shepard DS, Undurraga EA, Halasa YA. Economic and Disease Burden of Dengue in Southeast Asia. PLOS Neglected Tropical Diseases. 2013;7(2):e2055. doi: 10.1371/journal.pntd.0002055.
