# Supplementary material for: When and what to test for: A cost-effectiveness analysis of febrile illness test-and-treat strategies in the era of responsible antibiotic use
Source: PLoS One. 2020 Jan 8;15(1):e0227409. doi: 10.1371/journal.pone.0227409 (PMC6948826; doi:10.1371/journal.pone.0227409)
Supplement: S1 Table — (DOCX) [file pone.0227409.s003.docx]

**S1 Table: Values for model variables**

| **Variable** | **Base Value** | **Range** | | **Source** |
| --- | --- | --- | --- | --- |
|  |  | **Min** | **Max** |  |
| ***Population*** | | | | |
| Cohort Age | 40 | 20 | 60 | assumed |
| ***Daily transition probabilities: Leptospirosis*** | | | | |
| From Mild (with antibiotics) |  |  |  |  |
| to Mild | 0.510 |  |  | [1] |
| to Severe | 0.000 | 0.000 | 0.000 | [1] |
| to Recovered | 0.490 | 0.243 | 1.000 | [1] |
| From Severe (with antibiotics) |  |  |  |  |
| to Severe | 0.833 |  |  | [2, 3] |
| to Recovered | 0.146 | 0.156 | 0.137 | [2, 3] |
| to Dead | 0.021 | 0.011 | 0.029 |  |
| From Mild (without antibiotics) |  |  |  |  |
| to Mild | 0.815 |  |  | [1] |
| to Severe | 0.074 | 0.067 | 0.083 | [1] |
| to Recovered | 0.111 | 0.100 | 0.125 | [1] |
| From Severe (without antibiotics) |  |  |  |  |
| to Severe | 0.929 |  |  | [4] |
| to Recovered | 0.048 | 0.059 | 0.039 | [4] |
| to Dead | 0.023 | 0.012 | 0.033 | [5] |
|  |  |  |  |  |
| ***Tests Parameters, Leptospirosis*** | | | | |
| Rapid Test-costs, $ | 5.7 | 3 | 7 | [1] |
| Sensitivity ($\leq$ 4 day of illness) | 62% | 41% | 79% | [6] |
| Specificity ($\leq$ 4 day of illness) | 98% | 93% | 99% | [6] |
| Sensitivity ($\geq$ 5 day of illness) | 81% | 69% | 90% | [6] |
| Specificity ($\geq$ 5 day of illness) | 93% | 88% | 96% | [6] |
| Turnaround time, TAT (days) | 1 | 0 | 2 | assumed |
| PCR Test costs, $ | 14 | 10 | 20 | assumed |
| Sensitivity ($\leq$ 4 day of illness) | 85.0% | 73% | 97.50% | [7] |
| Specificity ($\leq$ 4 day of illness) | 95.0% | 90% | 100% | [7] |
| Sensitivity ($\geq$ 5 day of illness) | 41.5% | 26.70% | 57.80% | [7] |
| Specificity ($\geq$ 5 day of illness) | 95.0% | 90% | 100% | [7] |
| Turnaround time, TAT (days) | 2 | 0 | 3 | assumed |
| ***Health Costs, Leptospirosis, $*** | | | | |
| doxycycline prescription (one course) | 2.00 | 1 | 3 | [1] |
| Daily costs in Mild | 5.25 | 5 | 10 | [1] |
| Daily costs in Severe | 48.71 | 30 | 70 | [1] |
| ***DALYs, Leptospirosis*** | | | | |
| DW: Mild | 0.21 | 0.15 | 0.3 | [4] |
| DW: Severe | 0.56 | 0.4 | 0.6 | [4] |
| YLL | 34.90 |  |  | [5, 8] |
| ***Daily transition probabilities, other bacterial*** | | | | |
| From Mild (with antibiotics) |  |  |  |  |
| to Mild | 0.625 |  |  | [1] |
| to Severe | 0.000 | 0.000 | 0.000 | [1] |
| to Recovered | 0.375 | 0.063 | 1.000 | [1] |
| From Severe (with antibiotics) |  |  |  |  |
| to Severe | 0.833 |  |  | [2, 3] |
| to Recovered | 0.146 | 0.156 | 0.137 |  |
| to Dead | 0.021 | 0.011 | 0.029 | [2, 3] |
| From Mild (without antibiotics) |  |  |  |  |
| to Mild | 0.811 |  |  | [1] |
| to Severe | 0.075 | 0.051 | 0.148 | [1] |
| to Recovered | 0.113 | 0.076 | 0.222 | [1] |
| From Severe (without antibiotics) |  |  |  |  |
| to Severe | 0.929 |  |  | [9] |
| to Recovered | 0.048 | 0.059 | 0.039 | [9] |
| to Dead | 0.023 | 0.012 | 0.033 | [9] |
| ***Health Costs, other bacterial, $*** | | | | |
| doxycycline prescription (one course) | 2.00 | 1 | 3 | [1] |
| Daily costs in Mild | 5.25 | 5 | 10 | [1] |
| Daily costs in Severe | 48.71 | 30 | 70 | [1] |
| ***DALYs, other bacterial*** |  |  |  |  |
| DW: Mild | 0.2 | 0.15 | 0.3 | [5] |
| DW: Severe | 0.5 | 0.4 | 0.6 | [5] |
| YLL | 34.9 |  |  | [5, 8] |
| ***Daily transition probabilities, typhus*** | | | | |
| From Mild (with antibiotics) |  |  |  |  |
| to Mild | 0.383 |  |  | [1] |
| to Severe | 0.000 | 0.000 | 0.000 | [1] |
| to Recovered | 0.617 | 0.338 | 1.000 | [1] |
| From Severe (with antibiotics) |  |  |  |  |
| to Severe | 0.833 |  |  | [2, 3] |
| to Recovered | 0.146 | 0.156 | 0.137 | [2, 3] |
| to Dead | 0.021 | 0.011 | 0.029 | [2, 3] |
| From Mild (without antibiotics) |  |  |  |  |
| to Mild | 0.811 |  |  | [1] |
| to Severe | 0.075 | 0.076 | 0.222 | [1] |
| to Recovered | 0.113 | 0.051 | 0.148 | [1] |
| From Severe (without antibiotics) |  |  |  |  |
| to Severe | 0.929 |  |  | [9] |
| to Recovered | 0.048 | 0.059 | 0.039 | [9] |
| to Dead | 0.023 | 0.012 | 0.033 | [9] |
| ***Tests parameters, typhus*** | | | | |
| Rapid Test-costs, $ | 5.7 | 3 | 7 | [1] |
| Sensitivity ($\leq$ 4 day of illness) | 67% | 41% | 79% | [10] |
| Specificity ($\leq$ 4 day of illness) | 98% | 93% | 99% | [10] |
| Sensitivity ($\geq$ 5 day of illness) | 80% | 69% | 90% | assumed similar as lepto |
| Specificity ($\geq$ 5 day of illness) | 95% | 88% | 96% | assumed similar as lepto |
| Turnaround time, TAT (days) | 1 | 0 | 2 | assumed |
| PCR Test costs, $ | 14 | 10 | 20 | assumed |
| Sensitivity ($\leq$ 4 day of illness) | 85.0% | 73% | 97.50% | assumed similar to leptospirosis |
| Specificity ($\leq$ 4 day of illness) | 95.0% | 90% | 100% | assumed similar to leptospirosis |
| Sensitivity ($\geq$ 5 day of illness) | 41.5% | 26.70% | 57.80% | assumed similar to leptospirosis |
| Specificity ($\geq$ 5 day of illness) | 95.0% | 90% | 100% | assumed similar to leptospirosis |
| Turnaround time, TAT (days) | 2 | 0 | 3 | assumed |
| ***Health Costs, typhus, $*** | | | | |
| doxycycline prescription (one course) | 2.00 | 1 | 3 | [1] |
| Daily costs in Mild | 5.25 | 5 | 10 | [1] |
| Daily costs in Severe | 48.71 | 30 | 70 | [1] |
| **DALYs, typhus** |  |  |  |  |
| DW: Mild | 0.21 | 0.15 | 0.3 | [5] |
| DW: Severe | 0.56 | 0 | 0.6 | [5] |
| YLL | 34.90 |  |  | [5, 8] |
| ***Daily transition probabilities, dengue*** | | | | |
| From Mild (with standard care) |  |  |  |  |
| to Mild | 0.815 |  |  | [11, 12] |
| to Severe | 0.027 | 0.061 | 0.214 | [11, 12] |
| to Recovered | 0.159 | 0.010 | 0.036 | [11, 12] |
| From Severe |  |  |  |  |
| to Severe | 0.898 |  |  | [13, 14] |
| to Recovered | 0.089 | 0.095 | 0.073 | [13, 14] |
| to Dead | 0.013 | 0.007 | 0.013 | [13, 14] |
| ***Tests Parameters, dengue*** |  |  |  |  |
| Rapid Test-costs, $ | 5.7 | 3 | 7 | assumed similar as lepto |
| Sensitivity ($\leq$ 4 day of illness) | 68% | 60% | 75% | [15, 16] |
| Specificity ($\leq$ 4 day of illness) | 76% | 71% | 80% | [15, 16] |
| Sensitivity ($\geq$ 5 day of illness) | 90% | 80% | 99% | [15, 16] |
| Specificity ($\geq$ 5 day of illness) | 98% | 95% | 100% | [15, 16] |
| Turnaround time, TAT (days) | 1 | 0 | 2 | assumed |
| PCR Test-costs, $ | 14.00 | 10 | 20 | assumed |
| Sensitivity ($\leq$ 4 day of illness) | 85% | 51% | 100% | [17] |
| Specificity ($\leq$ 4 day of illness) | 100% | 100% | 100% | [17] |
| Sensitivity ($\geq$ 5 day of illness) | 50% | 30% | 70% | [17] |
| Specificity ($\geq$ 5 day of illness) | 100% | 100% | 100% | [17] |
| Turnaround time, TAT (days) | 2 | 0 | 3 | assumed |
| ***Health Costs, dengue*** | | | | |
| Daily costs in Mild, $ | 5.25 | 5 | 10 | [18] |
| Daily costs in Severe, $ | 59.68 | 40 | 60 | [18] |
| ***DALY-related, dengue*** | | | | |
| DW: Mild | 0.20 | 0.15 | 0.3 | [19] |
| DW: Severe | 0.56 | 0.4 | 0.6 | [19] |
| YLL | 34.90 |  |  | [5, 8] |
| ***Real-time Multiplex PCR*** | | | | |
| Price per test, $ | 50 | 30 | 80 | assumed |
| Lepto Sensitivity ($\leq$ 4 day of illness) | 90% | 70% | 100% | assumed |
| Lepto Specificity ($\leq$ 4 day of illness) | 95% | 90% | 100% | assumed |
| Typhus Sensitivity ($\leq$ 4 day of illness) | 87% | 74.2% | 94.4% | [20] |
| Typhus Specificity ($\leq$ 4 day of illness) | 100% | 97.3% | 100% | [20] |
| Dengue Sensitivity ($\leq$ 4 day of illness) | 90% | 70% | 100% | assumed |
| Dengue Specificity ($\leq$ 4 day of illness) | 95% | 90% | 100% | assumed |
| Lepto Sensitivity ($\geq$ 5 day of illness) | 60% | 40% | 80% | assumed |
| Lepto Specificity ($\geq$ 5 day of illness) | 90% | 85% | 98% | assumed |
| Typhus Sensitivity ($\geq$ 5 day of illness) | 55% | 40% | 80% | assumed |
| Typhus Specificity ($\geq$ 5 day of illness) | 97% | 85% | 98% | assumed |
| Dengue Sensitivity ($\geq$ 5 day of illness) | 60% | 40% | 80% | assumed |
| Dengue Specificity ($\geq$ 5 day of illness) | 90% | 85% | 98% | assumed |
| Turnaround time, TAT (days) | 2 | 0 | 3 | assumed |

**Reference**

1. Suputtamongkol Y, Pongtavornpinyo W, Lubell Y, Suttinont C, Hoontrakul S, Phimda K, et al. Strategies for Diagnosis and Treatment of Suspected Leptospirosis: A Cost-Benefit Analysis. PLOS Neglected Tropical Diseases. 2010;4(2):e610. doi: 10.1371/journal.pntd.0000610.

2. Watt G, Linda Tuazon MA, Santiago E, Padre L, Calubaquib C, Ranoa C, et al. PLACEBO-CONTROLLED TRIAL OF INTRAVENOUS PENICILLIN FOR SEVERE AND LATE LEPTOSPIROSIS. The Lancet. 1988;331(8583):433-5. doi: <https://doi.org/10.1016/S0140-6736(88)91230-5>.

3. Suputtamongkol Y, Niwattayakul K, Suttinont C, Losuwanaluk K, Limpaiboon R, Chierakul W, et al. An Open, Randomized, Controlled Trial of Penicillin, Doxycycline, and Cefotaxime for Patients with Severe Leptospirosis. Clinical Infectious Diseases. 2004;39(10):1417-24. doi: 10.1086/425001.

4. Costa F, Hagan JE, Calcagno J, Kane M, Torgerson P, Martinez-Silveira MS, et al. Global Morbidity and Mortality of Leptospirosis: A Systematic Review. PLOS Neglected Tropical Diseases. 2015;9(9):e0003898. doi: 10.1371/journal.pntd.0003898.

5. Torgerson PR, Hagan JE, Costa F, Calcagno J, Kane M, Martinez-Silveira MS, et al. Global Burden of Leptospirosis: Estimated in Terms of Disability Adjusted Life Years. PLOS Neglected Tropical Diseases. 2015;9(10):e0004122. doi: 10.1371/journal.pntd.0004122.

6. Goris MGA, Leeflang MMG, Loden M, Wagenaar JFP, Klatser PR, Hartskeerl RA, et al. Prospective Evaluation of Three Rapid Diagnostic Tests for Diagnosis of Human Leptospirosis. PLOS Neglected Tropical Diseases. 2013;7(7):e2290. doi: 10.1371/journal.pntd.0002290.

7. Riediger IN, Stoddard RA, Ribeiro GS, Nakatani SM, Moreira SDR, Skraba I, et al. Rapid, actionable diagnosis of urban epidemic leptospirosis using a pathogenic Leptospira lipL32-based real-time PCR assay. PLOS Neglected Tropical Diseases. 2017;11(9):e0005940. doi: 10.1371/journal.pntd.0005940.

8. World Health Organization. Global health observatory (GHO) data: World Health Organization; 2015 [cited 2019 June 28]. Available from: <https://www.who.int/gho/mortality_burden_disease/life_tables/life_tables/en/>.

9. Taylor AJ, Paris DH, Newton PN. A Systematic Review of Mortality from Untreated Scrub Typhus (Orientia tsutsugamushi). PLOS Neglected Tropical Diseases. 2015;9(8):e0003971. doi: 10.1371/journal.pntd.0003971.

10. Hoontrakul S, Suttinont C, Losuwanaluk K, Suputtamongkol Y. Performance of SD Bioline Tsutsugamushi assays for the diagnosis of scrub typhus in Thailand. J Med Assoc Thai. 2012;95(2):S18-S22.

11. Luh D-L, Liu C-C, Luo Y-R, Chen S-C. Economic cost and burden of dengue during epidemics and non-epidemic years in Taiwan. Journal of Infection and Public Health. 2018;11(2):215-23. doi: <https://doi.org/10.1016/j.jiph.2017.07.021>.

12. Carrasco LR, Lee LK, Lee VJ, Ooi EE, Shepard DS, Thein TL, et al. Economic Impact of Dengue Illness and the Cost-Effectiveness of Future Vaccination Programs in Singapore. PLOS Neglected Tropical Diseases. 2011;5(12):e1426. doi: 10.1371/journal.pntd.0001426.

13. Thanachartwet V, Oer-areemitr N, Chamnanchanunt S, Sahassananda D, Jittmittraphap A, Suwannakudt P, et al. Identification of clinical factors associated with severe dengue among Thai adults: a prospective study. BMC Infectious Diseases. 2015;15(1):420. doi: 10.1186/s12879-015-1150-2.

14. Laoprasopwattana K, Chaimongkol W, Pruekprasert P, Geater A. Acute Respiratory Failure and Active Bleeding Are the Important Fatality Predictive Factors for Severe Dengue Viral Infection. PLOS ONE. 2014;9(12):e114499. doi: 10.1371/journal.pone.0114499.

15. Guzman MG, Halstead SB, Artsob H, Buchy P, Farrar J, Gubler DJ, et al. Dengue: a continuing global threat. Nature Reviews Microbiology. 2010;8:S7. doi: 10.1038/nrmicro2460.

16. Guzman MG, Harris E. Dengue. The Lancet. 2015;385(9966):453-65. doi: <https://doi.org/10.1016/S0140-6736(14)60572-9>.

17. Ahmed NH, Broor S. Comparison of NS1 antigen detection ELISA, real time RT-PCR and virus isolation for rapid diagnosis of dengue infection in acute phase. Journal of Vector Borne Diseases. 2014;51(3):194.

18. Shepard DS, Undurraga EA, Halasa YA. Economic and Disease Burden of Dengue in Southeast Asia. PLOS Neglected Tropical Diseases. 2013;7(2):e2055. doi: 10.1371/journal.pntd.0002055.

19. Lee BY, Connor DL, Kitchen SB, Bacon KM, Shah M, Brown ST, et al. Economic value of dengue vaccine in Thailand. The American Society of Tropical Medicine and Hygiene. 2011;84(5):764-72.

20. Tantibhedhyangkul W, Wongsawat E, Silpasakorn S, Waywa D, Saenyasiri N, Suesuay J, et al. Use of Multiplex Real-Time PCR To Diagnose Scrub Typhus. Journal of Clinical Microbiology. 2017;55(5):1377-87. doi: 10.1128/jcm.02181-16.
