# Supplementary material for: When and what to test for: A cost-effectiveness analysis of febrile illness test-and-treat strategies in the era of responsible antibiotic use
Source: PLoS One. 2020 Jan 8;15(1):e0227409. doi: 10.1371/journal.pone.0227409 (PMC6948826; doi:10.1371/journal.pone.0227409)
Supplement: S2 Table — We fixed the disease occurrence probabilities to the ones in Scenario A, and set the WTP to Thailand GDP per capita. We assumed that patients presented to hospitals on day four (average day of illness). Strategy 2: Empirical to all; Strategy 5: Dengue PCR; Strategy 9: S: Lepto PCR, typhus RDT; Strategy 13: P: Lepto PCR, typhus RDT; Strategy 15: Multiplex PCR. (DOCX) [file pone.0227409.s004.docx]

**S2 Table: Results of one-way sensitivity analysis (numbers are the corresponding strategy orders).** We fixed the disease occurrence probabilities to the ones in Scenario A, and set the WTP to Thailand GDP per capita. We assumed that patients presented to hospitals on day four (average day of illness). Strategy 2: Empirical to all; Strategy 5: Dengue PCR; Strategy 9: S: Lepto PCR, typhus RDT; Strategy 13: P: Lepto PCR, typhus RDT; Strategy 15: Multiplex PCR.

| **Variable** | **Parameter Value (min/max)** | **Penalty on antibiotic overuse (in USD)** | | |
| --- | --- | --- | --- | --- |
|  |  | **$0** | **$10,000** | **$50,000** |
| *Base-case optimal strategy sequence* | | 2 | 2 | 15 |
| Cohort Age | 40 (max) | 2 | 15 | 15 |
| Probability from Severe to Death (with antibiotics) | 0.029 (max) | 2 | 15 | 15 |
| Lepto PCR sensitivity | 97.50% (max) | 2 | 2 | 13 |
| Lepto PCR turnaround time (days) | 0 day (min) | 2 | 2 | 9 |
| Dengue PCR turnaround time (days) | 0 day (min) | 2 | 5 | 15 |
| Multiple PCR sensitivity | 70% (min) | 2 | 2 | 13 |
| *All the remaining parameters in S1 Table, either with their min or max values* | | 2 | 2 | 15 |
