# Supplementary material for: When and what to test for: A cost-effectiveness analysis of febrile illness test-and-treat strategies in the era of responsible antibiotic use
Source: PLoS One. 2020 Jan 8;15(1):e0227409. doi: 10.1371/journal.pone.0227409 (PMC6948826; doi:10.1371/journal.pone.0227409)
Supplement: S3 Table — For each scenario, we identified the strategies that were on the three-dimensional effectiveness frontier, where the three dimensions are DALY, cost and antibiotic overuse. * = strategies on the effectiveness frontier (economically efficient) for Scenario A (bacterial-endemic); º = strategies on the effectiveness frontier (economically efficient) for Scenario B (viral-endemic). (DOCX) [file pone.0227409.s005.docx]

**S3 Table: Strategy outcomes: per-patient costs and disability-adjusted life years (DALYs) incurred, antibiotic overuse (Prob(over)) and underuse (Prob(under)) and for patients seeking care on the first day of illness.**

| Strategies/Scenarios | | *Scenario A: Bacterial-Endemic* | | | | *Scenario B: Viral-Endemic* | | | |
| --- | --- | --- | --- | --- | --- | --- | --- | --- | --- |
|  |  | ***Cost*** | ***DALY*** | ***P(over)*** | ***P(under)*** | ***Cost*** | ***DALY*** | ***P(over)*** | ***P(under)*** |
| 1 | No Antibiotics *º | 216.166 | 2.911 | 0.000 | 0.619 | 138.978 | 1.258 | 0.000 | 0.171 |
| 2 | Empirical All *º | 47.549 | 0.239 | 0.381 | 0.000 | 94.107 | 0.520 | 0.829 | 0.000 |
| 3 | Empirical Severe º | 130.630 | 1.323 | 0.055 | 0.248 | 116.784 | 0.819 | 0.119 | 0.068 |
| 4 | Dengue RDT | 104.111 | 1.034 | 0.198 | 0.132 | 112.024 | 0.739 | 0.282 | 0.036 |
| 5 | Dengue PCR *º | 85.512 | 0.628 | 0.197 | 0.000 | 110.094 | 0.628 | 0.184 | 0.000 |
| 6 | Lepto RDT *º | 137.518 | 1.576 | 0.006 | 0.253 | 132.471 | 1.078 | 0.014 | 0.112 |
| 7 | Lepto PCR * | 122.619 | 1.239 | 0.014 | 0.131 | 134.053 | 1.025 | 0.029 | 0.085 |
| 8 | S: Lepto RDT, typhus RDT º | 129.498 | 1.462 | 0.011 | 0.430 | 129.077 | 1.036 | 0.024 | 0.192 |
| 9 | S: Lepto PCR, typhus RDT *º | 119.643 | 1.142 | 0.017 | 0.216 | 134.098 | 0.988 | 0.037 | 0.146 |
| 10 | S: Lepto RDT, typhus PCR | 127.176 | 1.440 | 0.014 | 0.387 | 128.716 | 1.027 | 0.031 | 0.173 |
| 11 | P: Lepto PCR, typhus PCR | 131.534 | 1.079 | 0.026 | 0.097 | 148.671 | 0.958 | 0.057 | 0.071 |
| 12 | P: Lepto RDT, typhus RDT | 135.543 | 1.437 | 0.013 | 0.222 | 135.924 | 1.025 | 0.028 | 0.100 |
| 13 | P: Lepto PCR, typhus RDT | 125.833 | 1.122 | 0.019 | 0.106 | 141.617 | 0.979 | 0.041 | 0.075 |
| 14 | P: Lepto RDT, typhus PCR | 148.592 | 1.492 | 0.019 | 0.186 | 144.128 | 1.020 | 0.041 | 0.084 |
| 15 | Multiplex PCR*º | 148.696 | 1.001 | 0.014 | 0.080 | 170.800 | 0.961 | 0.029 | 0.072 |

For each scenario, we identified the strategies that were on the three-dimensional effectiveness frontier, where the three dimensions are DALY, cost and antibiotic overuse. * = strategies on the effectiveness frontier (economically efficient) for Scenario A (bacterial-endemic); º = strategies on the effectiveness frontier (economically efficient) for Scenario B (viral-endemic).
