# Supplementary material for: When and what to test for: A cost-effectiveness analysis of febrile illness test-and-treat strategies in the era of responsible antibiotic use
Source: PLoS One. 2020 Jan 8;15(1):e0227409. doi: 10.1371/journal.pone.0227409 (PMC6948826; doi:10.1371/journal.pone.0227409)
Supplement: S4 Table — For each scenario, we identified the strategies that were on the three-dimensional effectiveness frontier, where the three dimensions are DALY, cost and antibiotic overuse. * = strategies on the effectiveness frontier (economically efficient) for Scenario A (bacterial-endemic); º = strategies on the effectiveness frontier (economically efficient) for Scenario B (viral-endemic). (DOCX) [file pone.0227409.s006.docx]

**S4 Table: Strategy outcomes: per-patient costs and disability-adjusted life years (DALYs) incurred, antibiotic overuse (*Prob(over)*) and underuse (*Prob(under)*) and for patients seeking care on the tenth day of illness.**

| Strategies/Scenarios | | *Scenario A: Bacterial-Endemic* | | | | *Scenario B: Viral-Endemic* | | | |
| --- | --- | --- | --- | --- | --- | --- | --- | --- | --- |
|  |  | ***Cost*** | ***DALY*** | ***P(over)*** | ***P(under)*** | ***Cost*** | ***DALY*** | ***P(over)*** | ***P(under)*** |
| 1 | No Antibiotics *º | 216.166 | 2.911 | 0.000 | 0.220 | 138.978 | 1.258 | 0.000 | 0.060 |
| 2 | Empirical All *º | 149.508 | 1.720 | 0.075 | 0.000 | 122.128 | 0.930 | 0.163 | 0.000 |
| 3 | Empirical Severe º | 160.095 | 1.857 | 0.055 | 0.248 | 124.963 | 0.967 | 0.119 | 0.068 |
| 4 | Dengue RDT | 155.717 | 1.838 | 0.045 | 0.004 | 122.864 | 0.962 | 0.038 | 0.001 |
| 5 | Dengue PCR *º | 160.426 | 1.906 | 0.047 | 0.000 | 124.658 | 0.981 | 0.072 | 0.000 |
| 6 | Lepto RDT *º | 172.964 | 2.142 | 0.005 | 0.060 | 133.316 | 1.147 | 0.010 | 0.035 |
| 7 | Lepto PCR * | 196.730 | 2.548 | 0.003 | 0.117 | 137.110 | 1.203 | 0.006 | 0.041 |
| 8 | S: Lepto RDT, typhus RDT º | 172.399 | 2.084 | 0.007 | 0.099 | 134.200 | 1.124 | 0.015 | 0.060 |
| 9 | S: Lepto PCR, typhus RDT *º | 201.927 | 2.483 | 0.005 | 0.203 | 145.614 | 1.181 | 0.010 | 0.070 |
| 10 | S: Lepto RDT, typhus PCR | 172.814 | 2.118 | 0.006 | 0.096 | 133.345 | 1.138 | 0.014 | 0.057 |
| 11 | P: Lepto PCR, typhus PCR | 220.263 | 2.490 | 0.005 | 0.107 | 162.760 | 1.183 | 0.012 | 0.037 |
| 12 | P: Lepto RDT, typhus RDT | 179.633 | 2.070 | 0.008 | 0.046 | 142.403 | 1.117 | 0.016 | 0.029 |
| 13 | P: Lepto PCR, typhus RDT | 210.424 | 2.463 | 0.005 | 0.102 | 153.892 | 1.173 | 0.012 | 0.035 |
| 14 | P: Lepto RDT, typhus PCR | 193.554 | 2.165 | 0.006 | 0.047 | 151.943 | 1.138 | 0.014 | 0.029 |
| 15 | Multiplex PCR*º | 233.338 | 2.332 | 0.007 | 0.078 | 183.249 | 1.156 | 0.015 | 0.032 |

For each scenario, we identified the strategies that were on the three-dimensional effectiveness frontier, where the three dimensions are DALY, cost and antibiotic overuse. * = strategies on the effectiveness frontier (economically efficient) for Scenario A (bacterial-endemic); º = strategies on the effectiveness frontier (economically efficient) for Scenario B (viral-endemic).
