# Supplementary material for: When and what to test for: A cost-effectiveness analysis of febrile illness test-and-treat strategies in the era of responsible antibiotic use
Source: PLoS One. 2020 Jan 8;15(1):e0227409. doi: 10.1371/journal.pone.0227409 (PMC6948826; doi:10.1371/journal.pone.0227409)
Supplement: S5 Table — Values obtained from [6] (DOCX) [file pone.0227409.s007.docx]

**S5 Table: Sensitivity analysis of disease occurrence probabilities** Values obtained from [6]

| *Probability of infections* | *leptospirosis* | *typhus* | *Other bacterial* | *dengue* | *other* |
| --- | --- | --- | --- | --- | --- |
| SEt 1 | 0.358 | 0.209 | 0.047 | 0.071 | 0.315 |
| SET 2 | 0.076 | 0.204 | 0.022 | 0.222 | 0.476 |
