# Supplementary material for: When and what to test for: A cost-effectiveness analysis of febrile illness test-and-treat strategies in the era of responsible antibiotic use
Source: PLoS One. 2020 Jan 8;15(1):e0227409. doi: 10.1371/journal.pone.0227409 (PMC6948826; doi:10.1371/journal.pone.0227409)
Supplement: S6 Table — * = strategies on the effectiveness frontier (economically efficient) for Scenario A (bacterial-endemic); º = strategies on the effectiveness frontier (economically efficient) for Scenario B (viral-endemic). (DOCX) [file pone.0227409.s008.docx]

**S6 Table: Per-patient costs (USD), DALYs incurred, antibiotic overuse (*Prob(over)*) and underuse (*Prob(under)*) for febrile patients seeking care on the fourth day (average day) of illness and undergoing various test and treat strategies, with 75% antibiotic effectiveness.**

| Strategies | | *Scenario A: Bacterial-Endemic* | | | | *Scenario B: Viral-Endemic* | | | |
| --- | --- | --- | --- | --- | --- | --- | --- | --- | --- |
|  |  | ***Cost*** | ***DALY*** | ***P(over)*** | ***P(under)*** | ***Cost*** | ***DALY*** | ***P(over)*** | ***P(under)*** |
| 1 | No Antibiotics *º | 216.166 | 2.911 | 0.000 | 0.394 | 138.978 | 1.258 | 0.000 | 0.109 |
| 2 | Empirical All *º | 107.943 | 1.074 | 0.193 | 0.000 | 110.656 | 0.750 | 0.421 | 0.000 |
| 3 | Empirical Severe | 137.058 | 1.440 | 0.055 | 0.248 | 118.573 | 0.851 | 0.119 | 0.068 |
| 4 | Dengue RDT | 141.889 | 1.614 | 0.102 | 0.085 | 120.261 | 0.900 | 0.144 | 0.023 |
| 5 | Dengue PCR º | 127.833 | 1.327 | 0.102 | 0.000 | 117.781 | 0.821 | 0.095 | 0.000 |
| 6 | Lepto RDT *º | 163.998 | 1.992 | 0.003 | 0.163 | 133.758 | 1.134 | 0.007 | 0.072 |
| 7 | Lepto PCR * | 152.617 | 1.754 | 0.007 | 0.086 | 133.705 | 1.097 | 0.015 | 0.056 |
| 8 | S: Lepto RDT, typhus RDT º | 159.298 | 1.911 | 0.006 | 0.280 | 132.052 | 1.104 | 0.012 | 0.125 |
| 9 | S: Lepto PCR, typhus RDT *º | 155.984 | 1.685 | 0.009 | 0.143 | 138.665 | 1.071 | 0.019 | 0.096 |
| 10 | S: Lepto RDT, typhus PCR º | 156.261 | 1.897 | 0.008 | 0.254 | 130.067 | 1.099 | 0.016 | 0.113 |
| 11 | P: Lepto PCR, typhus PCR | 169.802 | 1.640 | 0.014 | 0.063 | 154.814 | 1.049 | 0.030 | 0.046 |
| 12 | P: Lepto RDT, typhus RDT | 167.390 | 1.892 | 0.007 | 0.143 | 140.858 | 1.096 | 0.014 | 0.064 |
| 13 | P: Lepto PCR, typhus RDT | 163.273 | 1.670 | 0.010 | 0.069 | 147.360 | 1.063 | 0.021 | 0.049 |
| 14 | P: Lepto RDT, typhus PCR | 178.179 | 1.925 | 0.010 | 0.121 | 148.990 | 1.091 | 0.021 | 0.055 |
| 15 | Multiplex PCR*º | 188.618 | 1.585 | 0.007 | 0.052 | 176.905 | 1.051 | 0.015 | 0.047 |

* = strategies on the effectiveness frontier (economically efficient) for Scenario A (bacterial-endemic); º = strategies on the effectiveness frontier (economically efficient) for Scenario B (viral-endemic).
