# Supplementary material for: When and what to test for: A cost-effectiveness analysis of febrile illness test-and-treat strategies in the era of responsible antibiotic use
Source: PLoS One. 2020 Jan 8;15(1):e0227409. doi: 10.1371/journal.pone.0227409 (PMC6948826; doi:10.1371/journal.pone.0227409)
Supplement: S7 Table — * = strategies on the effectiveness frontier (economically efficient) for Scenario A (bacterial-endemic); º = strategies on the effectiveness frontier (economically efficient) for Scenario B (viral-endemic). (DOCX) [file pone.0227409.s009.docx]

**S7 Table: Per-patient costs (USD), DALYs incurred, antibiotic overuse (*Prob(over)*) and underuse (*Prob(under)*) for febrile patients seeking care on the fourth day (average day) of illness and undergoing various test and treat strategies, with 50% antibiotic effectiveness.**

| Strategies | | *Scenario A: Bacterial-Endemic* | | | | *Scenario B: Viral-Endemic* | | | |
| --- | --- | --- | --- | --- | --- | --- | --- | --- | --- |
|  |  | ***Cost*** | ***DALY*** | ***P(over)*** | ***P(under)*** | ***Cost*** | ***DALY*** | ***P(over)*** | ***P(under)*** |
| 1 | No Antibiotics *º | 216.166 | 2.911 | 0.000 | 0.394 | 138.978 | 1.258 | 0.000 | 0.109 |
| 2 | Empirical All *º | 115.311 | 1.185 | 0.193 | 0.000 | 112.619 | 0.780 | 0.421 | 0.000 |
| 3 | Empirical Severe º | 137.058 | 1.440 | 0.055 | 0.248 | 118.573 | 0.851 | 0.119 | 0.068 |
| 4 | Dengue RDT | 146.449 | 1.682 | 0.102 | 0.085 | 121.474 | 0.918 | 0.144 | 0.023 |
| 5 | Dengue PCR *º | 132.720 | 1.400 | 0.102 | 0.000 | 119.078 | 0.840 | 0.095 | 0.000 |
| 6 | Lepto RDT *º | 167.497 | 2.044 | 0.003 | 0.163 | 134.225 | 1.141 | 0.007 | 0.072 |
| 7 | Lepto PCR * | 156.471 | 1.812 | 0.007 | 0.086 | 134.235 | 1.105 | 0.015 | 0.056 |
| 8 | S: Lepto RDT, typhus RDT º | 162.830 | 1.964 | 0.006 | 0.280 | 132.534 | 1.112 | 0.012 | 0.125 |
| 9 | S: Lepto PCR, typhus RDT *º | 159.851 | 1.743 | 0.009 | 0.143 | 139.205 | 1.079 | 0.019 | 0.096 |
| 10 | S: Lepto RDT, typhus PCR º | 159.814 | 1.950 | 0.008 | 0.254 | 130.558 | 1.106 | 0.016 | 0.113 |
| 11 | P: Lepto PCR, typhus PCR | 173.708 | 1.698 | 0.014 | 0.063 | 155.382 | 1.057 | 0.030 | 0.046 |
| 12 | P: Lepto RDT, typhus RDT | 170.938 | 1.945 | 0.007 | 0.143 | 141.347 | 1.103 | 0.014 | 0.064 |
| 13 | P: Lepto PCR, typhus RDT | 167.147 | 1.728 | 0.010 | 0.069 | 147.905 | 1.072 | 0.021 | 0.049 |
| 14 | P: Lepto RDT, typhus PCR | 181.131 | 1.969 | 0.010 | 0.121 | 149.416 | 1.098 | 0.021 | 0.055 |
| 15 | Multiplex PCR*º | 192.698 | 1.647 | 0.007 | 0.052 | 177.463 | 1.059 | 0.015 | 0.047 |

* = strategies on the effectiveness frontier (economically efficient) for Scenario A (bacterial-endemic); º = strategies on the effectiveness frontier (economically efficient) for Scenario B (viral-endemic).
