# Supplementary material for: When and what to test for: A cost-effectiveness analysis of febrile illness test-and-treat strategies in the era of responsible antibiotic use
Source: PLoS One. 2020 Jan 8;15(1):e0227409. doi: 10.1371/journal.pone.0227409 (PMC6948826; doi:10.1371/journal.pone.0227409)
Supplement: S8 Table — (DOCX) [file pone.0227409.s010.docx]

|  | ***Strategy*** | ***Test interpretation treatment decision*** |
| --- | --- | --- |
| 16 | S: Dengue PCR, lepto PCR | Dengue PCR positive: out(in)patient care w/o antibiotics  Dengue PCR negative: perform Lepto PCR  Lepto PCR positive: antibiotic  Lepto PCR negative: out(in)patient care w/o antibiotics |
| 17 | S: Dengue PCR, lepto RDT | Dengue PCR positive: out(in)patient care w/o antibiotics  Dengue PCR negative: perform Lepto RDT  Lepto RDT positive: antibiotic  Lepto RDT negative: out(in)patient care w/o antibiotics |
| 18 | S: Dengue RDT, lepto PCR | Dengue RDT positive: out(in)patient care w/o antibiotics  Dengue RDT negative: perform Lepto PCR  Lepto PCR positive: antibiotic  Lepto PCR negative: out(in)patient care w/o antibiotics |
| 19 | S: Dengue RDT, lepto RDT | Dengue RDT positive: out(in)patient care w/o antibiotics  Dengue RDT negative: perform Lepto RDT  Lepto RDT positive: antibiotic  Lepto RDT negative: out(in)patient care w/o antibiotics |

**S8 Table: Additional Strategies evaluated for two Thai settings using Markov cohort models**
