# Supplementary material for: When and what to test for: A cost-effectiveness analysis of febrile illness test-and-treat strategies in the era of responsible antibiotic use
Source: PLoS One. 2020 Jan 8;15(1):e0227409. doi: 10.1371/journal.pone.0227409 (PMC6948826; doi:10.1371/journal.pone.0227409)
Supplement: S9 Table — * = strategies on the effectiveness frontier (economically efficient) for Scenario A (bacterial-endemic); º = strategies on the effectiveness frontier (economically efficient) for Scenario B (viral-endemic). (DOCX) [file pone.0227409.s011.docx]

**S9 Table: Per-patient costs (USD), DALYs incurred, antibiotic overuse (*Prob(over)*) and underuse (*Prob(under)*) for febrile patients on different days of presentation and undergoing various test and treat strategies.**

| Strategies | | *Scenario A: Bacterial-Endemic* | | | | *Scenario B: Viral-Endemic* | | | |
| --- | --- | --- | --- | --- | --- | --- | --- | --- | --- |
|  |  | ***Cost*** | ***DALY*** | ***P(over)*** | ***P(under)*** | ***Cost*** | ***DALY*** | ***P(over)*** | ***P(under)*** |
| First (min) day of illness | | | | | | | | | |
| 16 | S: Dengue PCR, lepto PCR º | 150.216 | 1.702 | 0.005 | 0.086 | 140.603 | 1.093 | 0.005 | 0.056 |
| 17 | S: Dengue PCR, lepto RDT *º | 162.273 | 1.864 | 0.003 | 0.181 | 142.908 | 1.119 | 0.003 | 0.080 |
| 18 | S: Dengue RDT, lepto PCR º | 157.232 | 1.915 | 0.005 | 0.204 | 135.054 | 1.122 | 0.007 | 0.083 |
| 19 | S: Dengue RDT, lepto RDT º | 166.438 | 2.049 | 0.003 | 0.285 | 136.533 | 1.143 | 0.004 | 0.104 |
| Fourth (average) day of illness | | | | | | | | | |
| 16 | S: Dengue PCR, lepto PCR º | 169.362 | 2.044 | 0.003 | 0.058 | 142.491 | 1.141 | 0.003 | 0.038 |
| 17 | S: Dengue PCR, lepto RDT *º | 179.805 | 2.155 | 0.001 | 0.121 | 144.863 | 1.158 | 0.001 | 0.053 |
| 18 | S: Dengue RDT, lepto PCR º | 172.103 | 2.194 | 0.003 | 0.134 | 135.707 | 1.161 | 0.004 | 0.055 |
| 19 | S: Dengue RDT, lepto RDT º | 180.463 | 2.287 | 0.001 | 0.187 | 137.694 | 1.175 | 0.002 | 0.068 |
| Tenth (max) day of illness | | | | | | | | | |
| 16 | S: Dengue PCR, lepto PCR | 207.182 | 2.670 | 0.001 | 0.084 | 147.064 | 1.225 | 0.002 | 0.029 |
| 17 | S: Dengue PCR, lepto RDT | 191.320 | 2.322 | 0.002 | 0.046 | 145.790 | 1.175 | 0.004 | 0.027 |
| 18 | S: Dengue RDT, lepto PCR | 197.713 | 2.652 | 0.001 | 0.093 | 139.508 | 1.222 | 0.001 | 0.032 |
| 19 | S: Dengue RDT, lepto RDT*º | 180.539 | 2.280 | 0.002 | 0.053 | 137.739 | 1.169 | 0.002 | 0.030 |

* = strategies on the effectiveness frontier (economically efficient) for Scenario A (bacterial-endemic); º = strategies on the effectiveness frontier (economically efficient) for Scenario B (viral-endemic).
