# Supplementary figures and images for: When and what to test for: A cost-effectiveness analysis of febrile illness test-and-treat strategies in the era of responsible antibiotic use
Source: PLoS One. 2020 Jan 8;15(1):e0227409. doi: 10.1371/journal.pone.0227409 (PMC6948826; doi:10.1371/journal.pone.0227409)

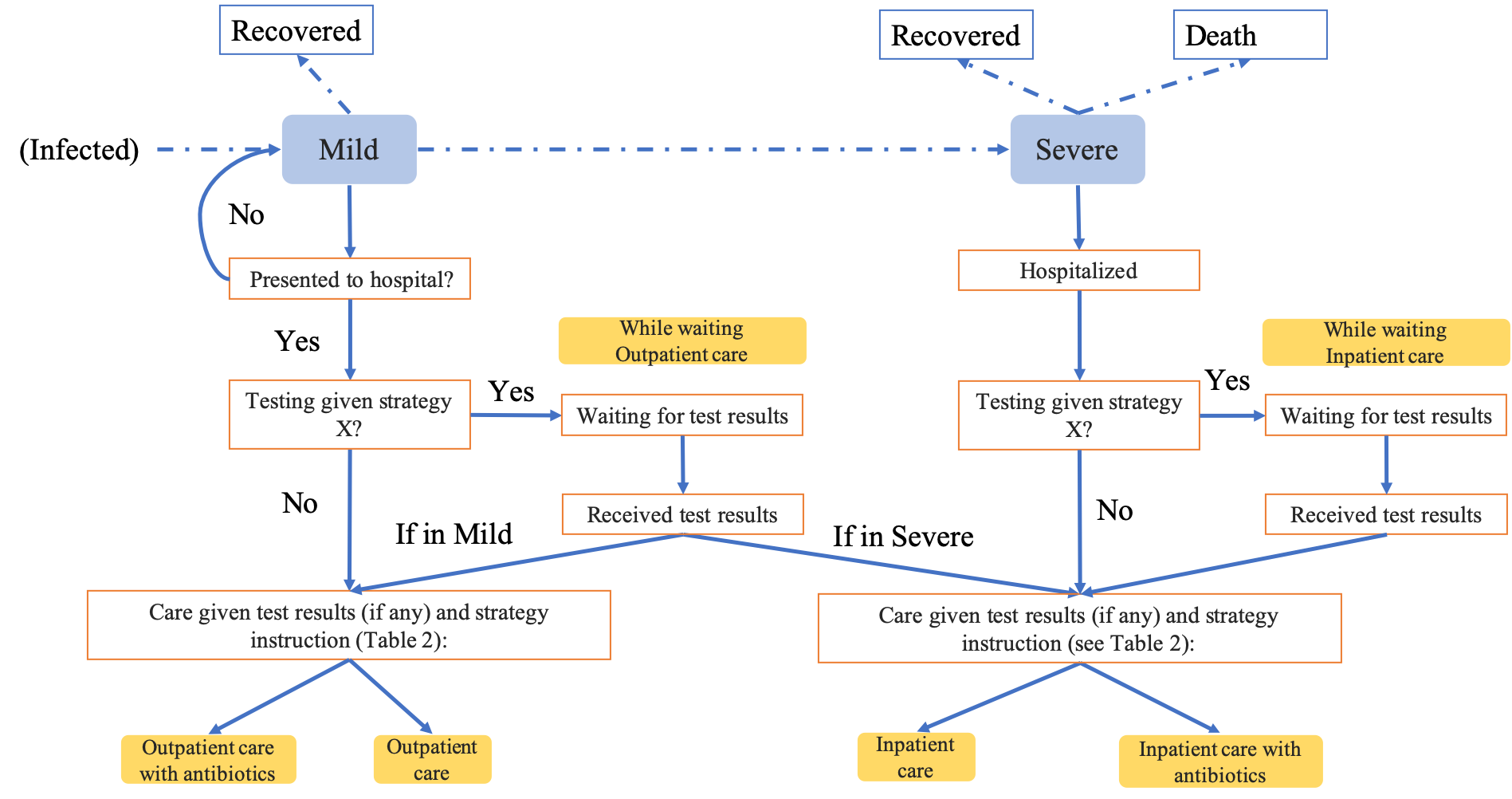

Supplement: S1 Fig — (TIFF) [file pone.0227409.s012.tiff]

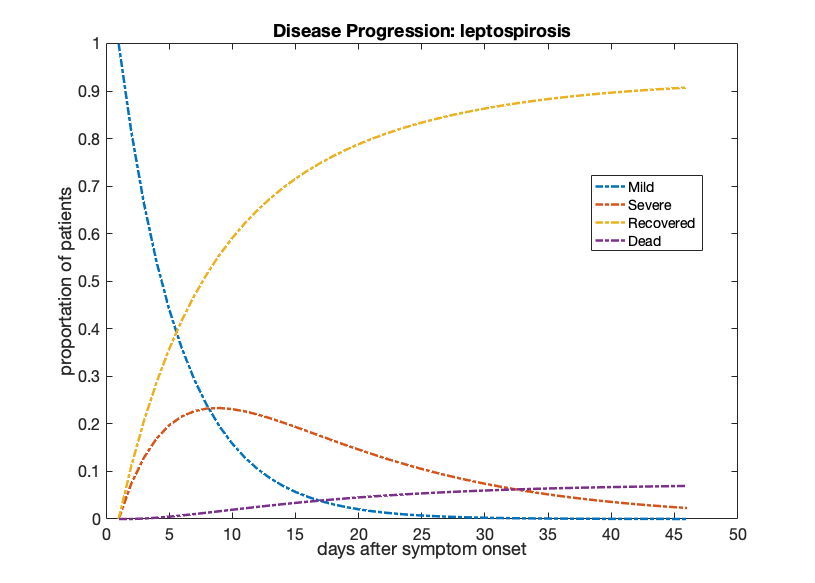

Supplement: S2 Fig — Patients enter the model in Mild state, during the 45-day horizon, they could progress to Severe state, become recovered, or dead. (TIF) [file pone.0227409.s013.tif]

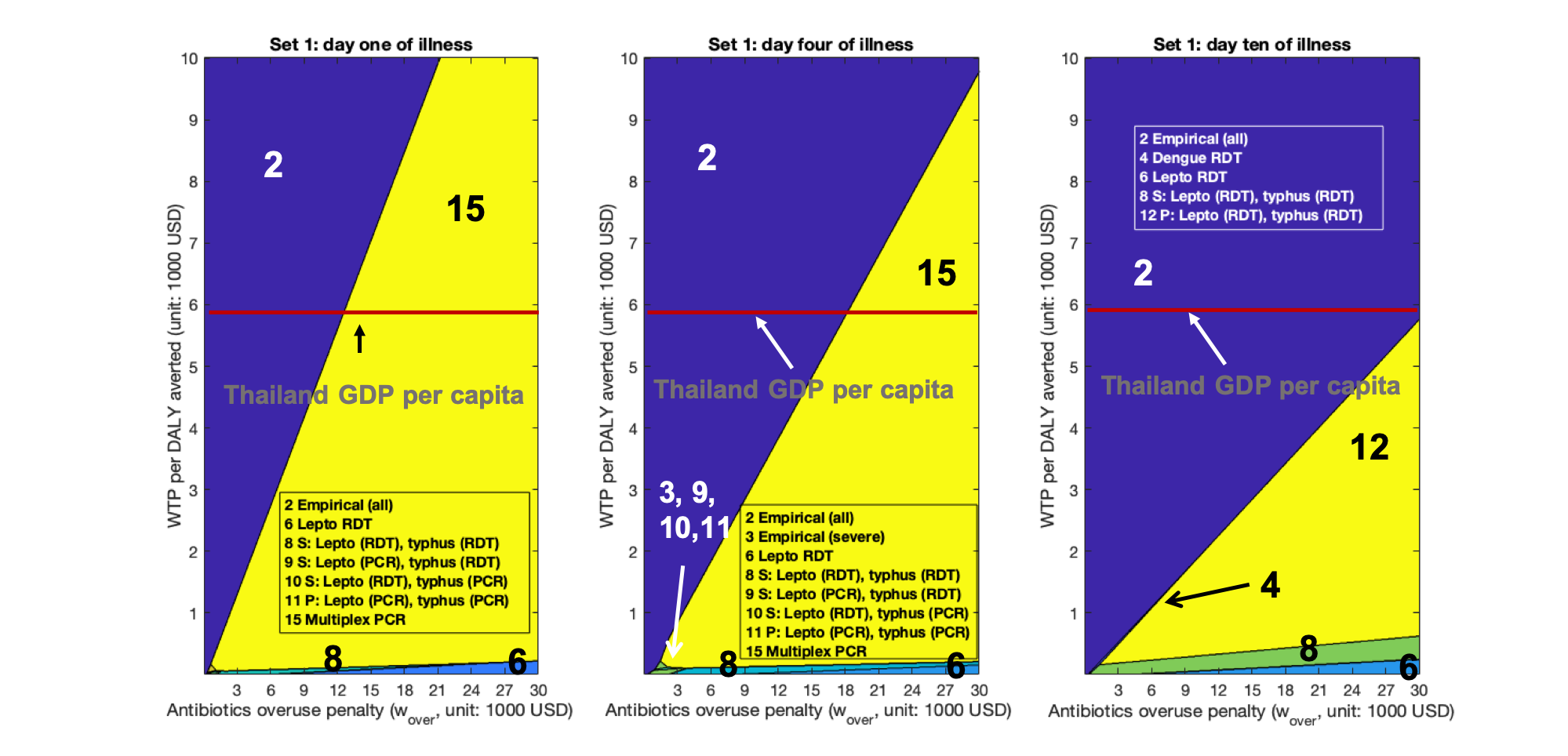

Supplement: S3 Fig — We fixed disease occurrence probability vector as Set 1 in S5 Table. NMB = net monetary benefit, WTP = willingness-to-pay, wover = antibiotic overuse penalty. (TIF) [file pone.0227409.s014.tif]

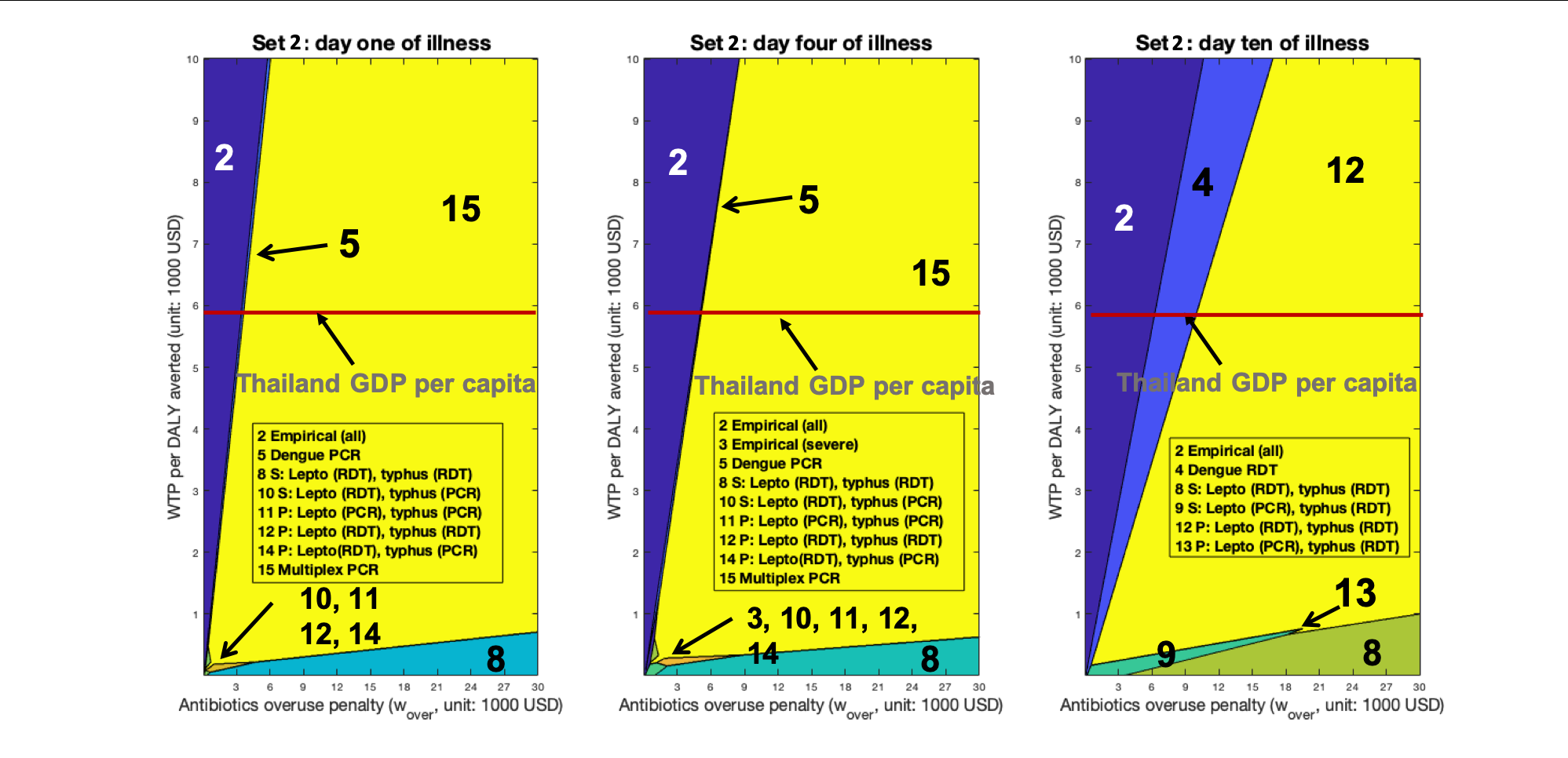

Supplement: S4 Fig — We fixed disease occurrence probability as Set 2 in S5 Table. NMB = net monetary benefit, WTP = willingness-to-pay, wover = antibiotic overuse penalty. (TIF) [file pone.0227409.s015.tif]

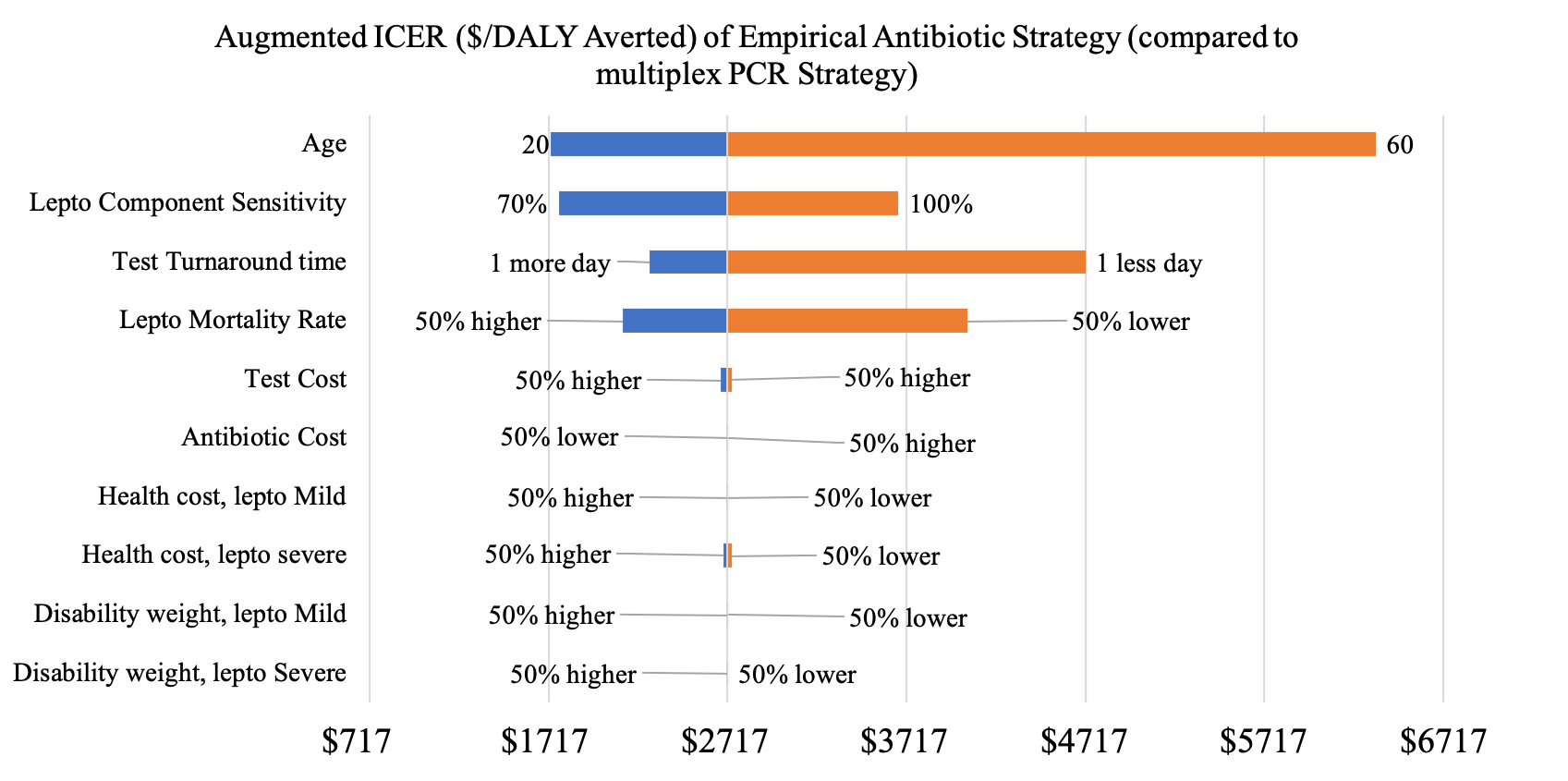

Supplement: S5 Fig — One-way sensitivity analysis, each row (bar) displays the range of Augmented ICER between the empirical antibiotic to all strategy and the Multiplex PCR strategy (patients present to a hospital on day one). We only displayed leptospirosis-specific disease parameters, but all other disease categories share the same structure in the range of ICER change. Augmented ICER = augmented incremental cost-effectiveness ratio, calculated by the ratio of DALY difference and augmented cost difference. Augmented cost = cost + penalty * wover; DALY = disability-adjusted life year; PCR = Polymerase Chain Reaction tests. (TIFF) [file pone.0227409.s016.tiff]

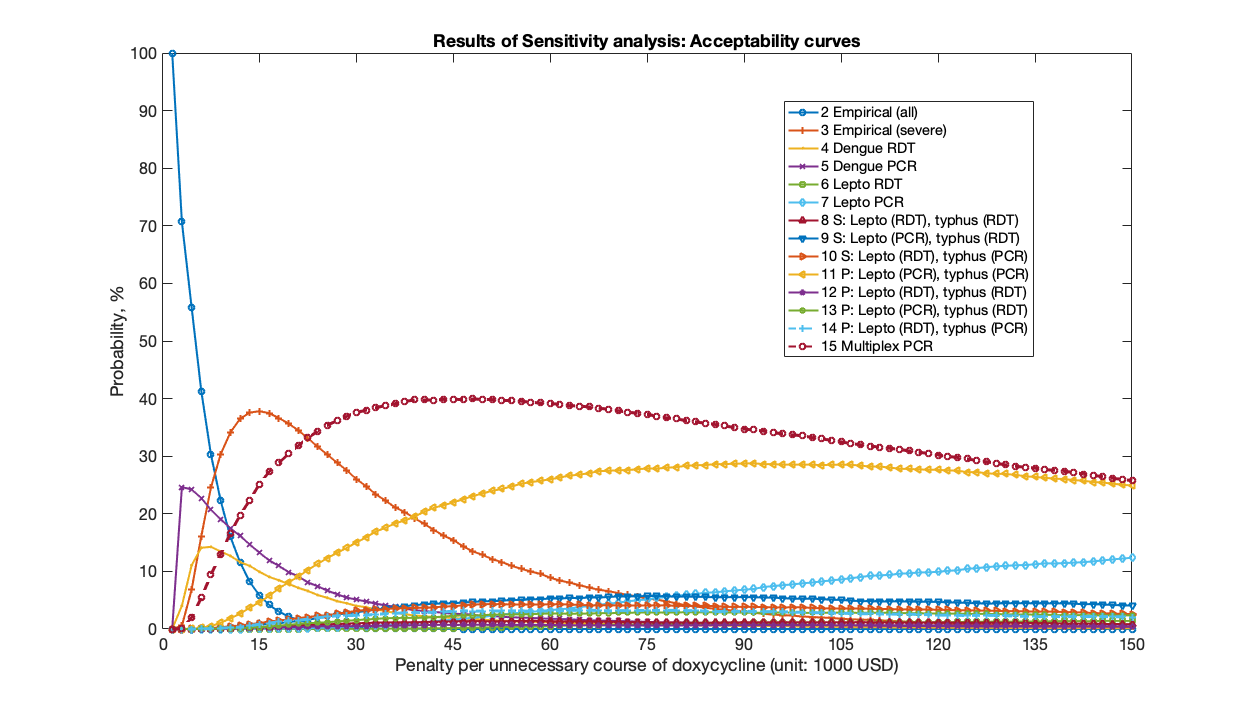

Supplement: S6 Fig — We fixed WTP = Thailand GDP per capita. The optimal strategy for a given penalty, is the strategy with the highest NMB value. (TIF) [file pone.0227409.s017.tif]

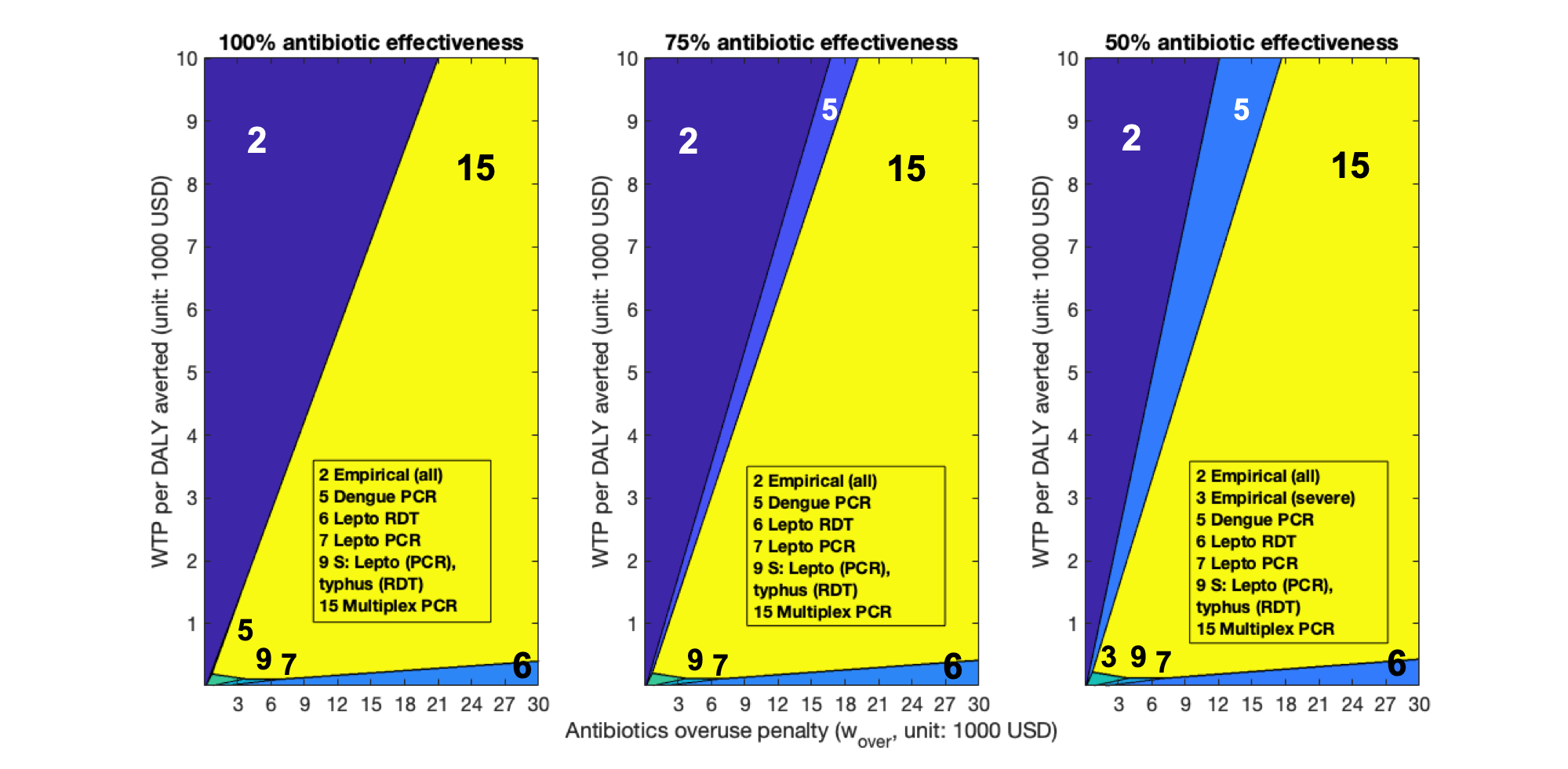

Supplement: S7 Fig — We vary willingness-to-pay (WTP) on the y-axis and penalty (wover) on the x-axis. (A): 100% antibiotic effectiveness (B): 75% antibiotic effectiveness (C): 50% antibiotic effectiveness. (TIF) [file pone.0227409.s018.tif]

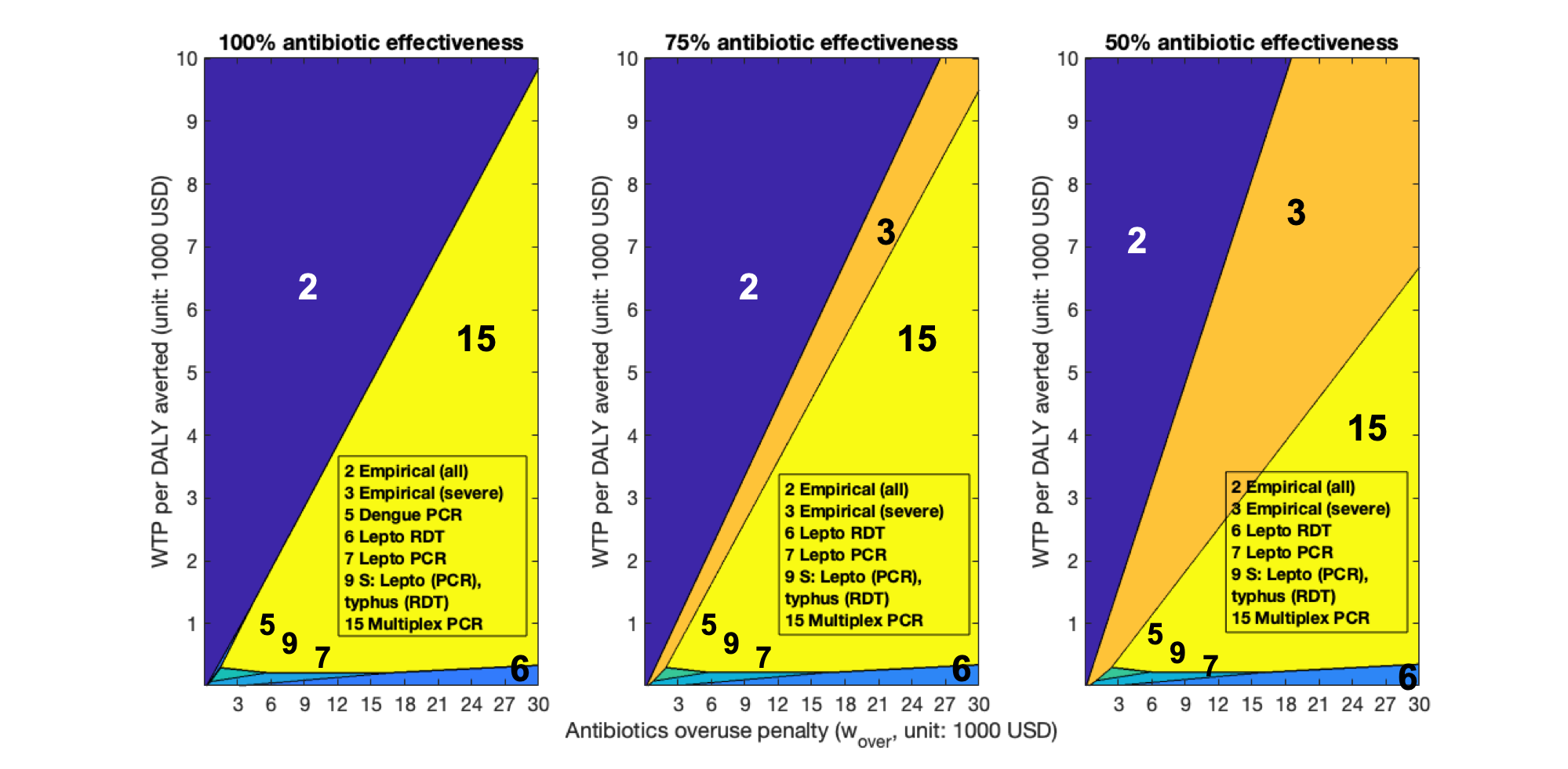

Supplement: S8 Fig — We vary willingness-to-pay (WTP) on the y-axis and penalty (wover) on the x-axis. (A): 100% antibiotic effectiveness (B): 75% antibiotic effectiveness (C): 50% antibiotic effectiveness. (TIF) [file pone.0227409.s019.tif]

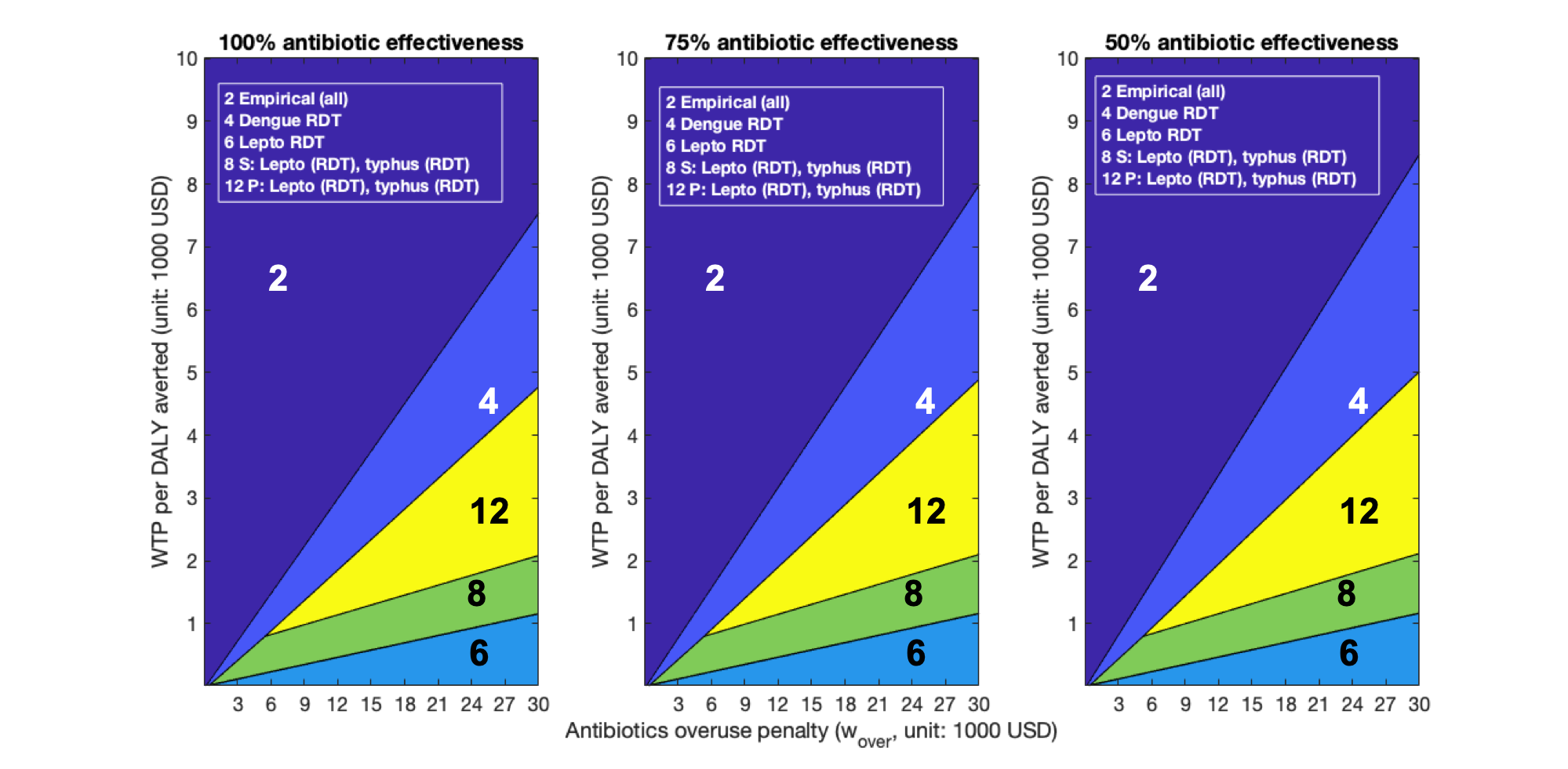

Supplement: S9 Fig — We vary willingness-to-pay (WTP) on the y-axis and penalty (wover) on the x-axis. (A): 100% antibiotic effectiveness (B): 75% antibiotic effectiveness (C): 50% antibiotic effectiveness. (TIF) [file pone.0227409.s020.tif]

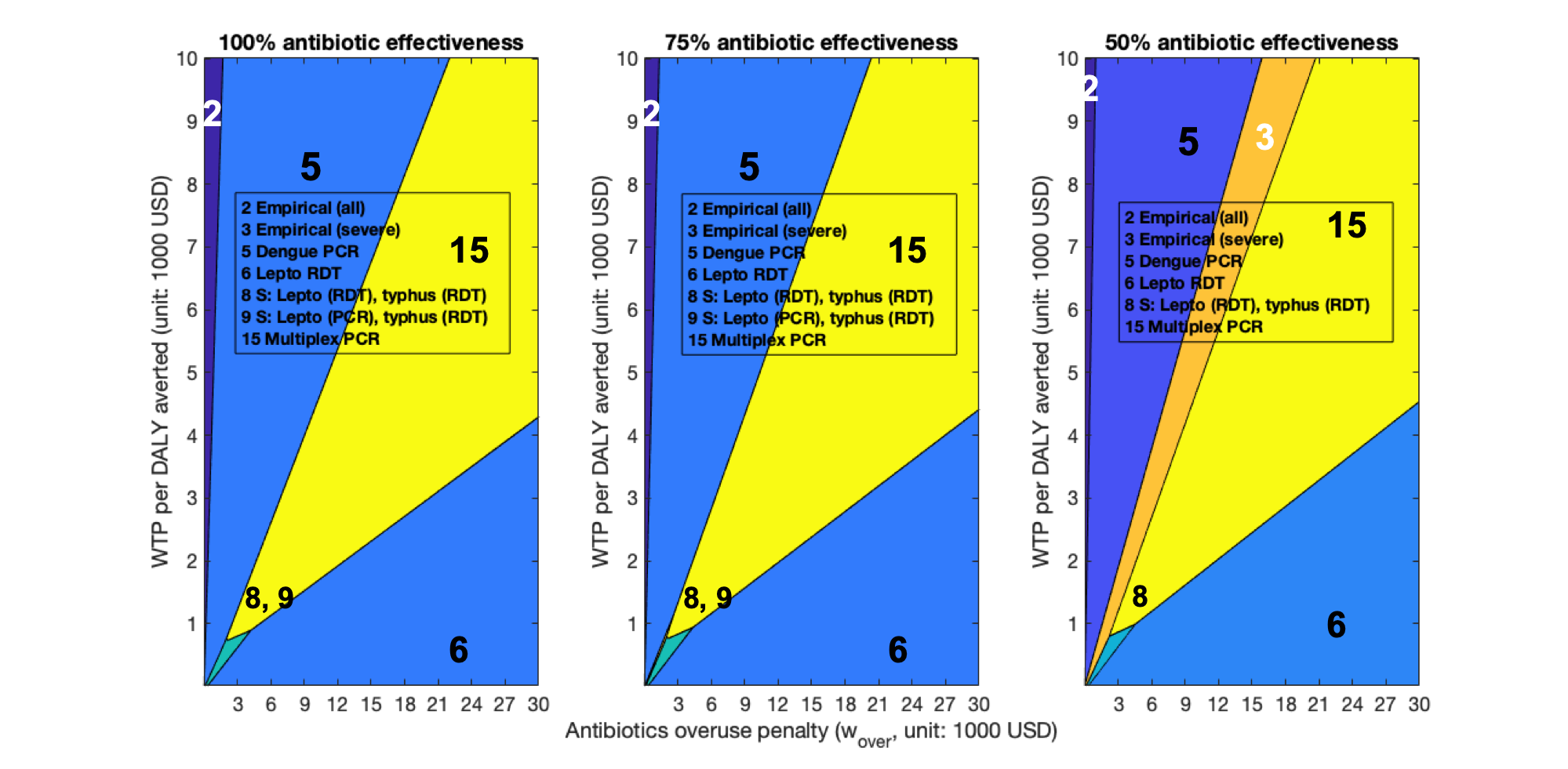

Supplement: S10 Fig — We vary willingness-to-pay (WTP) on the y-axis and penalty (wover) on the x-axis. (A): 100% antibiotic effectiveness (B): 75% antibiotic effectiveness (C): 50% antibiotic effectiveness. (TIF) [file pone.0227409.s021.tif]

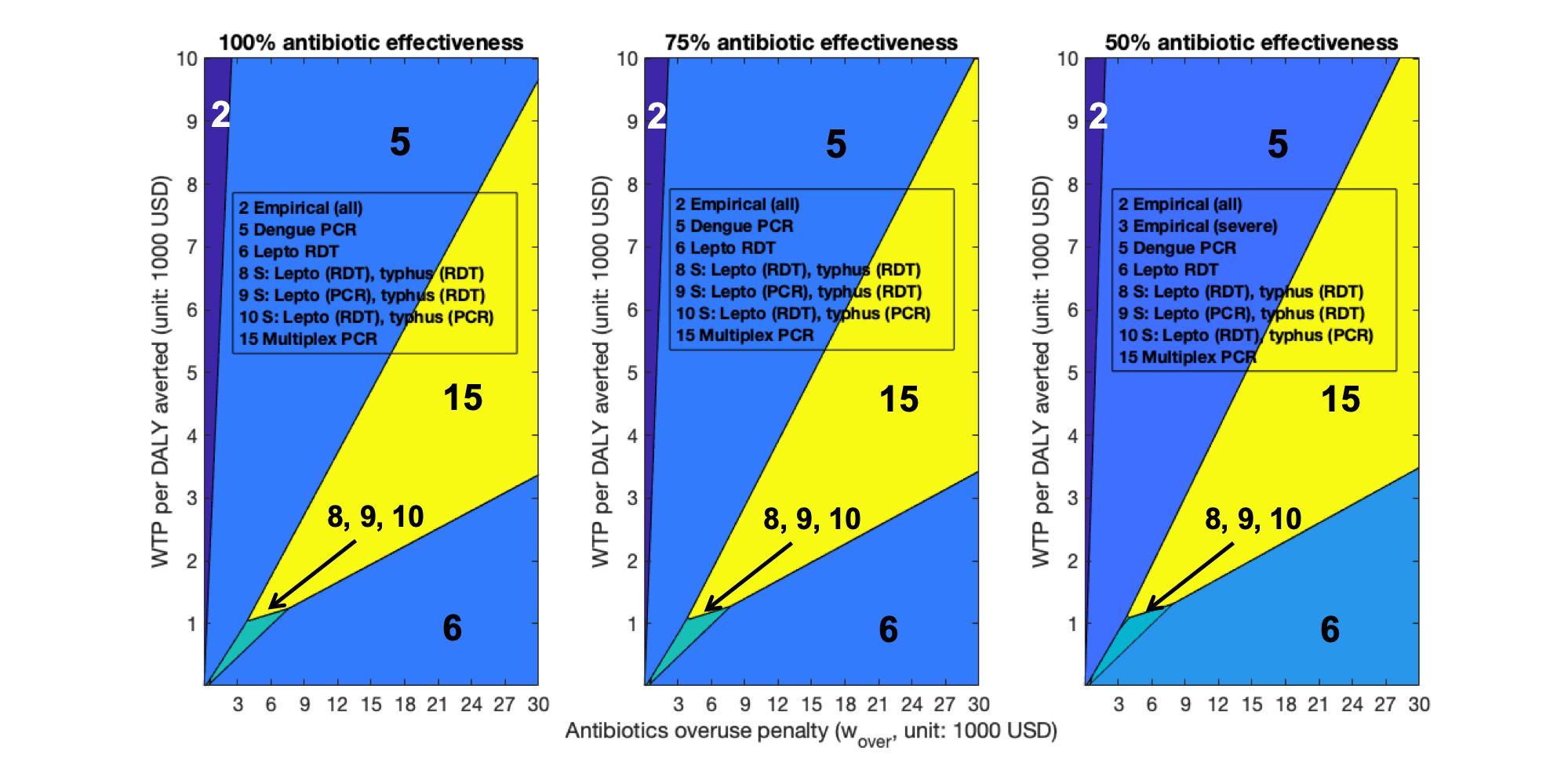

Supplement: S11 Fig — We vary willingness-to-pay (WTP) on the y-axis and penalty (wover) on the x-axis. (A): 100% antibiotic effectiveness (B): 75% antibiotic effectiveness (C): 50% antibiotic effectiveness. (TIF) [file pone.0227409.s022.tif]

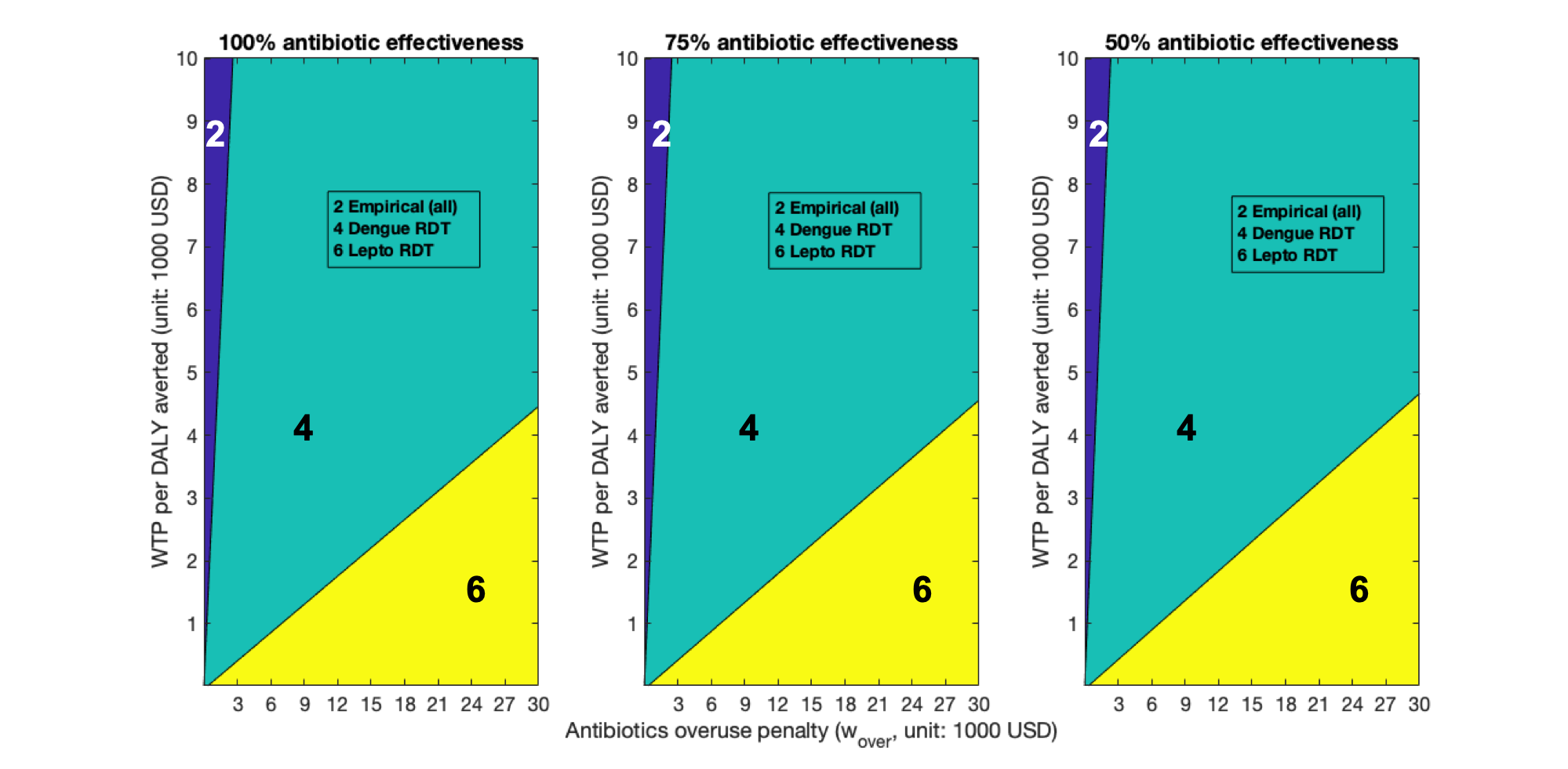

Supplement: S12 Fig — We vary willingness-to-pay (WTP) on the y-axis and penalty (wover) on the x-axis. (A): 100% antibiotic effectiveness (B): 75% antibiotic effectiveness (C): 50% antibiotic effectiveness. (TIF) [file pone.0227409.s023.tif]

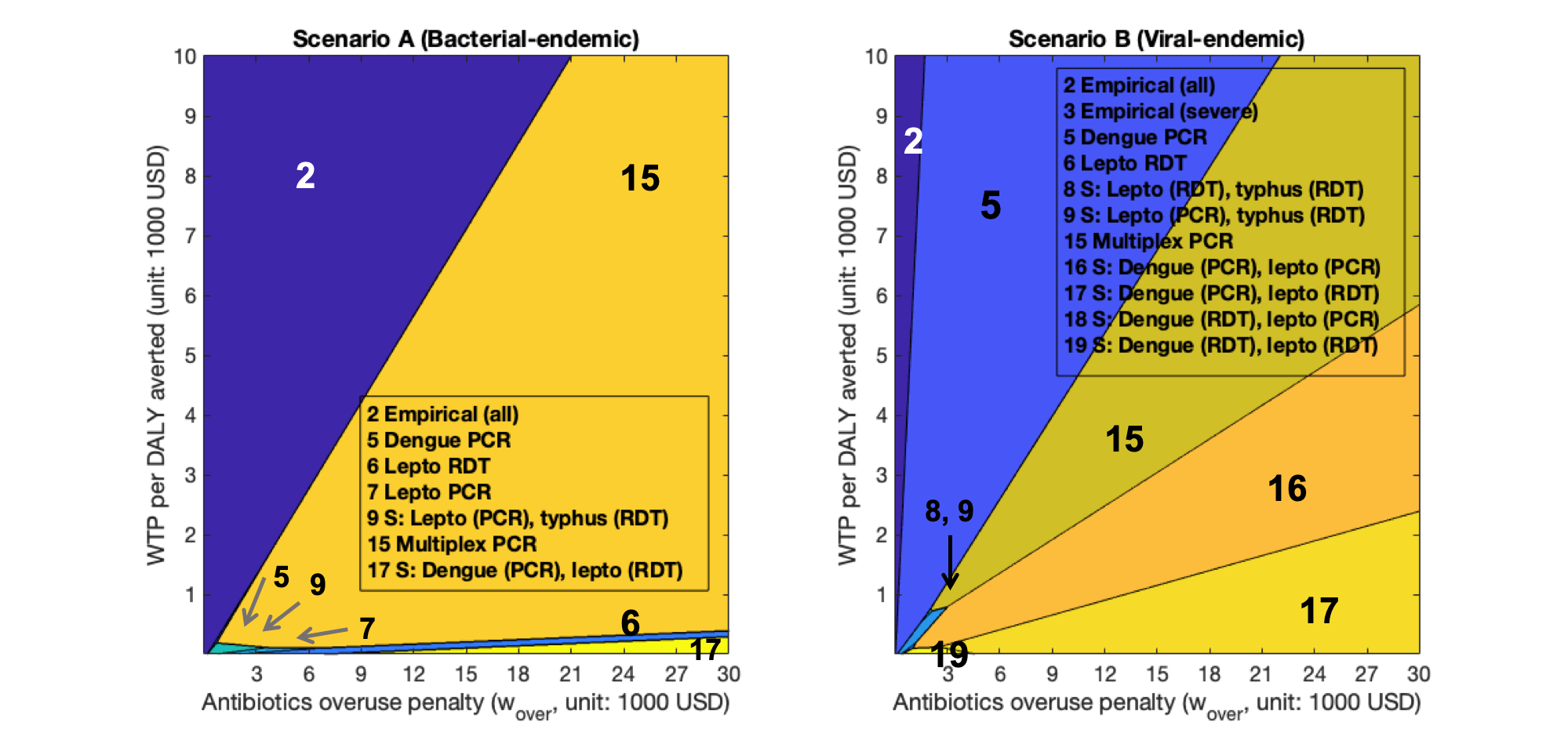

Supplement: S13 Fig — We vary willingness-to-pay (WTP) on the y-axis and penalty (wover) on the x-axis. (A): Bacterial-endemic Scenario A (B): Viral-endemic Scenario B. (TIF) [file pone.0227409.s024.tif]

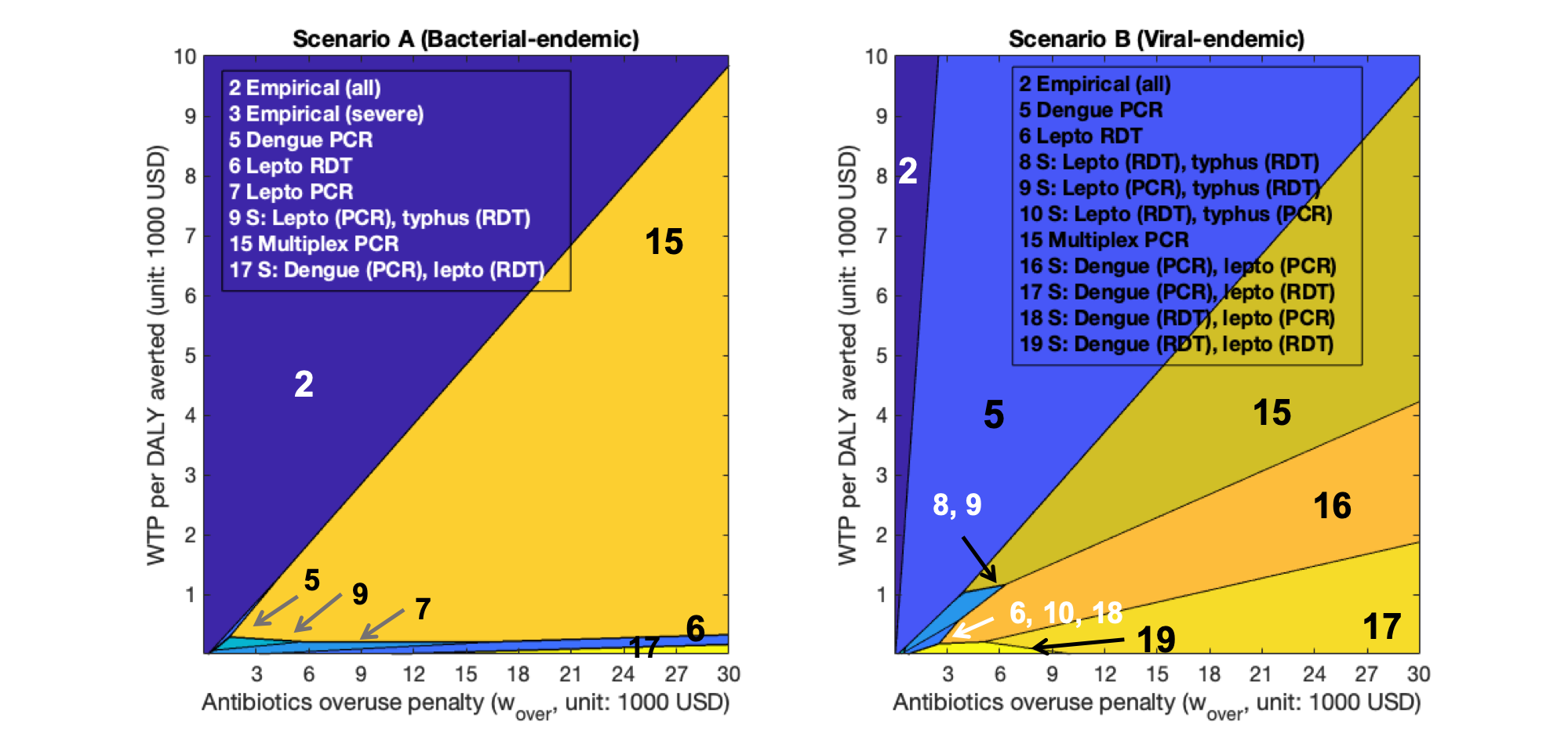

Supplement: S14 Fig — We vary willingness-to-pay (WTP) on the y-axis and penalty (wover) on the x-axis. (A): Bacterial-endemic Scenario A (B): Viral-endemic Scenario B. (TIF) [file pone.0227409.s025.tif]

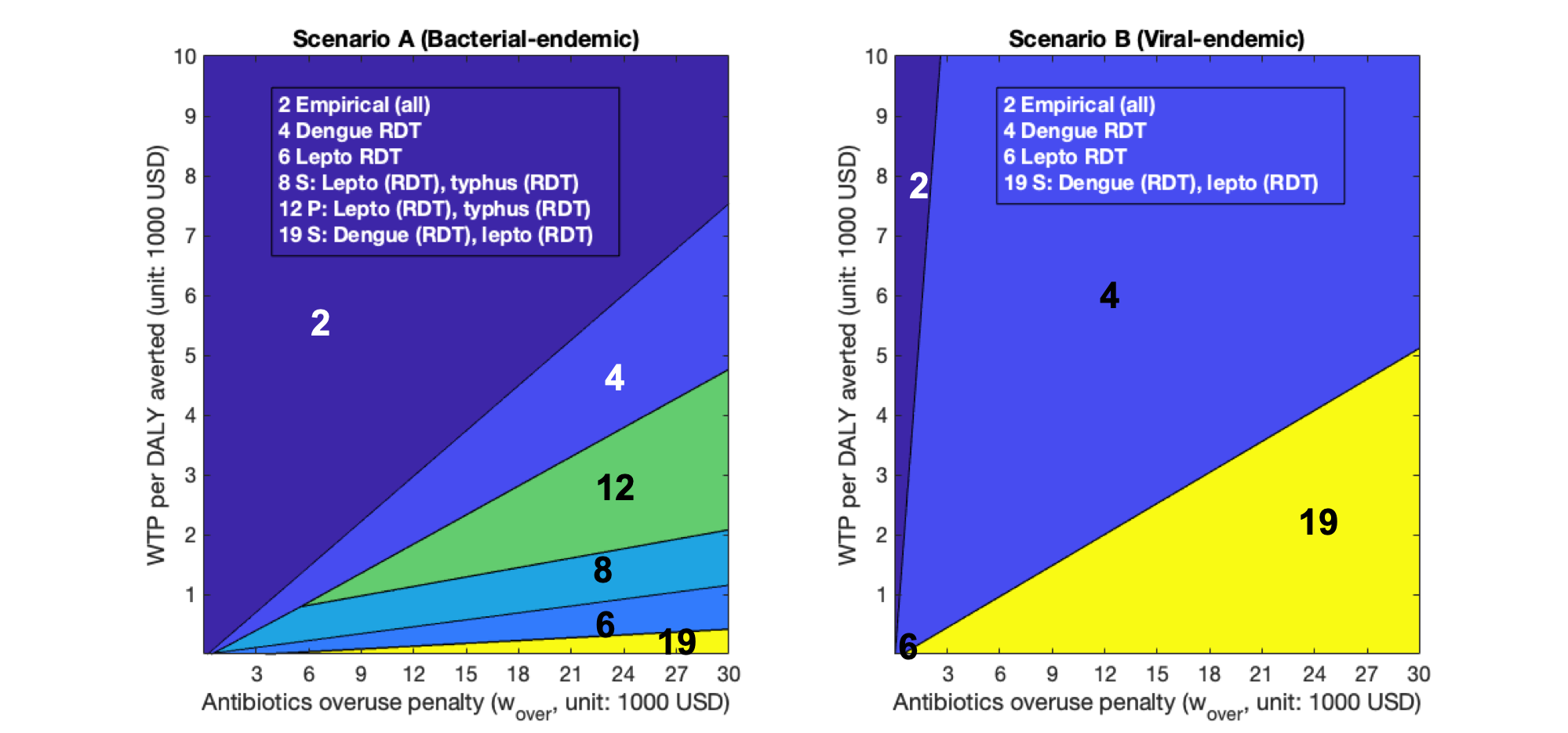

Supplement: S15 Fig — We vary willingness-to-pay (WTP) on the y-axis and penalty (wover) on the x-axis. (A): Bacterial-endemic Scenario A (B): Viral-endemic Scenario B. (TIF) [file pone.0227409.s026.tif]
